# Dual Role of Tamoxifen in Enhancing STING and CEACAM1 Expression to Prime a Favorable Tumor Microenvironment for Anti‐TIM3 Immunotherapy

**DOI:** 10.1002/advs.202503303

**Published:** 2026-08-17

**Authors:** Marvin Angelo E Aberin, Saurabh Singh, Soumya Chatterjee, Guang‐Zhi Sui, Yi‐Fu Wang, Ya‐Ting Lu, Yu‐Ling Lee, Kun‐Yuan Lin, Joy Khag, Ta‐Yu Liu, Shao‐Han Chang, Wai‐Mui Cheung, Hsiao‐Chin Hong, Ren‐Jun Hsu, Chen‐Yang Shen, Chia‐Wei Li, Weng‐Lang Yang, Yao‐Ming Chang, Shih‐Yu Chen, Chandan Guha, Shu‐Ping Wang

**Affiliations:** ^1^ Taiwan International Graduate Program in Molecular Medicine Academia Sinica and National Yang Ming Chiao Tung University Taipei Taiwan; ^2^ Institute of Biomedical Sciences Academia Sinica Taipei Taiwan; ^3^ Department of Radiation Oncology Albert Einstein College of Medicine New York USA; ^4^ Pharmacology Discovery Services Taiwan Ltd. New Taipei Taiwan; ^5^ Taiwan International Graduate Program in Molecular and Cellular Biology Academia Sinica and Graduate Institute of Life Sciences National Defense Medical Center Taipei Taiwan; ^6^ Department of Data Science Soochow University Taipei Taiwan; ^7^ Institute of Medical Science Tzu Chi University Hualien Taiwan; ^8^ Cancer Center, Hualien Tzu Chi Hospital Buddhist Tzu Chi Medical Foundation Hualien Taiwan; ^9^ Department of Pathology Albert Einstein College of Medicine New York USA

**Keywords:** breast cancer, CEACAM1/TIM3 blockade, cGAS/STING pathway, immunotherapy, RACK7/KDM5 histone demethylase complex, tamoxifen, type I interferons

## Abstract

Despite advancements in immune checkpoint blockade (ICB) therapy, breast cancer shows limited response to anti‐PD1/PD‐L1 treatments, emphasizing the need for alternative ICB targets. Here, we reveal that endocrine therapeutics, specifically tamoxifen, create an immunosuppressive yet primed tumor microenvironment conducive to anti‐TIM3 immunotherapy. Tamoxifen induces mitochondrial DNA damage and disrupts the RACK7/KDM5C histone demethylase complex, resulting in STING accumulation and activation of the type I interferon (IFN‐I) pathway, thereby fostering an immunogenic tumor microenvironment. However, tamoxifen also elevates CEACAM1 expression via a RACK7/KDM5C axis, driving T‐cell exhaustion and limiting tumor elimination. This dual effect of tamoxifen – promoting STING‐mediated immunogenicity while upregulating CEACAM1 expression – shapes the tumor‐immune microenvironment in both ER‐positive and ER‐negative tumors. Notably, combining anti‐TIM3 immunotherapy with tamoxifen mitigates its immunosuppressive effects, potentially enhancing ICB efficacy. Our findings highlight the therapeutic potential of integrating anti‐TIM3 immunotherapy with endocrine therapy to improve outcomes for breast cancer patients.

## Graphical abstract

Anti‐PD‐1/PD‐L1 blockade has revolutionized cancer immunotherapy, but is ineffective against endocrine‐treated (i.e., Tamoxifen), relapsed ER^+^ breast cancer (BC) patients. This study provides insight into the sub‐optimal response of ER^+^BCs to anti‐PD‐1/PD‐L1 blockade – highlighting the induction of STING and the CEACAM1/TIM3 axis after chronic Tamoxifen treatment – and suggesting an alternative approach using anti‐TIM3 or anti‐TIM3/PD‐1 combination therapy for relapsed ER^+^BCs.

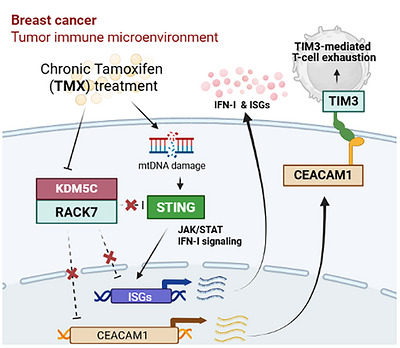

## 1 Introduction

Despite advancements in immunotherapy against various cancers, breast cancer still retains poor response to immune checkpoint blockade (ICB) therapy [1, 2]. Current strategies such as humanized monoclonal antibodies (mAbs) targeting programmed cell death‐1 (PD1; e.g., pembrolizumab and nivolumab) exhibit limited clinical benefit. Numerous studies point to the poor tumor immunogenicity of most breast cancers, often referred to as “non‐inflamed” or “cold” tumors, in contrast to “hot” tumors that respond well to ICB [3]. Additionally, the poor infiltration of T‐lymphocytes into the tumor microenvironment largely contributes to the observed poor response [4, 5, 6]. Several other biomarkers are prognostic of the effectiveness of ICB therapy and are commonly associated with hot tumors. One such biomarker is tumor mutational burden (TMB), defined as the number of somatic mutations (mt) per megabase (Mb) of analyzed genomic sequence (mt/Mb). Higher TMB status correlates with enhanced neoantigen presentation and better survival rates in ICB‐treated patients [7]. Activated antigen processing and presentation is another phenotype associated with hot tumors and is closely correlated with enhanced tumor recognition and targeting [8].

Modulating the breast cancer tumor microenvironment to recruit more tumor‐infiltrating lymphocytes (TILs) is a key factor in transforming a “cold” breast tumor into a “hot” one. Consequently, the immunomodulatory effects and clinical benefits of combining adjuvant chemotherapy or radiotherapy with immunotherapeutic agents in breast cancer patients have been evaluated in numerous clinical trials. Notably, favorable benefit‐risk assessments from phase III clinical trials, such as IMpassion130, KEYNOTE‐355 and KEYNOTE‐522, led to the approval of the immune‐chemotherapy combinations with pembrolizumab or atezolizumab (targeting programmed death‐ligand 1, PD‐L1) by the US Food and Drug Administration (FDA) for patients with advanced or metastatic triple‐negative breast cancer (TNBC) [9, 10, 11, 12]. However, owing to the limited response to ICB, the immunotherapeutic option is currently unavailable to estrogen receptor‐positive (ER^+^) patients, who account for approximately 75% of all breast cancer diagnoses.

Although ER^+^ breast cancer patients generally have favorable prognosis, significant relapse rates remain a critical clinical issue to be addressed. The common therapeutic options for ER^+^ breast cancer are to inhibit tumor cell proliferation by targeting ER signaling with selective ER modulators (e.g., tamoxifen), aromatase inhibitors (a.k.a. AIs; e.g., anastrozole, letrozole, exemestane), and the selective ER degrader, fulvestrant [13]. Among these anti‐ER signaling therapeutics, tamoxifen has been employed in adjuvant treatment of ER^+^ breast cancer for several decades. Although newer drugs such as AIs exhibit better effectivity among post‐menopausal women, they have limited utility for premenopausal patients and can exhibit unfavorable safety profiles [14, 15]. This makes tamoxifen the standard therapeutic agent for ER^+^ breast cancer, especially among lower‐risk and premenopausal patients [16]. However, resistance to tamoxifen therapy is observed in around 30% of patients, with a loss of or reduced ER levels considered a primary mechanism of resistance [13, 17].

Interestingly, early clinical observations reveal that tamoxifen is capable of inducing regression of some tumors lacking ER expression, whereas tamoxifen is also capable of increasing host resistance against cancer in an ER‐independent mechanism [18, 19, 20, 21]. These findings highlight the non‐canonical, cytotoxic effects of tamoxifen, which include modulation of the immune microenvironment [22, 23, 24], impairment of mitochondrial function [25], and perturbation of DNA damage response (DDR) pathways [26, 27]. The DNA‐damaging potential of tamoxifen may result in the leakage of single‐stranded DNA (ssDNA) and its accumulation in the cytosol of ER^+^ breast cancer cells, thereby activating type I interferon‐dependent interferon‐stimulated gene (ISG) expression [28]. Interestingly, high levels of ISG expression in breast cancer patients are associated with increased TIL expression signature but correlated with worse outcomes in patients treated with adjuvant tamoxifen [28, 29].

Type I IFNs are critical regulators of the innate immune system, which is important for inducing durable and protective T‐cell responses to infection, and has been progressively recognized as a target for cancer immunotherapy [30]. Innate immune responses are initiated by pattern recognition receptors (PRRs) that can sense damage‐associated molecular patterns (DAMPs) and pathogen‐associated molecular patterns (PAMPs) [31]. Cyclic GMP‐AMP synthase (cGAS) has emerged as a key PRR for the recognition of cytosolic DNA and the activation of innate immunity [32, 33]. Upon DNA binding, cGAS becomes enzymatically active and thereby synthesizes a second messenger, cGAMP. cGAMP binds to stimulator of interferon genes (STING), which in turn triggers activation of downstream effectors of the cGAS/STING pathway, TANK‐binding kinase 1 (TBK1) and interferon regulatory factor 3 (IRF3) [34]. Activated IRF3 translocates into the nucleus, leading to increased transcription of genes encoding type I IFNs. Type I IFNs (e.g., IFN‐α, β) then initiate ISG induction via the IFN receptor and the downstream JAK/STAT signaling [35]. Given that the cGAS‐STING and type I IFN (IFN‐I) signaling pathways have been shown to both stimulate and suppress immune responses [36, 37], and can also exert immune‐independent effects [38], the actual role of tamoxifen in modulating these pathways and shaping the breast cancer tumor microenvironment remains obscure.

RACK7 (aka ZMYND8) is a scaffold protein that forms a transcriptional corepressor complex with the histone H3K4 KDM5 demethylase family members (KDM5A‐D) [39, 40]. Among these, the RACK7/KDM5C complex can inhibit oncogene‐associated enhancer hyperactivation and suppress tumor development and malignancy in breast cancer [41, 42]. Interestingly, KDM5C can also target the *STING* promoter, repressing *STING* expression and negatively impacting the cGAS−STING−TBK1−IRF3 signaling cascade [43]. Pharmacological inhibition of KDM5C enzymatic activity activates *STING* expression and triggers an interferon response in breast cancer cells [43]. The current findings highlight the critical role of the RACK7/KDM5C complex in controlling the cGAS−STING−TBK1−IRF3 axis. However, it remains unclear whether this demethylase complex and/or the cGAS−STING−TBK1−IRF3 axis influence breast cancer patients’ response to endocrine therapy, with or without ICBs, and what the possible underlying mechanisms could be.

In this study, we investigate the immunomodulatory effects of tamoxifen in breast cancer, with a specific emphasis on its impact within the “cold” and immune‐suppressed tumor microenvironment. Our findings uncover an additional mechanism of tamoxifen resistance in activating the cGAS/STING pathway by inducing mitochondrial DNA (mtDNA) damage, disrupting the stability of the RACK7/KDM5C histone demethylase complex, and increasing STING protein accumulation. Interestingly, this tamoxifen‐mediated epigenetic and immunomodulatory axis not only activates IFN‐I signaling, but also contributes to T‐cell exhaustion by promoting *CEACAM1* transcription and subsequent CEACAM1/TIM3 interactions. Collectively, these events contribute to the establishment of an immunosuppressive yet ready‐to‐fire tumor microenvironment for anti‐CEACAM1/TIM3 immunotherapy, presenting a promising target for effective tumor eradication interventions.

## 2 Results

### 2.1 Chronic Tamoxifen Treatment Triggers Lymphocyte‐Attractive IFN‐I Signaling and Concurrently Induces the Expression of Ligands for the T‐Cell Inhibitory Receptor TIM3

**FIGURE 1:**
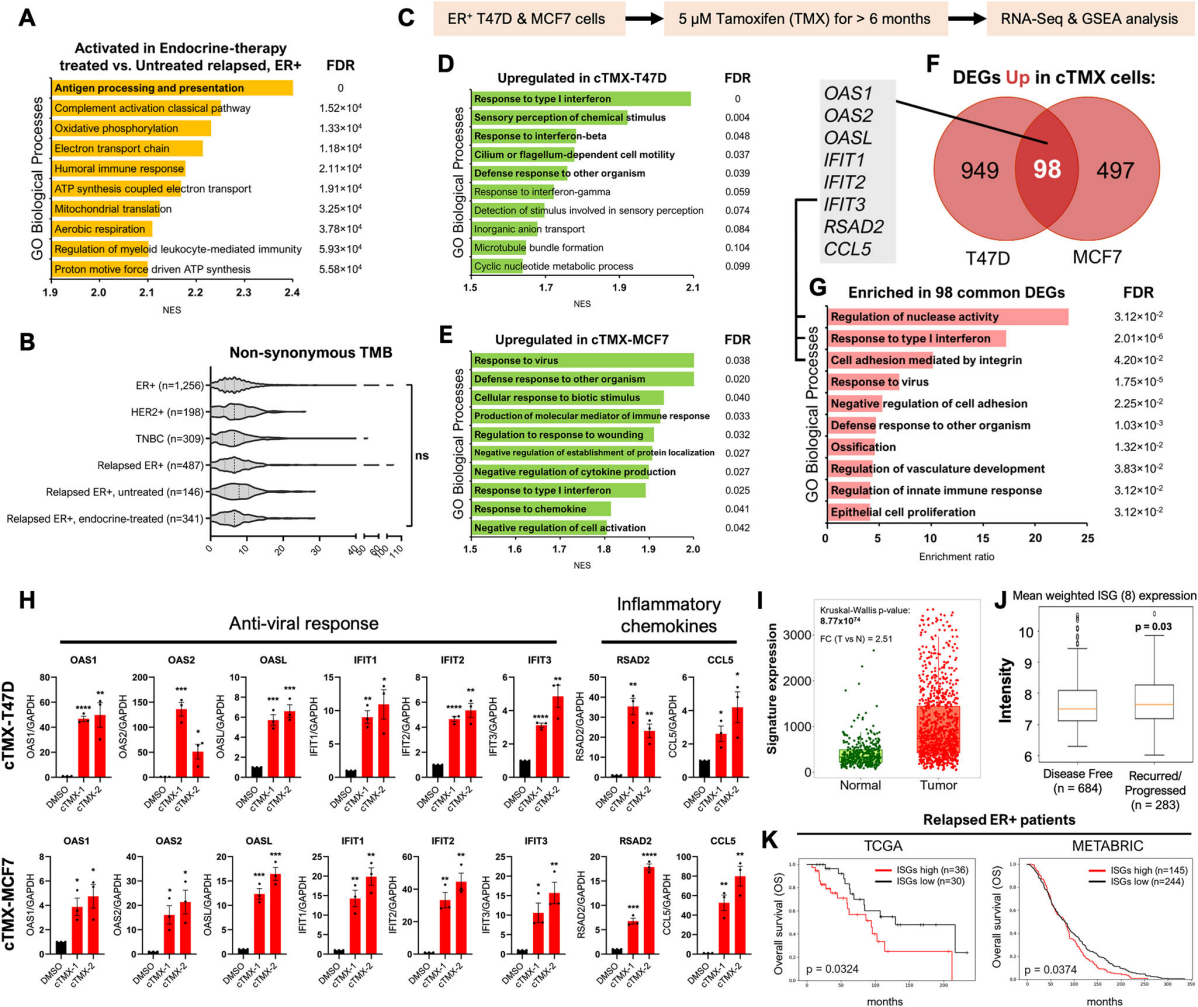
Clinical patient database and cell line‐based transcriptomic analyses reveal lymphocyte‐attractive phenotypes in endocrine therapy‐treated conditions. **A**. GSEA analysis of microarray expression data in tumor biopsies of relapsed ER^+^ patients receiving endocrine therapy treatment compared with those who do not receive endocrine therapy (NES > 0, FDR < 0.05). Data were obtained from the cBioPortal webtool (Breast cancer dataset: METABRIC, Nature 2012 [47] & Nature Communications 2016 [46], *n* = 2,509) and re‐analyzed using the GSEA ver4.3.2 software. Data is presented as the normalized enrichment score (NES) for each pathway and their corresponding false discovery rate (FDR). **B**. Distribution of non‐synonymous TMB across different breast cancer subtypes (ER^+^, HER2^+^, TNBC) and different status of endocrine therapy in overall or relapsed ER^+^ patients, respectively. Data is presented as distribution of TMB and analyzed using the Kruskal‐Wallis test (*p* = 0.1003). **C**. Schematic of chronic tamoxifen treatment of ER^+^ T47D and MCF7 cells. **D** and **E**. GSEA analysis of upregulated DEGs in cTMX‐T47D (D) or cTMX‐MCF7 (E) cells compared with the respective control (DMSO‐treated) cells. Data is presented as the NES for each pathway and their corresponding FDR. **F**. Venn diagram of the unique (T47D, *n* = 949; MCF7, *n* = 497) and the 98 common upregulated DEGs in the cTMX cells. Genes exhibiting log‐fold change (logFC) > 1.5 and *p*‐value < 0.05 from the RNA‐Seq analysis are considered upregulated. **G**. Over‐Representation Analysis (ORA) indicating top 10 pathways enriched in the 98 common upregulated genes between cTMX‐T47D and cTMX‐MCF7 clones. The grey box indicates 8 ISGs that were consistently upregulated in the cTMX clones. Data presented as the NES for each pathway and their corresponding FDR. **H**. RT‐qPCR analysis showing relative mRNA expression levels of the representative ISGs in two independent cTMX‐T47D and MCF7 clones compared with the control cells. Data is presented as mean log2FC ± SEM, *n* = 3, *p*‐values are calculated using one‐way ANOVA followed by Tukey post‐hoc test, *p < 0.05, **p < 0.005, ***p < 0.0005, ****p < 0.0001. **I**. Clinical patient dataset analysis using the TNMplot webtool indicating a high co‐expression pattern of these 8 ISGs in invasive breast carcinoma tumor tissues compared to the normal tissues. Data is presented as distribution of ISG‐signature expression and analyzed using the Kruskal‐Wallis test, *p* < 0.0001. **J**. Clinical patient dataset analysis using the CTGS webtool showing a high co‐expression pattern of these 8 ISGs in recurred/progressed ER^+^ patients compared to those who are disease‐free. Data is presented as box plots of ISG‐signature expression and analyzed using the Kruskal‐Wallis test. **K**. Kaplan‐Meier survival analysis showing that high co‐expression of these 8 ISGs correlates with worse overall survival of relapsed ER^+^ patients from two independent public databases (TCGA and METABRIC). TMB, tumor mutational burden; ER^+^, ER‐positive; HER2^+^, HER2‐positive; TNBC, triple negative breast cancer; cTMX, chronic tamoxifen; DEGs, differentially expressed genes.

The success of anti‐PD1/PD‐L1 immunotherapy in TNBC patients has prompted similar investigations of ICB therapy for ER^+^ patients [9]. However, interim results indicate poor to limited clinical benefit, particularly among relapsed patients who have undergone prior endocrine therapy [3, 44]. To explore why endocrine therapy‐treated, ER^+^ breast cancer patients respond poorly to ICB, we examined biomarkers that are associated with “hot” tumors by retrieving publicly‐available microarray and non‐synonymous TMB data from the METABRIC dataset, and reanalyzing them using cBioPortal [45, 46, 47]. Gene set enrichment analysis (GSEA) results showed that among relapsed ER^+^ patients, those who received endocrine therapy exhibit activated “antigen processing and presentation” compared to those who did not receive endocrine therapy (Figure 1A, Supplementary Figure S1A). Additionally, these patients demonstrate a similar TMB profile to ICB‐responsive TNBCs (Figure 1B, Supplementary Figure S1B). Interestingly, high TMB status is correlated with reduced overall survival (OS) among relapsed ER^+^ patients who received endocrine therapy (Supplementary Figure S1I), a prognostic effect not observed among patients who did not receive endocrine therapy (Supplementary Figure S1H). Both the activated antigen‐presentation phenotype and TNBC‐like TMB profile of these endocrine therapy‐treated ER^+^ patients suggest an immunogenic tumor microenvironment that promotes “hot” tumor immunity. However, this did not correlate with better clinical outcomes (Supplementary Figure S1I). More importantly, it cannot explain the poor response of relapsed, ER^+^ patients to ICB therapies. Instead, these findings strongly suggest an unexplored compensatory role of endocrine therapies that negatively impact anti‐tumor immunity.

To investigate how endocrine therapy affects patients’ response to ICB therapies and the underlying mechanisms, we selected tamoxifen as a representative endocrine therapy, given that tamoxifen is recommended as the first‐line treatment for pre‐menopausal, low‐risk, and early ER^+^ breast cancer patients [16]. We cultured ER^+^ T47D and MCF7 breast cancer cells in a clinically relevant concentration of 5 µM tamoxifen for over 6 months, and subjected these chronically tamoxifen‐treated cells (designated cTMX‐T47D and cTMX‐MCF7 cells) to high‐throughput RNA sequencing (RNA‐seq) (Figure 1C). We selected 5 µM tamoxifen for this study based on prior reports indicating that tamoxifen and its metabolites can accumulate in cancer tissues at concentrations ranging from 0.56 to 6.88 µM, which is considerably higher than the average steady‐state plasma levels [48]. Pathway enrichment analysis (PEA) and GSEA of the RNA‐seq data revealed upregulation of several immunoregulatory pathways, including IFN‐I signaling and innate immunity, in both cTMX‐T47D and MCF7 cell lines (Figure 1D and E, Supplementary Figure S2A, S2D, and S2E). Similar pathways were enriched among the 98 commonly upregulated genes in these cTMX cell lines (Figure 1F and G). To validate our RNA‐seq results, we performed RT‐qPCR assays on two independent cTMX‐T47D and MCF7 cell clones, assessing the expression of eight ISGs. These ISGs, including pro‐inflammatory chemokines (e.g., RSAD2, CCL5) and canonical anti‐viral ISGs (e.g., OAS1, OAS2, OASL, IFIT1, IFIT2, IFIT3), were consistently upregulated in the cTMX clones (Figure 1H). Analyzing an RNA‐seq dataset from in vivo T47D xenograft models treated with tamoxifen (GSE80619) [49], we confirmed that tamoxifen induces IFN‐I signaling within the tumor microenvironment (Supplementary Figure S2F). To further validate these findings and assess the effects of tamoxifen at single‐cell resolution across different cell populations, we analyzed a single‐cell RNA‐seq (scRNA‐seq) dataset of patient‐derived breast tumor biopsies treated ex vivo with tamoxifen (dbGaP accession# phs003186.v1.p1). Notably, we consistently observed selective activation of ISGs (e.g., IFIT1, IFIT2, IFIT3, and RSAD2) in tumor cells, but not in stromal cells, fibroblasts, epithelial non‐tumor cells, or immune cells (including T and B cells) (Supplementary Figure S3A–C). These findings suggest that although tamoxifen exerts both ER‐dependent and ER‐independent effects in breast cancer, its predominant activity within the tumor microenvironment may be mediated through ISG activation in tumor cells.

We next investigated the weighted mean expression of these ISGs and their prognostic effects in ER^+^ breast cancer patients. Our analysis of the TNMplot database [50] revealed a higher co‐expression pattern of these ISGs in breast tumor tissues compared to normal tissues (Figure 1I). Additionally, using the CTGS webtool [51], we found that these ISGs exhibit elevated co‐expression in relapsed ER^+^ patients who received endocrine therapy, as opposed to those who are disease‐free (Figure 1J), in agreement with our in vitro results. Our findings suggest that endocrine therapy creates an ISG‐associated, inflamed tumor microenvironment that is widely considered synergistic to anti‐tumor immunity [32]. Interestingly, however, high expression of this 8‐ISG signature is significantly correlated with adverse outcomes in relapsed ER^+^ patients (Figure 1K). This strongly suggests that although endocrine therapy promotes lymphocyte infiltration through the induction of ISGs, it also likely induces an unrecognized immunosuppressive mechanism that overpowers the effect of ISGs and ultimately contributes to worse patient outcomes.

**FIGURE 2:**
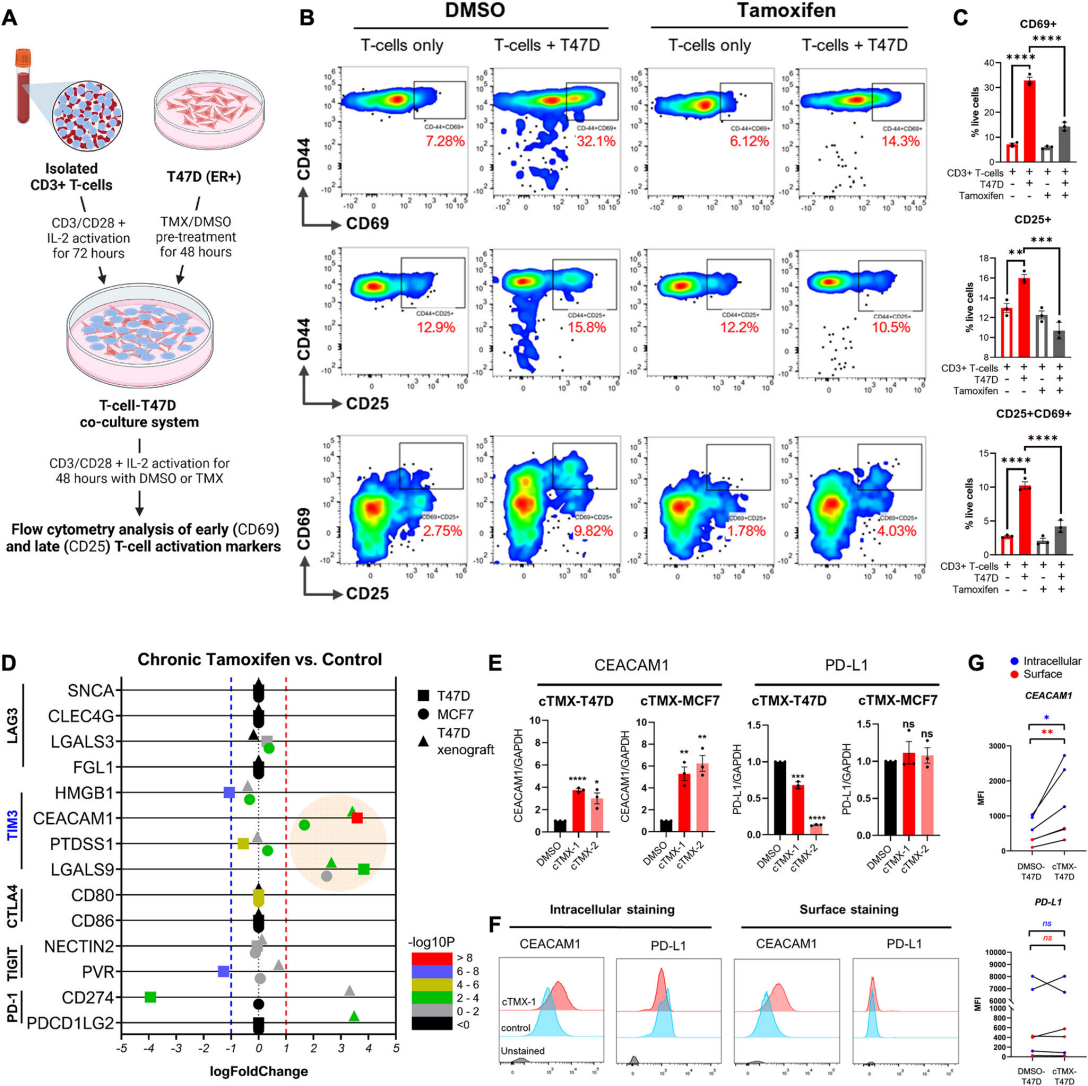
Chronic tamoxifen treatment upregulates the alternative T‐cell inhibitory ligand CEACAM1. **A**. Schematic of in vitro co‐culture experiments between isolated CD3^+^ T‐cells and T47D cells under DMSO/tamoxifen (TMX)‐treated conditions. **B**. Representative density plots showing percentages of CD44^+^CD69^+^, CD44^+^CD25^+^, and CD25^+^CD69^+^ T‐cells in the different treatment groups. **C**. Quantification of percent CD44^+^CD69^+^, CD44^+^CD25^+^, and CD25^+^CD69^+^ populations. Data is presented as mean % of live cells ± SEM, *n* = 3, *p*‐values are calculated using one‐way ANOVA followed by Tukey post‐hoc test, *p < 0.05, **p < 0.005, ***p < 0.0005, ****p < 0.0001. **D**. Differentially expressed genes (DEGs) encoding well‐known T‐cell inhibitory ligands (as indicated) in two independent cTMX‐T47D (square) and MCF7 (circle) clones compared with the control (DMSO‐treated). The triangle shows the indicated DEGs in T47D mouse xenografts treated with tamoxifen compared with the control (GSE80619). The corresponding T‐cell inhibitory receptors are shown on the far left. Data is presented as logFC of the T‐cell inhibitory ligands from the RNA‐seq analysis. **E**. RT‐qPCR analysis showing relative mRNA expression levels of CEACAM1 and PD‐L1 in two independent cTMX‐T47D and MCF7 clones compared with the control (DMSO‐treated). Data is presented as mean log2FC ± SEM, *n* = 3, *p*‐values are calculated using one‐way ANOVA followed by Tukey post‐hoc test, *p < 0.05, **p < 0.005, ***p < 0.0005, ****p < 0.0001. **F** and **G**. Representative flow cytometry and quantification results of intracellular and surface‐expressed CEACAM1 and PD‐L1 in control (DMSO‐treated) and cTMX‐T47D cells. Quantification data is presented as paired mean fluorescence intensity (MFI) per independent replicate, *n* = 3, *p*‐values are calculated using paired *t*‐test *p < 0.05, **p < 0.005.

Given the non‐specific, immunomodulatory functions of tamoxifen, we hypothesize that this unrecognized mechanism likely acts through the exhaustion of critical tumor‐killing factors in the tumor microenvironment, specifically cytotoxic T‐lymphocytes (CTLs). To illustrate this CTL‐exhaustive effect, we established a T47D/T‐cell co‐culture system (Figure 2A). In this setup, human CTLs were isolated from primary peripheral blood mononuclear cells (PBMCs) and activated using IL‐2 and CD3/CD28 T‐cell activators. These CTLs were then co‐cultured with T47D cells pre‐treated with tamoxifen or the vehicle control (DMSO). After 72 hours of co‐culture, CTL activation was assessed by flow cytometric monitoring of the well‐known CTL surface markers CD25, CD44, and CD69, which are enriched in activated CTLs upon CD3/CD28 stimulation [52, 53, 54] (Supplementary Figure S4A and S4B). Our data demonstrated that under control DMSO conditions, CTLs undergo a dramatic expansion of CD69^+^, CD25^+^, and double‐positive CD25^+^CD69^+^ populations (Figure 2B and C, red bars), consistent with findings that tumor cells can stimulate T‐cell activation via antigenic engagement and stimulation. In stark contrast, the expansion of activated CTL populations was significantly hampered when CTLs were co‐cultured under tamoxifen‐treated conditions (Figure 2B and C, gray bars). Notably, tamoxifen treatment of CTLs in the absence of T47D cells appear to have minimal effects on CTL‐activation (see CD3^+^ T‐cells only group). This strongly suggests that the tamoxifen‐induced inactivation of CTLs likely occurs through tumor‐intrinsic mechanisms, possibly involving T‐cell exhaustion pathways. This prompted us to investigate whether tamoxifen differentially affects the tumor expression of well‐known immune checkpoint molecules.

Our examination of the differentially expressed genes (DEGs) in cTMX cells (Supplementary Figure S2B and S2C) revealed no significant alterations in genes related to most well‐known immune checkpoint molecules (Figure 2D) [2]. Surprisingly, CEACAM1 and Galectin‐9 (LGASL9), two primary ligands for the non‐classical immune checkpoint molecule TIM3 [55], were upregulated in both cTMX‐T47D and cTMX‐MCF7 cells. CEACAM1 serves as a T‐cell inhibitory ligand expressed on the surface of tumor cells [56]. This enables it to interact with its corresponding inhibitory receptor, TIM3, on T‐lymphocytes, ultimately resulting in T‐cell exhaustion [55]. While PD‐L1 (CD274) expression remained unchanged following chronic tamoxifen exposure in MCF7 cells (cTMX‐MCF7), we observed a reduction in CD274 expression in cTMX‐T47D cells. These findings were further validated by RT‐qPCR and flow cytometry analyses using anti‐PD‐L1 and CEACAM1 antibodies (Figure 2E–G). These results indicate that PD‐L1 is differentially regulated in these two ER^+^ breast cancer cell lines, likely through mechanisms that are not solely dependent on tamoxifen treatment.

To determine whether MCF7 and T47D cells exhibit comparable T‐cell inhibitory effects following chronic tamoxifen exposure, we performed T‐cell co‐culture assays using two independent cTMX‐T47D and cTMX‐MCF7 clones, with or without tamoxifen treatment. T cells co‐cultured with control cells (DMSO‐treated T47D or MCF7) displayed increased frequencies of CD25^+^, CD69^+^, and/or CD25^+^CD69^+^ populations compared with non‐co‐cultured T cells, indicating robust tumor cell‐mediated T‐cell activation. In contrast, both cTMX‐T47D and cTMX‐MCF7 cells suppressed this activation (Supplementary Figure S4C and S4D), demonstrating that chronic tamoxifen exposure confers immunosuppressive properties in both cell lines. Notably, the suppressive effect was more pronounced in cTMX‐T47D cells than in cTMX‐MCF7 cells. These results suggest that tamoxifen‐induced immunosuppression may be mediated through distinct mechanisms in different cellular contexts. In T47D cells, this effect is more closely associated with modulation of the CEACAM1‐TIM3 axis, whereas in MCF7 cells, persistent PD‐L1 expression may play a dominant role in driving T‐cell inhibition. Further analysis of CD274 and CEACAM1 in tamoxifen‐treated T47D xenografts (GSE80619) [49] revealed elevated levels of these ligands for both PD1 and TIM3 in the tamoxifen‐modulated tumor microenvironment (Figure 2D, triangle). We speculate that the observed differences in PD‐L1 (CD274) expression levels between cell‐based assays and in vivo xenograft models may be attributed to a positive feedback loop between tumor cells and intra‐tumoral lymphocytes. In this loop, the activation of IFN‐I signaling may trigger CD8^+^ TILs to release IFN‐γ, which, in turn, regulates PD‐L1 upregulation in tumor cells [38]. However, PD‐L1 upregulation in the tamoxifen‐modulated tumor microenvironment may not fully account for the unfavorable outcomes observed in tamoxifen‐treated ER^+^ patients, as evidenced by the limited response to anti‐PD1/PD‐L1 immunotherapy in clinical settings for these patients [3, 57].

Hence, our data strongly suggest that the interaction between CEACAM1‐TIM3 among tumor cells and CTLs could represent an unrecognized immunosuppressive mechanism mediated by tamoxifen. This interaction likely suppresses anti‐tumor immunity, negating the lymphocyte‐attractive effects of ISGs and eventually leading to worse patient outcomes.

### 2.2 Tamoxifen‐Induced Activation of the cGAS/STING and JAK/STAT Pathways, Along with the Loss of RACK7/KDM5C, is Indispensable for ISG Induction

**FIGURE 3:**
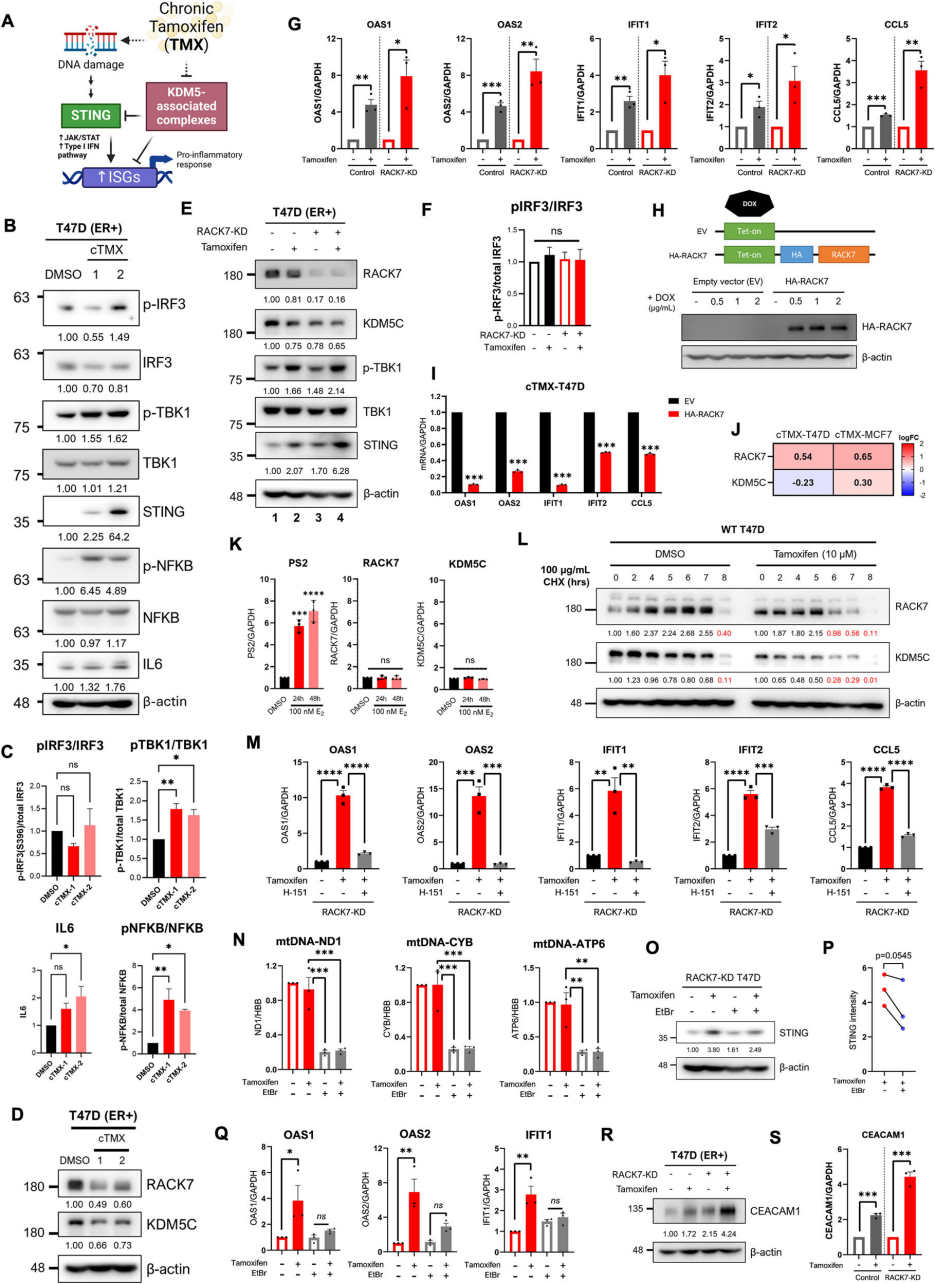
Tamoxifen‐induced type I interferon signaling is mediated by activation of the cGAS/STING pathway and loss of the histone H3K4 demethylase complex components RACK7 and KDM5C. **A**. Schematic of the proposed tamoxifen‐mediated regulation of type I IFN signaling and ISGs. **B, C,** and **D**. Representative immunoblots (B) and quantification (C) of alterations in protein expression and/or phosphorylation status of components in the cGAS/STING pathway, NF‐κB, IL6, and the histone H3K4 demethylase complex components (D) following chronic tamoxifen (cTMX) treatment. Immunoblotting shows the expression of the indicated total or phosphorylated proteins in control and two independent cTMX‐T47D clones. The levels of β‐actin serve as loading control. **E** and **F**. Representative immunoblots (E) of protein expression and/or phosphorylation status of RACK7/KDM5C and cGAS/STING components; and p‐IRF3 quantification (F) in T47D cells, with or without RACK7 knockdown, treated with 5 µM of tamoxifen or vehicle control (DMSO) for 48 hours, assessed by immunoblotting. The levels of β‐actin serve as loading control. **G**. RT‐qPCR analysis showing relative mRNA expression levels of selected ISGs in control T47D (gray) or RACK7‐KD T47D (red) cells treated with tamoxifen (10 µM) for 48 hours (solid bars) compared to DMSO‐treated controls (hollow bars). Data is presented as mean log2FC ± SEM, *n* = 3, *p*‐values are calculated using *t*‐test per group, *p < 0.05, **p < 0.005. **H**. Schematic diagram of the doxycycline (DOX)‐induced HA‐RACK7 overexpression system (upper panel). Immunoblotting showing HA‐RACK7 overexpression induced by different concentrations of DOX (lower panel). **I**. RT‐qPCR analysis showing relative mRNA expression levels of selected ISGs in the empty vector (EV) and HA‐RACK7 overexpressing cTMX‐T47D cells. Data is presented as mean log2FC ± SEM, *n* = 3, *p*‐values are calculated using multiple *t*‐tests followed by Holm‐Sidak correction, ***p < 0.001. **J**. Heatmap plot indicating the logFC in mRNA expression of RACK7 and KDM5C between cTMX‐T47D and cTMX‐MCF7 cells compared to control (DMSO‐treated) cells. Data are summarized from the RNA‐seq analysis of cTMX cells. **K**. RT‐qPCR analysis showing relative mRNA expression levels of PS2 (ER target gene), RACK7, and KDM5C in T47D cells treated with estradiol (E_2_, 100 nM) for 24 and 48 hours compared to control (DMSO‐treated) cells. Data is presented as mean log2FC ± SEM, *n* = 3, *p*‐values are calculated using one‐way ANOVA followed by Tukey post‐hoc test, ***p < 0.0005, ****p < 0.0001. **L**. Detection of protein levels of RACK7 and KDM5C in parental T47D cells treated with 10 µM of tamoxifen or vehicle control (DMSO) for 48 hours, followed by 100 ug/mL cycloheximide (CHX) for 0, 2, 4, 5, 6, 7, and 8 hours, assessed by immunoblotting. The levels of β‐actin serve as loading control. **M**. RT‐qPCR analysis showing the relative mRNA expression levels of selected ISGs in RACK7‐KD T47D cells treated with tamoxifen (10 µM) or a combination of tamoxifen and the STING antagonist H‐151 (2 µM) for 48 hours, compared to control (DMSO‐treated) cells. Data is presented as mean log2FC ± SEM, *n* = 3, *p*‐values are calculated using one‐way ANOVA followed by Tukey post‐hoc test, **p < 0.005, ***p < 0.0005, ****p < 0.0001. **N**. RT‐qPCR analysis showing the relative genomic copy number of mtDNA‐encoded genes (as indicated) in RACK7‐KD T47D cells treated with tamoxifen (10 µM), EtBr (50 ng/mL), or a combination of tamoxifen and EtBr, compared to control (DMSO‐treated) cells. The levels of the nuclear gene HBB serve as normalization control. Data is presented as mean log2FC ± SEM, *n* = 3, *p*‐values are calculated using one‐way ANOVA followed by Tukey post‐hoc test, **p < 0.005, ***p < 0.0005. **O** and **P**. Representative immunoblotting (O) and quantification (P) showing the protein levels of STING in RACK7‐KD T47D cells treated under the same conditions as described in (N). Data is presented as paired quantifications per independent replicate, *n* = 3, *p*‐value is calculated using paired *t*‐test. **Q**. RT‐qPCR analysis showing the relative mRNA expression levels of selected ISGs in RACK7‐KD T47D cells treated with tamoxifen, EtBr, or a combination of tamoxifen and EtBr, compared to control (DMSO‐treated) cells. Data is presented as mean log2FC ± SEM, *n* = 3, *p*‐values are calculated using one‐way ANOVA followed by Tukey post‐hoc test, *p < 0.05, **p < 0.005. **R** and **S**. Detection of protein (R) and mRNA (S) expression levels of CEACAM1 in T47D cells, with or without RACK7 knockdown, treated with 5 µM of tamoxifen or vehicle control (DMSO) for 48 hours, assessed by immunoblotting and RT‐qPCR, respectively. RT‐qPCR data is presented as mean log2FC ± SEM, *n* = 3, *p*‐values are calculated using *t*‐test per group, ***p < 0.0005. Numbers below the panels of the immunoblots indicate the densitometric values normalized to the corresponding control lane. p, phosphorylation; cTMX, chronic tamoxifen; KD, knockdown; mtDNA, mitochondrial DNA; EtBr, ethidium bromide; logFC, log fold change.

The regulation of type I IFNs and ISGs largely depends on the activation of the cGAS/STING and JAK/STAT signaling pathways. These signaling pathways are primarily regulated by viral genomes or DNA‐damage induced, phosphorylation‐dependent activation mechanisms. Key components involved in this axis include cGAS/STING‐associated factors such as STING, TBK1, and IRF3, as well as JAK/STAT‐associated factors such as STAT1 and STAT3 [34, 35]. Additionally, the KDM5 family of H3K4 demethylases, such as KDM5C, function as transcription corepressors for STING (TMEM173) gene regulation [43]. They often form a complex with the non‐enzymatic scaffold protein RACK7, which also functions as a DNA damage repair factor [58], and have been shown to regulate the cGAS/STING pathway [42] (Figure 3A). To investigate whether prolonged tamoxifen treatment influences the cGAS/STING and JAK/STAT pathways, as well as the KDM5C‐RACK7 corepressor complex, we monitored the protein expression levels and phosphorylation status of STAT1, STAT3, IRF3, and TBK1, along with the protein levels of STING, KDM5C, and RACK7 in T47D and MCF7 cells during long‐term (up to 6 months) tamoxifen treatment. Continuous tamoxifen treatment consistently triggered phosphorylation of STAT1 and/or STAT3 in both ER^+^ cell lines (Supplementary Figure S5A and S5B). Additionally, marked phosphorylation of TBK1 was observed in cTMX‐T47D cells, whereas no significant changes in IRF3 phosphorylation were detected across these ER^+^ cell lines (Figure 3B and C, Supplementary Figure 5C). Notably, increased STING protein levels were consistently observed in both T47D and MCF7 cells following extended tamoxifen treatment (Figure 3B, Supplementary Figure S5C), accompanied by a gradual decrease in KDM5C and RACK7 protein levels (Figure 3D, Supplementary Figure S5A and S5B). Given that STING serves as a scaffold to coordinate downstream TBK1–IRF3 and IKK–NF‐κB pathways [34], we next examined whether chronic tamoxifen exposure preferentially engage NF‐κB signaling. Indeed, both NF‐kB phosphorylation and the expression of its downstream target IL‐6 showed a clear trend toward, or significant, upregulation under long‐term tamoxifen treatment (Figure 3B and C). Collectively, these data indicate that tamoxifen induces ISG expression through activating the cGAS/STING and JAK/STAT pathways, primarily via the STING–NF‐kB–IL6 axis (see details in Discussion).

Given that KDM5 demethylases have been shown to repress *STING* gene expression [43], we hypothesize that the depletion of KDM5C and RACK7 may be a key factor for tamoxifen‐induced STING upregulation. Since RACK7 plays a critical role as a chromatin reader within the KDM5 demethylase complex, facilitating the recruitment of KDM5 chromatin modifiers to enhancers and/or promoters for target gene repression [40, 41], we tested whether RACK7 is an essential factor for the tamoxifen‐induced upregulation of STING and ISGs. To this end, we conducted short hairpin RNA (shRNA)‐mediated knockdown of RACK7 in T47D cells and then compared the induction of cGAS/STING signaling components and ISG expression between control and RACK7‐knockdown (RACK7‐KD) cells following short‐term tamoxifen treatment (48 hours). Short‐term tamoxifen exposure decreased both RACK7 and KDM5C levels while concurrently increasing STING levels, along with elevated levels of hyperphosphorylated TBK1 in control T47D cells (Figure 3E, lanes 1 and 2). Remarkably, loss of RACK7 significantly intensified tamoxifen‐induced STING upregulation and TBK1 hyperphosphorylation, with minimal effect on IRF3 phosphorylation in T47D cells (Figure 3E, lanes 2 and 4, Figure 3F). The impact of tamoxifen treatment in upregulating ISGs was also more pronounced in the RACK7‐KD cells compared to control cells (Figure 3G). Additionally, ectopic expression of HA‐tagged RACK7 (HA‐RACK7) in cTMX‐T47D cells resulted in significant suppression of ISG expression, providing further confirmation of RACK7's involvement in tamoxifen‐induced upregulation of ISGs (Figure 3H and 3I). Interestingly, mRNA expression of both RACK7 and KDM5C showed no significant changes after prolonged tamoxifen treatment (Figure 3J), despite significant reductions in protein levels (Figure 3D). mRNA expression of both RACK7 and KDM5C was also unaffected by treatment of T47D cells with estradiol (E_2_), the natural ER ligand (Figure 3K). These findings strongly suggest that the tamoxifen‐induced depletion of RACK7 and KDM5C occurs through post‐translational regulatory mechanisms (i.e., protein degradation, complex stability) rather than through tamoxifen‐mediated inhibition of the ER‐signaling transcriptional pathway. To test this hypothesis, we employed the protein synthesis inhibitor cycloheximide (CHX) to monitor the turnover rates of both RACK7 and KDM5C following 48 hours of pre‐treatment with tamoxifen or the vehicle control DMSO. In control (DMSO‐treated) cells, both RACK7 and KDM5C proteins remained relatively stable up to 7 hours after CHX treatment, with marked degradation observed at 8 hours. In contrast, tamoxifen pre‐treatment accelerated protein turnover, with degradation initiating as early as 6 hours and resulting in near‐complete loss of both RACK7 and KDM5C by 8 hours post‐CHX treatment (Figure 3L). These data strongly indicate that tamoxifen depletes RACK7 and KDM5C by negatively impacting their protein stability, leading to enhanced activation of both STING and ISGs during chronic tamoxifen exposure.

To evaluate STING pathway activation under more physiologically relevant conditions, we employed a T47D xenograft model in nude mice, which lack functional T cells and therefore enable assessment of tumor‐intrinsic and non‐T‐cell‐mediated effects. Control or RACK7‐KD T47D cells were implanted and treated with vehicle or tamoxifen for up to three weeks. Although T47D xenograft tumors exhibited limited growth (data not shown), probably due to non‐T‐cell‐mediated anti‐tumor effects, we were able to obtain sufficient tumor material for CyTOF analysis. Owing to the relatively small tumor size and limited immune cell infiltration, analyses were restricted to tumor and stromal cell populations (Supplementary Figure S6A). Combined tamoxifen treatment and RACK7 loss significantly increased total STING levels in tumor cells, consistent with our observations in cTMX‐T47D cells (Figure 3B), indicating that chronic tamoxifen exposure together with RACK7 downregulation induce tumor cell‐intrinsic STING accumulation. Notably, upon the same conditions, we also detected robust phosphorylation of STING and IRF3 in tumor cells (Supplementary Figure S6B), which was not observed in cTMX‐T47D cells in vitro (Figure 3B). These findings suggest that canonical STING pathway activation – characterized by STING and IRF3 phosphorylation – may be facilitated in vivo through non‐cell autonomous mechanisms involving interactions within the tumor microenvironment (see details in Discussion). In contrast, stromal cells exhibited detectable STING pathway activation under untreated condition, which was reduced upon tamoxifen treatment alone or in combination with RACK7 knockdown (Supplementary Figure S5C). This observation indicates that the tamoxifen‐mediated STING signaling activation is largely restricted to tumor cells and may not extend to stromal compartments.

Among the different cGAS/STING factors evaluated, the upregulation of STING consistently paralleled the increase in ISG induction upon combined tamoxifen treatment and RACK7‐knockdown (Figure 3E), suggesting its critical role in the tamoxifen/RACK7‐KD‐mediated activation of ISGs. To illustrate the crucial role of STING in this regulation, we employed the selective STING antagonist H‐151, which inhibits activation of both IRF3 and NF‐κB, thereby reducing type I IFN and inflammatory cytokine production [59]. As expected, the impact of short‐term tamoxifen treatment and RACK7 knockdown on ISG induction was significantly reduced with STING inhibition (Figure 3M). We further demonstrated that ethidium bromide (EtBr)‐depletion of mtDNA (Figure 3N) hampers the tamoxifen and RACK7 loss‐mediated increase in STING cellular levels (Figure 3O and P), and more critically, diminishes or completely abolishes ISG induction (Figure 3Q). Consistent with our cell‐based findings, an inverse correlation was observed between RACK7 and STING expression in patients with relapsed ER^+^ breast cancer (Supplementary Figure S5D). Furthermore, a positive correlation was identified between STING expression and ISG levels. Conversely, both RACK7 and KDM5C expression showed an inverse relationship with ISG levels in these patients (Supplementary Figure S5D). Beyond upregulating ISGs, our data reveal that the tamoxifen‐RACK7 regulatory axis also upregulates the TIM3‐inhibitory ligand CEACAM1 at both mRNA and protein levels (Figure 3R and S).

Taken together, our findings indicate a clear mechanism for tamoxifen‐mediated immunomodulation. Tamoxifen treatment induces mtDNA damage, activating the cGAS/STING, JAK/STAT, and IFN‐I signaling pathways, which leads to the upregulation of ISGs. Concurrently, RACK7/KDM5C depletion by tamoxifen further enhances this effect by derepressing *STING*, thereby ensuring robust engagement of the cGAS/STING pathway. Furthermore, our findings suggest that tamoxifen simultaneously upregulates CEACAM1 transcriptional activation by suppressing the protein levels of the RACK7/KDM5C corepressor complex, leading to activation of the CEACAM1‐TIM3 immune checkpoint pathway and resulting in T‐cell exhaustion.

### 2.3 The Combined Treatment of Tamoxifen and RACK7‐Knockdown Promotes the Growth of ER^+^ Tumor via Modulating an Immunosuppressive Tumor Microenvironment

**FIGURE 4:**
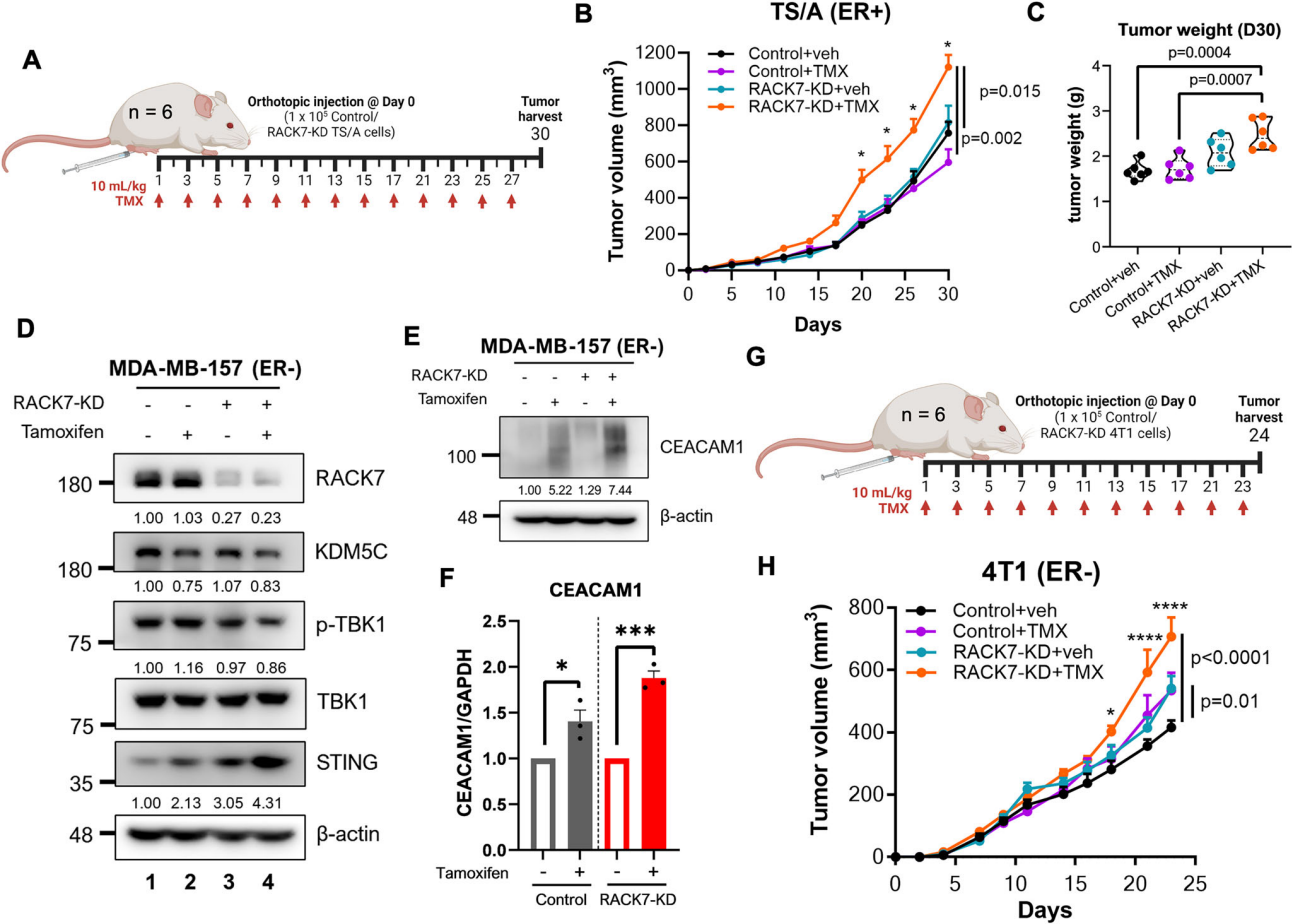
Combination treatment of tamoxifen and RACK7 knockdown shapes a T‐cell exhaustive microenvironment that promotes growth of ER‐positive and ER‐negative breast tumors in vivo. **A**. Schematic of experimental design for TS/A syngeneic tumor mouse experiments. **B** and **C**. Growth curves (B) and tumor weight (C) of TS/A ER‐positive (ER^+^) breast cancer cells transduced with scramble control‐shRNA (Control) or shRACK7 (RACK7‐KD) in syngeneic BALB/c mice (*n* = 6) under continuous tamoxifen administration for 30 days. Tumor growth is presented as mean tumor volume + SEM, *n* = 6, statistical significance was calculated by mixed‐effects modeling with Geisser‐Greenhouse correction, *p < 0.05. Tumor weight is presented as violin plots of tumor weight, *n* = 6, *p*‐values are calculated using one‐way ANOVA followed by Tukey post‐hoc test. **D**. Detection of protein expression and/or phosphorylation status of RACK7/KDM5C and cGAS/STING components in ER‐negative (ER^−^) MDA‐MB‐157 breast cancer cells, with or without RACK7 knockdown, treated with 5 µM of tamoxifen or vehicle control (DMSO) for 48 hours, assessed by immunoblotting. The levels of β‐actin serve as loading control. **E** and **F**. Detection of protein (E) and mRNA (F) expression levels of CEACAM1 in MDA‐MB‐157 cells, with or without RACK7 knockdown, treated with 5µM of tamoxifen or vehicle control (DMSO) for 48 hours, assessed by immunoblotting and RT‐qPCR, respectively. RT‐qPCR data is presented as mean log2FC ± SEM, *n* = 3, *p*‐values are calculated using *t*‐test per group, *p < 0.05, ***p < 0.0005. β‐actin serve as loading control. Numbers below the panels of the immunoblots indicate the densitometric values normalized to the corresponding control lane. **G**. Schematic of experimental design for 4T1 syngeneic tumor mouse experiments. **H**. Growth curves of 4T1 (ER^−^) breast cancer cells transduced with scramble control‐shRNA (Control) or shRACK7 (RACK7‐KD) in syngeneic BALB/c mice (*n* = 6) under continuous tamoxifen administration for 24 days. Tumor growth is presented as mean tumor volume + SEM, *n* = 6, statistical significance was calculated by mixed‐effects modeling with Geisser‐Greenhouse correction, *p < 0.05, ****p < 0.0001. Veh, vehicle; TMX, tamoxifen; KD, knockdown.

Although tamoxifen‐induced ISGs promote CTL infiltration into the tumor microenvironment, the concomitant immunosuppressive effects – such as IL6 upregulation and CEACAM1 induction – suggest that many of these infiltrating CTLs may become functionally exhausted and unable to effectively eliminate tumor cells. Based on our in vitro findings that robust CEACAM1 induction occurs when RACK7 is lost under long‐term tamoxifen exposure, we considered two clinically relevant scenarios happening at tumor recurrence: (1) tumors retaining basal RACK7 expression and (2) tumors with reduced RACK7 levels due to prolonged tamoxifen exposure, in which continued tamoxifen treatment may further amplify CEACAM1 upregulation. To directly investigate the effect of the tamoxifen–RACK7–CEACAM1 regulatory axis in vivo, we established syngeneic tumor mouse models by orthotopically transplanting either control or RACK7‐knockdown mouse TS/A (ER^+^) breast cancer cells into the mammary fat pad of female BALB/c mice. To simulate tumor recurrence following adjuvant endocrine therapy after primary surgery, we initiated tamoxifen treatment on day 1 after tumor implantation and closely monitored the tumor growth during the experimental period (Figure 4A). While tamoxifen treatment or RACK7‐knockdown alone had modest or nonsignificant effects on tumor growth in the TS/A model, their combination markedly promoted tumor progression (Figure 4B and C). The lack of tumor‐promoting effects with either condition alone likely reflects opposing influences of tamoxifen – direct inhibition of tumor cell proliferation versus modulation of the immune microenvironment – as well as the minimal impact of RACK7 loss on intrinsic tumor growth. These findings suggest that tamoxifen–RACK7–CEACAM1 axis drive tumor progression through both tumor cell‐intrinsic mechanisms and the establishment of an immunosuppressive tumor microenvironment. To further elucidate the mechanisms driving the enhanced pro‐tumor effects of tamoxifen and RACK7‐knockdown, we examined the tumor immune microenvironment in TS/A tumors by flow cytometry. Our initial flow cytometry analysis of tumor‐infiltrating immune populations revealed that, beyond T cells, additional immune compartments were variably affected by tamoxifen treatment and/or RACK7 knockdown (Supplementary Figure S7 and S8). In the TS/A model, although changes were observed in specific conditions – such as alterations in total myeloid cell populations and certain myeloid subsets – these effects were not consistently statistically significant when comparing the combined tamoxifen and RACK7 knockdown condition with controls. Given this variability, we focused on our analysis on tumor cells expressing CEACAM1 or PD‐L1, along with the corresponding CD4^+^/CD8^+^ T‐cell populations. We found that prolonged tamoxifen treatment induces CEACAM1 (CD66a) presentation in the CD45‐negative (CD45^−^) tumor cell population (Supplementary Figure S8E). Induction of PD‐L1 (CD274) was also observed in the CD45^−^ tumor cell population, albeit at a lower rate (Supplementary Figure S8F). We further assessed the immune cell composition within the tumor microenvironment. We observed that there is a tendency towards increased infiltration of CD45‐positive (CD45^+^) immune cells in the group exposed to the combined treatment of tamoxifen and RACK7‐knockdown (Supplementary Figure S7C). While the CD3^+^ T‐cell population remained consistent across all groups (Supplementary Figure S7D), a significant elevation in TIM3^+^ CD8 T‐cells was observed in the tamoxifen combined with RACK7‐knockdown group (Supplementary Figure S8C). This is consistent with the enhanced tumor growth observed in the TS/A syngeneic tumor mouse model (Figure 4B and C). Notably, the population of PD1^+^ CD8 T‐cells, as well as TIM3^+^ or PD1^+^ CD4 T‐cells in the tumor microenvironment, remained unaffected (Supplementary Figure S8A, S8B, and S8D). Collectively, these findings suggest that prolonged tamoxifen treatment modulates an immunosuppressive tumor microenvironment by inducing the CEACAM1‐TIM3, STING‐associated, as well as PD‐L1‐PD1 pathways. The immunomodulatory effects of tamoxifen are further amplified when RACK7 expression is substantially reduced, although some discrepancies between in vitro and in vivo models likely reflect context‐dependent regulation (see Discussion). Together, these changes promote a tumor microenvironment that favors immune evasion and supports tumor progression.

### 2.4 The Tamoxifen–RACK7–CEACAM1 Axis also Contributes to Enhancing Tumor Growth in ER^−^ Tumors

Loss of ER expression and reduced dependence on estrogen signaling are major resistance mechanisms among ER^+^ patients [17]. Notably, approximately 30% of patients convert from ER^+^ to ER‐negative (ER^−^) status following extended adjuvant tamoxifen treatment [60]. To investigate whether the pro‐tumor effects associated with the tamoxifen–RACK7–CEACAM1 axis could also be identified in ER^−^ breast tumors despite the loss or reduction of ER, we selected ER^−^ cell models, including human MDA‐MB‐157 and mouse 4T1 TNBC cell lines. Similar to ER^+^ cell lines (Figure 3E), we observed that the combined treatment of tamoxifen and RACK7‐knockdown substantially triggers STING accumulation in MDA‐MB‐157 cells, whereas phosphorylation of TBK1 and IRF3 remained unchanged or undetectable (Figure 4D). These findings suggest that tamoxifen may induce a noncanonical mode of STING regulation distinct from the classical activation‐degradation dynamics (see details in Discussion). Additionally, this combination treatment upregulated the TIM3‐inhibitory ligand CEACAM1 at both mRNA and protein levels (Figure 4E and F). These findings indicate that the mechanism of tamoxifen and RACK7 loss affecting cellular STING and CEACAM1 levels may also apply to ER^−^ cancer cells. To investigate the immunomodulatory effects of tamoxifen in combination with RACK7 loss in ER^−^ tumors, we orthotopically implanted control or RACK7‐knockdown 4T1 cells into immunocompetent BALB/c mice following extended tamoxifen treatment. Consistent with the results observed in ER^+^ TS/A tumors, neither tamoxifen nor RACK7 knockdown alone had a substantial effect on promoting tumor growth in ER^−^ 4T1 tumors (Figure 4G and H). However, the tumor‐promoting effects became markedly more pronounced when tamoxifen treatment was combined with RACK7 knockdown (Figure 4H). Furthermore, immunohistochemical (IHC) analysis of FFPE tumor tissues from the 4T1 syngeneic model revealed increased expression of both CEACAM1 and STING in the combined treatment group compared to controls (Supplementary Figure S9A and S9B). Our mass cytometry (CyTOF) profiling further revealed alterations in the immune landscape following single or combined treatment of tamoxifen and RACK7‐knockdown (Supplementary Figure S10A–M). We observed lower amounts of natural killer (NK) cells and higher amounts of CD64^+^ macrophages in the tamoxifen combined with RACK7‐knockdown group compared to tamoxifen treatment alone or RACK7‐knockdown alone (Supplementary Figure S10C and S10D). Further analysis of the macrophage population revealed an enrichment of CD64^+^ macrophages (metaclusters 9, 11, 12 in Supplementary Figure S11B–E), which exhibited elevated expression of PD‐L1 (CD274), suggesting that this combination treatment may also promote the expansion or activation of tumor‐associated macrophages [61]. However, no significant changes were found in the CD4^+^ and CD8^+^ T‐cell populations (Supplementary Figure S10J and S10K). Interestingly, closer examination of the CD8^+^ T‐cells revealed a highly heterogeneous population that could be further classified into naïve, progenitor‐exhausted, and terminally‐exhausted CD8^+^ T‐cells based on the differential expression of several T‐cell specific exhaustion markers (Supplementary Figure S10N–P and S10Q–Z). Notably, a distinct shift toward a terminally‐exhausted cellular phenotype in CD8^+^ T‐cells was evident in the tamoxifen combined with RACK7‐knockdown group (Supplementary Figure S10N–P). Further analysis of PD1 and TIM3 expression within the CD8^+^ T‐cells population showed that RACK7 knockdown alone (restricted to tumor cells) did not significantly alter either PD1^+^ or TIM3^+^ CD8^+^ T‐cell populations. Similarly, tamoxifen treatment alone predominantly increased PD1^+^ cells without inducing TIM3^+^ or PD1^+^/TIM3^+^ populations, suggesting that the threshold for recruiting TIM3^+^ T cells is higher in 4T1 tumors than in TS/A tumors. Importantly, the addition of RACK7 knockdown appears to lower this threshold, facilitating the accumulation of terminally exhausted (PD1^+^/TIM3^+^) CD8^+^ T cells (Supplementary Figure S10P). These findings indicate that TIM3 induction requires both tamoxifen‐driven signaling and sufficient reduction of RACK7, supporting a combinatorial, threshold‐dependent mechanism rather than a direct effect of tamoxifen alone. This transition likely drives a highly T‐cell‐exhaustive microenvironment within the ER^−^ 4T1 tumor, and may account for the pro‐tumor effects observed within this distinct immunomodulatory context. Overall, our findings strongly suggest that the tamoxifen–RACK7–CEACAM1 axis may persist not only in ER^+^ tumors but also endure after relapse‐associated ER conversion, indicating that similar immunosuppressive mechanisms may govern the pro‐tumor effects in both ER^+^ and ER^−^ tumors.

### 2.5 Blocking the Interaction Between TIM3 and CEACAM1 Reshapes the Tamoxifen‐Modulated Immunosuppressive Tumor Microenvironment, Leading to Effective Inhibition of Both ER^+^ and ER^−^ Tumor Growth

**FIGURE 5:**
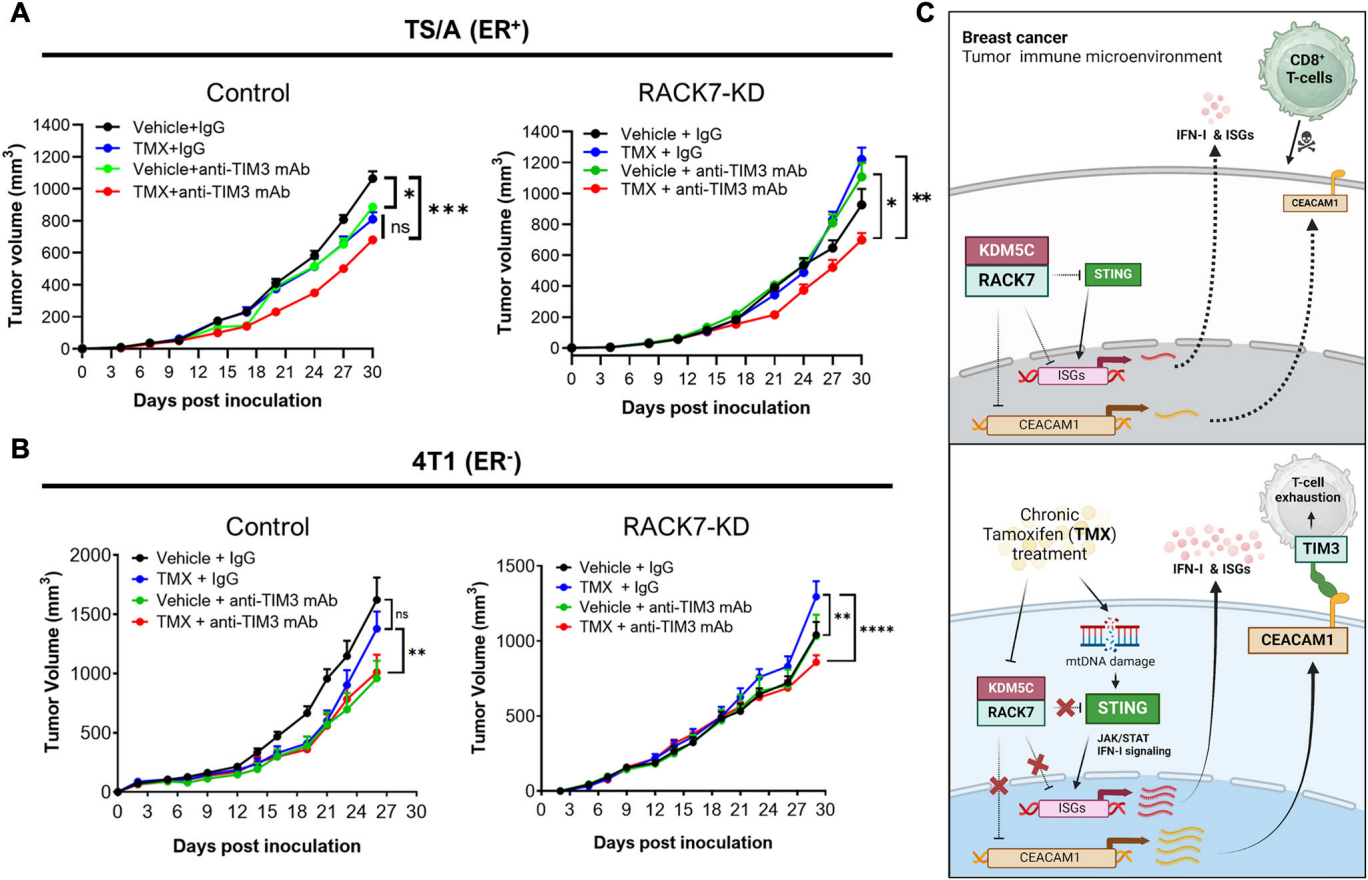
Anti‐TIM3 antibody‐based immune checkpoint blockade mitigates tamoxifen and RACK7 loss‐induced tumor progression in both ER‐positive and ER‐negative breast cancer. **A** and **B**. Growth curves of Control (left panels) and RACK7‐KD (right panels) TS/A (A) or 4T1 (B) breast cancer cells in syngeneic BALB/c mice (*n* = 8) under continuous TMX or vehicle (corn oil) administration for up to 30 days. Immune checkpoint blockade (ICB) using anti‐TIM3 antibodies were also tested in various combinations with TMX or vehicle as indicated. The corresponding isotype antibody was used as control. Tumor growth is presented as mean tumor volume + SEM, *n* = 8, statistical significance was calculated by mixed‐effects modeling with Geisser‐Greenhouse correction, *p < 0.05, **p < 0.005, ***p < 0.0005 ****p < 0.0001. **C**. A model illustrating how long‐term tamoxifen treatment modulates an immunosuppressive tumor microenvironment, promoting pro‐tumor immunity and progressive tumor growth. Tamoxifen mediates breast tumor immunosuppression through multiple cellular regulators and pathways. Chronic tamoxifen treatment induces mitochondrial DNA (mtDNA) damage. This activates the cGAS/STING, JAK/STAT, and type I IFN (IFN‐I) signaling pathways, leading to enhanced expression of interferon‐stimulated genes (ISGs). Additionally, tamoxifen depletes the RACK7/KDM5C complex, a known transcription corepressor of STING and ISGs. The net effect of tamoxifen‐induced cGAS/STING pathway activation and loss of the RACK7/KDM5C complex creates an inflammatory and lymphocyte‐attracting microenvironment. However, loss of the RACK7/KDM5C complex also upregulates CEACAM1, which can exhaust T‐cells through interaction with TIM3. In breast tumors, the combined effects of tamoxifen are associated with higher presentation of CEACAM1 on tumor cells, an increased population of TIM3‐positive (TIM3^+^) CD8 T‐cells in the tumor microenvironment (TME), and enhanced tumor progression. The picture is generated using Biorender.com.

The observed increase in tumor growth and immunosuppressive modulation in both ER^+^ TS/A (Figure 4B) and ER^−^ 4T1 (Figure 4H) syngeneic tumor mouse models strongly support the role of the CEACAM1–TIM3 axis in tamoxifen‐induced pro‐tumor immunity. Hence, we hypothesize that blocking the CEACAM1‐TIM3 interaction could result in tumor regression in these preclinical mouse models. To test this hypothesis, we administered a neutralizing anti‐TIM3 monoclonal antibody (TIM3‐mAb) alongside tamoxifen in TS/A and 4T1 syngeneic tumor mouse models carrying tumor cell‐specific RACK7 knockdown, thereby mimicking the chronic tamoxifen‐treatment condition identified in this study (Figure 5A and B). In TS/A control tumors, tamoxifen alone significantly delayed tumor growth, reflecting its canonical ER‐dependent anti‐proliferation effect (Figure 5A, left panel). However, immune profiling revealed concurrent increases in PD1^+^, TIM3^+^, and PD1^+^/TIM3^+^ CD4^+^ and CD8^+^ T‐cell populations (Supplementary Figure S12A), indicating induction of T‐cell exhaustion. This dual effect likely underlies the limited efficacy of tamoxifen monotherapy. In contrast, addition of TIM3‐mAb reversed this exhausted phenotype, markedly reducing PD1^+^, TIM3^+^, and PD1^+^/TIM3^+^ T‐cell frequencies and producing the strongest anti‐tumor response.

In 4T1 control tumors, tamoxifen alone had minimal impact on tumor growth, consistent with the absence of ER signaling (Figure 5B, left panel). In contrast, TIM3‐mAb monotherapy exhibited clear anti‐tumor activity comparable to the tamoxifen plus TIM3‐mAb combination. Immune profiling showed broadly similar T‐cell frequencies across treatment groups (Supplementary Figure S13A), suggesting that TIM3 blockade may exert anti‐tumor effects that are, at least in part, independent of tamoxifen in ER^−^ tumors. Further analysis revealed a significant increase in CD64^+^ macrophages following TIM3‐mAb treatment (Supplementary Figure S13I). Clustering analysis identified enrichment of macrophage subsets with M1‐like characteristics, including elevated CD80, CD86, and iNOS expression, which also exhibited high TIM3 levels (Supplementary Figure S14C–E). These findings suggest that TIM3 blockade may additionally enhance macrophage‐mediated anti‐tumor immunity, consistent with previous reports [62, 63]

In both TS/A and 4T1 RACK7‐KD tumors (Figure 5A and B, right panels), tamoxifen treatment accelerated tumor growth in both ER^+^ and ER^−^ context, consistent with our in vitro findings that RACK7 loss enhances tamoxifen‐mediated immunosuppressive effects. Although the changes did not reach statistical significance, immune profiling revealed a consistent trend toward increased PD1^+^, TIM3^+^, and PD1^+^/TIM3^+^ CD8^+^ T‐cell populations in TS/A RACK7‐KD tumors following tamoxifen treatment (Supplementary Figure S12C). A similar trend was observed in 4T1 RACK7‐KD tumors, with increased PD1^+^ and PD1^+^/TIM3^+^ CD8^+^ T‐cell populations upon TMX treatment (Supplementary Figure S14B). Notably, TIM3‐mAb treatment did not significantly reduce the frequencies of TIM3^+^ or PD1^+^/TIM3^+^ CD8^+^ T‐cells, yet still produced a clear anti‐tumor effect in tumor growth assays. This observation is consistent with clinical reports showing that TIM3 blockade can exert anti‐tumor efficacy without necessarily depleting TIM3‐expressing T‐cell populations [64].

To further evaluate the therapeutic potential of combining tamoxifen with TIM3‐mAb in ER^+^ breast cancer, we performed a post hoc surrogate survival analysis in TS/A control and RACK7‐KD tumors. The surrogate survival results were fully consistent with the tumor growth curves. In the TS/A control tumors (Supplementary Figure S15A and S15B), surrogate survival analysis revealed significant differences among treatment groups (log‐rank *p* < 0.05). Both tamoxifen and TIM3‐mAb monotherapies significantly delayed progression relative to control (*p* < 0.05), whereas the combination treatment produced the greatest delay and significantly outperformed both monotherapies (*p* < 0.05). In TS/A RACK7‐knockdown tumors (Supplementary Figure S15C and S15D), neither monotherapy significantly prolonged surrogate survival compared with control. In contrast, the combination treatment significantly extended surrogate survival relative to all other treatment groups (*p* < 0.05). Taken together, these findings suggest that TIM3 blockade may enhance the therapeutic efficacy of tamoxifen under both RACK7‐proficient and RACK7‐deficient conditions.

**FIGURE 6:**
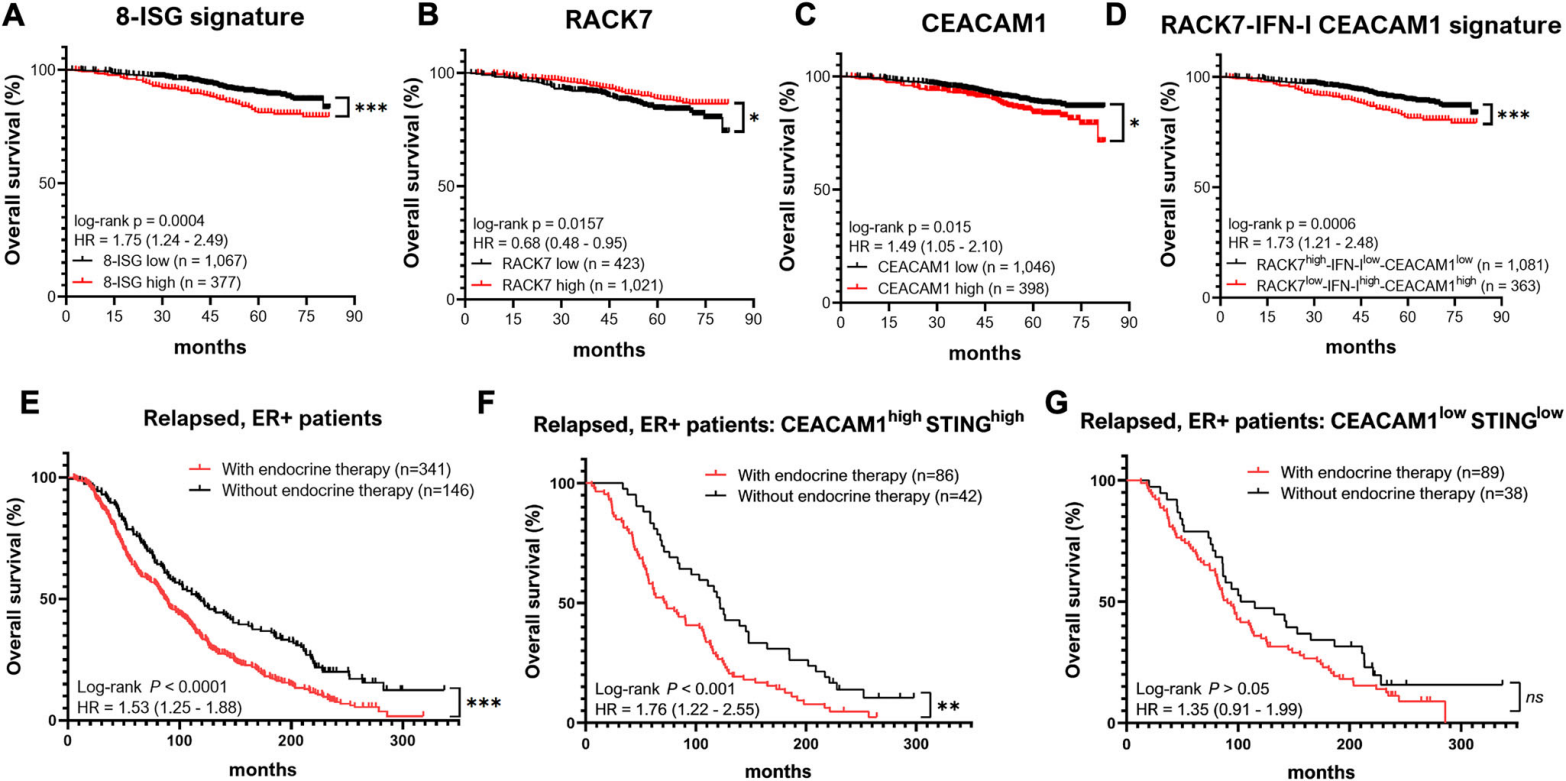
Continuous endocrine therapy of relapsed, ER^+^ patients with high CEACAM1 and high STING gene expression is correlated with poorer patient survival. **A**–**D**. Kaplan–Meier survival curves revealed the prognostic effect of the 8‐ISG gene signature (A), RACK7 expression (B), CEACAM1 expression (C), and the RACK7‐IFN‐I‐CEACAM1 gene signature (D) on the overall survival (OS) of ER^+^ breast cancer patients who have undergone endocrine therapy. Statistical significance was calculated using the log‐rank (Mantel‐Cox) test, *p < 0.05, ***p < 0.0005. **E**–**G**. Kaplan–Meier survival curves revealed the prognostic effect of endocrine therapy on overall survival (OS) in relapsed, ER^+^ patients (E), patients exhibiting high levels of both CEACAM1 and STING (CEACAM1^high^/ STING^high^) (F), and patients exhibiting low levels of both CEACAM1 and STING (CEACAM1^low^/ STING^low^) (G). Statistical significance was calculated using the log‐rank (Mantel‐Cox) test, *p < 0.05, **p < 0.001, ***p < 0.0001. The genes included in the 8‐ISG signature as indicated in Figure 1H include OAS1, OAS2, OASL, IFIT1, IFIT2, IFIT3, RSAD2, and CCL5.

In summary, our findings indicate that blocking the CEACAM1‐TIM3 interaction could have therapeutic significance in governing prolonged tamoxifen‐treated breast cancer tumors, whether they remain ER^+^ or convert to ER^−^ during tumor recurrence. Furthermore, our data highlight the critical role of loss of the RACK7‐containing KDM5 histone demethylase complex in facilitating tamoxifen's ability to create an immunosuppressive tumor microenvironment, primarily by enhancing both STING and CEACAM1 expression following long‐term tamoxifen treatment (Figure 5C). In support of our findings, survival analysis of ER^+^ patients who received endocrine therapy showed a worse prognosis for those exhibiting a higher 8‐ISG signature (Figure 6A), lower RACK7 expression (Figure 6B), and higher CEACAM1 expression (Figure 6C). Importantly, the presence of this tamoxifen‐induced gene signature–characterized by low RACK7, high IFN‐I, and high CEACAM1 expression–was associated with significantly worse patient outcomes (Figure 6D).

## 3 Discussion

ICB therapy has revolutionized cancer treatment, including for poorly immunogenic breast cancer. Numerous studies indicate that elevated PD‐L1 expression in breast cancer correlates with better response and survival rates in patients treated with anti‐PD1/PD‐L1 monotherapy [65, 66, 67, 68]. In line with this, the FDA has authorized the combination therapy of anti‐PD1/PD‐L1 antibodies and chemotherapy for PD‐L1‐positive TNBC, showing a significant, yet moderate, enhancement in efficacy for tumors exhibiting high PD‐L1 expression [10]. Conversely, tumors lacking PD‐L1 expression have not demonstrated any therapeutic benefit from this combination of ICB and chemotherapy [6]. While responses to this anti‐PD1/PD‐L1 immune‐chemotherapy combination appear to be confined to TNBC patients due to limited immune modulation efficacy in ER^+^ breast cancer patients [9, 10, 11, 12], an ongoing phase III clinical study (NCT03725059) has demonstrated that a regimen of neoadjuvant chemotherapy with anti‐PD1 antibody pembrolizumab, followed by adjuvant endocrine therapy, significantly enhanced the rates of pathological complete response (pCR) among high‐risk, early‐stage, ER^+^/HER2^−^ breast cancer patients [69]. However, a significant number of patients still fail to respond to this anti‐PD1 combination therapy. These findings indicate that additional mechanisms of immune tolerance or resistance may counteract the effects of current anti‐PD1/PD‐L1 immunotherapy in breast cancer. Nevertheless, the potential confounding factors responsible for the unbeneficial immune responses remained undefined.

In this study, we show a novel mechanism of ICB resistance in ER^+^ breast cancers induced by prolonged tamoxifen treatment (Figure 5C). Mechanistically, we identify a dual function of tamoxifen in sustaining this ICB resistance mechanism: (1) activation of the STING–IFN‐I pathway and (2) upregulation of the T‐cell inhibitory ligand CEACAM1. Interestingly, we demonstrate that the RACK7‐containing KDM5 histone demethylase complex(es) plays a central role in mediating the unfavorable tamoxifen effects. Although activated IFN‐I signaling can create an inflammatory and lymphocyte‐attractive microenvironment, we found that infiltrating lymphocytes in the tumors, particularly T‐lymphocytes, likely experience exhaustion via the CEACAM1‐TIM3 pathway (Figure 4B and H). This highlights a tamoxifen‐driven preference for CEACAM1/TIM3‐mediated T‐cell exhaustion, providing insight into the suboptimal response of ER^+^ patients to the current ICB therapy, i.e., PD‐1/PD‐L1 blockade [44, 70]. Our findings further illustrate that blocking the CEACAM1‐TIM3 interaction can reverse tumor growth associated with this resistance mechanism. This underscores the potential therapeutic importance of blocking the CEACAM1‐TIM3 interaction in the management of tamoxifen‐treated recurrent tumors, irrespective of their ER^+^ or ER^−^ status.

Although tamoxifen is well‐characterized as an ER modulator, it also exhibits off‐target and non‐specific effects. For instance, tamoxifen induces oxidative cell stress, contributing to genotoxicity in both the nucleus and mitochondria [26, 71], and can also directly interact with DNA to form tamoxifen‐DNA adducts, which may activate the nucleic acid‐sensing cGAS/STING pathway after chronic treatment [72, 73]. Hence, we speculate that tamoxifen's DNA‐damaging potential may contribute to its resistance mechanism by modulating the tumor immune microenvironment, potentially extending to mtDNA – a major source of cytosolic dsDNA that activates the cGAS/STING‐IFN‐I pathway. Indeed, depleting mtDNA in tamoxifen‐treated T47D cells markedly diminished its impact on ISG activation (Figure 3N and Q). Our findings further elucidate the critical regulatory pathways involving tamoxifen–mtDNA–STING and tamoxifen–RACK7/KDM5C–STING in the activation of the cGAS/STING‐IFN‐I pathway via STING upregulation (Figure 5C). Functional inhibition of STING by the antagonist H‐151 dramatically abolished tamoxifen's effects on ISG expression (Figure 3M). The modulatory effects of tamoxifen on STING upregulation and ISG activation could be further enhanced by shRNA‐knockdown of RACK7 (Figure 3E and G), as inhibition of KDM5‐associated demethylase complexes lead to the derepression of STING and ISG expression [43], in line with our proposed model (Figure 5C). Therefore, our data indicate that tamoxifen has a dual function in activating the cGAS/STING‐IFN‐I pathway by promoting mtDNA damage and inhibiting post‐translational stabilization of the RACK7/KDM5C complex.

Surprisingly, while increased cellular STING levels appear to be a consistent feature of the tamoxifen–mtDNA–STING and tamoxifen–RACK7/KDM5C–STING axes across multiple breast cancer cell lines, downstream signaling events involving IRF3 phosphorylation display atypical patterns. Specifically, TBK1 phosphorylation was consistently detected in all cTMX‐T47D cell clones, whereas IRF3 phosphorylation was not significantly induced (Figure 3B). We therefore examined the effects of tamoxifen on NF‐κB activation, another downstream branch of STING signaling, in both DMSO control and cTMX‐T47D cells. Chronic tamoxifen exposure enhanced NF‐κB phosphorylation and increased IL6 expression (Figure 3C), supporting preferential engagement of an NF‐κB‐associated signaling program. Mechanistically, STING functions as a scaffold that coordinates signaling through both TBK1–IRF3 and IKK–NF‐κB pathways [34]. The lack of detectable IRF3 activation despite TBK1 phosphorylation suggests that STING signaling may be biased toward NF‐κB under these conditions. While one possibility is differential utilization of downstream signaling complexes, the precise mechanism remains unclear. In canonical STING signaling, activation is typically coupled to TBK1‐mediated phosphorylation events followed by STING degradation, which serves to limit signal duration and prevent excessive inflammation [74, 75]. In contrast, increased STING protein levels were consistently observed without evidence of robust IRF3 activation, suggesting that tamoxifen may alter STING regulatory dynamics. Altogether, these findings raise the possibility that tamoxifen induces a noncanonical mode of STING regulation characterized by STING accumulation and preferential engagement of NF‐κB‐associated signaling. However, given the absence of direct mechanistic evidence, this interpretation remains speculative and will require further investigation.

While cGAS‐STING and IFN‐I signaling pathways can promote both anti‐ and pro‐tumor immune responses [30, 36, 37], our findings strongly suggest that tamoxifen modulates an immunosuppressive microenvironment by upregulating *CEACAM1* expression. Our data showed that loss of RACK7 in tumor cells enhances tamoxifen‐mediated upregulation of CEACAM1 at both mRNA and protein levels (Figure 3R, S, 4E, and F), highlighting the significant role of RACK7's transcriptional repression in this process.

In our cell‐based and in vivo models, we combined tamoxifen treatment with RACK7 knockdown to approximate the long‐term effects of tamoxifen exposure. The combined condition – tamoxifen treatment together with tamoxifen‐induced reduction of RACK7 – leads to increased STING and CEACAM1 expression in both ER^+^ and ER^−^ breast cancer cells. However, while RACK7 depletion is tumor cell‐intrinsic in our system, tamoxifen exerts broader effects on the tumor microenvironment, influencing multiple immune cell populations as previously reported [22, 23]. These systemic effects likely contribute to discrepancies between in vitro and in vivo observations. For example, the lack of detectable increase in IRF3 and STING phosphorylation in T47D and cTMX‐T47D (Figure 3B and E) differs from the STING signaling observed in vivo (Supplementary Figure S6B). This discrepancy is likely attributable to the complexity of the tumor microenvironment, where STING pathway activation can be modulated by intercellular interactions. In particular, paracrine IFN‐I signaling from dendritic cells [76, 77, 78, 79], fibroblasts [80], and endothelial cells [81] may contribute to enhanced STING pathway activation in vivo. We also note that the synergistic upregulation of CEACAM1 observed in T47D cells was not fully recapitulated in TS/A tumors. Instead, tamoxifen treatment alone induced relatively high CEACAM1 expression in TS/A tumors (Supplementary Figure S8E vs. Figure 3R), suggesting context‐dependent regulation. Notably, increased CEACAM1 expression in TS/A tumors was associated with enhanced infiltration of TIM3^+^ CD8^+^ T cells (Supplementary Figure S8E vs. Supplementary Figure S8C), consistent with engagement of the CEACAM1–TIM3 signaling axis. Although RACK7 knockdown alone moderately increased STING and CEACAM1 expression and exerted limited effects on tumor growth in both in vitro and in vivo settings (Figure 3E, R, S, 4B, D, E, F, H), it did not significantly alter PD1, TIM3, or PD1^+^/TIM3^+^ T‐cell populations (Supplementary Figure S8A‐D, S10O–P). In contrast, tamoxifen treatment alone promotes a shift of naïve CD8^+^ T cells toward a progenitor‐exhausted phenotype, as evidenced by increased PD‐1 expression (Supplementary Figure S10O‐P). Strikingly, the combination of tamoxifen and RACK7‐knockdown – mimicking the chronic tamoxifen treatment condition identified in this study – resulted in increased T cell infiltration with a predominance of terminally exhausted (PD1^+^/TIM3^+^) CD8^+^ T cells (Supplementary Figure S8C and S10N). Together, these findings suggest that tamoxifen‐driven alterations in the tumor microenvironment, coupled with tumor‐intrinsic RACK7 loss, cooperatively promote T‐cell exhaustion. This highlights a potential therapeutic rationale for targeting the TIM3 axis in the context of prolonged tamoxifen treatment.

Supporting this idea, our results demonstrate that blocking the CEACAM1‐TIM3 interaction with an anti‐TIM3 antibody significantly reverses tumor growth associated with this tamoxifen resistance mechanism (i.e., tamoxifen plus RACK7‐depletion). Interestingly, our data further show that the combination of tamoxifen and anti‐TIM3 antibody yields a comparable therapeutic effect under both RACK7‐proficient and RACK7‐deficient conditions (Supplementary Figure S15A–D). These findings suggest that aside from patient groups exhibiting tamoxifen‐induced loss of RACK7, TIM3 blockade combined with tamoxifen treatment may also be beneficial during early stages of treatment. Nevertheless, this assumption requires validation in more refined preclinical and clinical models with comprehensive immune profiling. Thus, differentiating patients who are likely to be endangered by continued tamoxifen treatment from those who will benefit becomes increasingly critical. Notably, when relapsed ER^+^ patients are stratified based on their STING and CEACAM1 co‐expression levels (high and low), the prognostic effect of endocrine therapy is retained only among those with high STING and CEACAM1 co‐expression (Figure 6F), but is abolished among those with low co‐expression (Figure 6G). Thus, we propose that high co‐expression of these tamoxifen‐induced pro‐tumor factors could serve as a biomarker to identify at‐risk patients who might consider alternative modes of therapy, such as anti‐TIM3‐based ICB therapy. Given that the efficacy of anti‐PD1/anti‐TIM3 blockade in breast cancer remains underexplored, our preclinical data – showing a significant increase in PD1^+^/TIM3^+^ CD8^+^ T cells and PD‐L1–high macrophages – further support the potential of combining anti‐TIM3 and anti‐PD1 therapies to enhance treatment efficacy, particularly in relapsed ER^+^ patients. Nonetheless, the therapeutic potential of combined PD1 and TIM3 blockade in relapsed ER^+^ patients warrant further pre‐clinical investigation.

## 4 Methods

### 4.1 Cell Lines and Cell Culture

MCF7, T47D, MDA‐MB‐157 human breast cancer cells, along with 4T1 mouse breast cancer cells, were obtained from the laboratory of Dr. Chia‐Wei Li at Academia Sinica, Taipei, Taiwan. The cTMX‐MCF7 and cTMX‐T47D cell clones, along with their vehicle control (DMSO) clones, were obtained from the laboratory of Dr. Shen‐Yang Chen at Academia Sinica, Taipei, Taiwan. TS/A mouse breast cancer cells were obtained from the laboratory of Dr. Chandan Guha at Albert Einstein College of Medicine, NY, USA. HEK293T cells were obtained from the laboratory of Dr. Robert G. Roeder at The Rockefeller University, NY, USA. Cells were cultured in either phenol‐red‐free RPMI for T47D, DMEM/F12 for MCF7 and 4T1, or high‐glucose DMEM for TS/A. All media were supplemented with 10% FBS and 1% penicillin/streptomycin. The cells were maintained at 5% CO_2_ and 37°C in a humidified incubator. All media and supplements were obtained from Thermo Fisher Scientific. All the cell lines tested were free of Mycoplasma contamination.

### 4.2 Generation of cTMX, RACK7‐Knockdown and HA‐RACK7 Overexpressing Cell Lines

To establish the cTMX cell clones, MCF7 and T47D cells were cultured in appropriate growth media supplemented with a clinically relevant concentration of 5 µM tamoxifen (Selleckchem, Cat# S1238) and continuously passaged in the presence of tamoxifen for more than 6 months. Corresponding DMSO control cell clones were maintained in appropriate growth media supplemented with equal volumes of the vehicle control (DMSO) for the same number of passages. To establish RACK7‐knockdown (RACK7‐KD) cell lines, cells were infected with RACK7‐shRNA‐containing lentiviruses in medium containing polybrene (8 µg/mL). Twenty‐four hours post‐infection, cells were treated with 4–8 µg/mL puromycin to select for a pool of puromycin‐resistant clones. Knockdown efficiency was validated by immunoblotting. Stable clones were subsequently maintained in 1–2 µg/mL puromycin. RACK7‐shRNA‐containing lentiviral vectors were obtained from the National RNAi Core Facility (Academia Sinica, Taiwan) and prepared in accordance with standard protocols. The shRNA targeting sequences (with RNAi consortium ID) are as follows: human RACK7‐shRNA (ID: TRCN0000037995), mouse RACK7‐shRNA (ID: TRCN0000088516), and scramble control‐shRNA (ID: ASN0000000004). To establish doxycycline‐inducible HA‐tagged (HA‐RACK7) overexpressing cTMX‐T47D cell lines, the full‐length human *RACK7* cDNA was subcloned from the GFP‐ZMYND8 expression plasmid, a gift from Kyle Miller (Addgene plasmid # 65401; http://n2t.net/addgene:65401; RRID: Addgene_65401), into a 3xHA‐containing plasmid to insert an N‐terminal 3xHA‐tag into the *RACK7* (aka *ZMYND8*) gene. The doxycycline‐inducible 3xHA‐RACK7 expression plasmid was constructed by cloning the 3xHA‐RACK7 sequence into a Tet‐on lentivirus vector for doxycycline induction (Takara Bio, Cat# 631847). Stably HA‐RACK7‐overexpressing clones and the corresponding empty vector (EV) clones were generated by infecting cells with the respective lentiviruses. Stable HA‐RACK7‐T47D clones were then selected using 4 µg/mL puromycin and maintained in 2 µg/mL puromycin. HA‐RACK7 overexpression was validated by induction with 0.5–2 µg/mL doxycycline (Sigma) for 48 hours and immunoblot detection using an anti‐HA antibody. All stable clones tested were free of Mycoplasma contamination.

### 4.3 Cell Lysate Preparation and Immunoblotting

All experiments were conducted following standard protocols. Briefly, snap‐frozen cell pellets were resuspended in RIPA buffer (50 mM Tris‐HCl, pH 7.5, 150 mM NaCl, 1% Nonidet P40,0,5% sodium deoxycholate, 0.1% SDS) containing 1X complete EDTA‐free Protease Inhibitor Cocktail (Roche, Cat# 4693159001) and 1X PhosSTOP Phosphatase Inhibitor Cocktail (Roche, Cat# 4906845001). The cell lysates were then pulse sonicated for 15 pulses using a Bioruptor sonicator (Diagenode) and centrifuged at 4°C at 15,000×g for 20 minutes. The protein concentration in the supernatant was quantified using the Pierce BCA Protein Assay Kit (Pierce, Cat# 23325). Protein lysate was diluted in 1X SDS sample loading buffer and boiled at 95°C for 10 minutes. The protein samples were separated by SDS–PAGE, transferred to a poly(vinylidene difluoride) (PVDF) membrane, and subjected to immunoblotting as follows. PVDF membranes were blocked with either 5% formula milk, or 5% BSA (BioBasic, Cat# D0024) for phosphoproteins, at room temperature for 1 hour. The membranes were then incubated with primary antibodies overnight at 4°C, followed by the corresponding secondary antibodies at room temperature for 1 hour. Immunoblot bands were visualized using a UVP BioSpectrum Imaging System (Analytik Jena). All antibodies used are listed in Supplementary Table 1.

### 4.4 Quantitative Real‐Time PCR (RT‐qPCR)

Total RNA was extracted using the RNASpin Mini kit (Cytiva, Cat# GE25050071) according to the manufacturer's protocol. One microgram (1 µg) of total RNA was used to synthesize cDNA with the PrimeScript RT MasterMix (Takara Bio, Cat# RR036A) following the manufacturer's recommendations. qPCR was performed using the Power SYBR Green Master Mix (Thermo Fisher Scientific, Cat# 4368708), and Ct values were detected with the QuantStudio 5 RT‐PCR system (Applied Biosystems). Relative mRNA expression was calculated using the comparative Ct method (ΔΔCt). Log2 fold changes (Log2FC) were calculated using the 2^−∆∆CT^ method, with Ct values normalized to GAPDH for human genes and β‐actin for mouse genes. All RT‐qPCR primers used and their corresponding sequences are listed in Supplementary Table 2.

### 4.5 Immunoblot and RT‐qPCR to Quantify the Effects of Short‐Term Tamoxifen Treatment on the cGAS/STING and ISG Pathways

To assess the effects of tamoxifen treatment and/or RACK7 knockdown on the cGAS/STING and ISG pathways in ER^+^ and ER^−^ breast cancer cells, scramble shRNA (Control) or human RACK7‐shRNA (RACK7‐KD) transduced T47D (ER^+^) and MDA‐MB‐157 (ER^−^) cells were seeded into 60‐mm dishes and treated with either 5–10 µM tamoxifen or vehicle control (DMSO) for 48 hours. Cells were trypsinized, washed once with 1X phosphate‐buffered saline (PBS), and snap‐frozen in liquid nitrogen. The frozen pellets were then lysed with RIPA buffer and subjected to immunoblotting for the detection of cGAS/STING components. Alternatively, RNA was extracted from the cells and analyzed by RT‐qPCR for the detection of ISGs. To assess the effects of exogenous RACK7 overexpression on ISGs in cTMX cells, cTMX‐T47D cells transduced with either EV or HA‐RACK7 lentiviruses were maintained in 1 µg/mL doxycycline for 7 days to induce stable overexpression of HA‐RACK7. Cells were trypsinized, washed once with 1X PBS, and snap‐frozen in liquid nitrogen. The frozen pellets were then lysed with RIPA buffer and subjected to immunoblotting to detect HA‐RACK7 overexpression. Additionally, RNA was extracted from the cells and analyzed by RT‐qPCR to detect ISGs. To assess the effects of STING inhibition to the tamoxifen and RACK7 knockdown‐mediated induction of ISGs, T47D cells transduced with either scramble shRNA (Control) or human RACK7‐shRNA (RACK7‐KD) were seeded into 60‐mm dishes. These cells were pre‐treated with either 2 µM STING antagonist H‐151 (a gift from Dr. Liuh‐Yow Chen, Academia Sinica) or vehicle control (DMSO) for 24 hours, followed by two days of co‐treatment with either 10 µM tamoxifen or DMSO. Cells were trypsinized, washed once with 1X PBS, and RNA was extracted and analyzed by RT‐qPCR to detect ISGs. To assess the role of mitochondrial DNA (mtDNA) damage in the tamoxifen‐induced activation of the cGAS/STING‐IFN‐I pathway, RACK7‐KD T47D cells were seeded into 60‐mm dishes and depleted of mtDNA as previously described [82]. Briefly, cells were maintained in complete media supplemented with 4.5 mg/mL D‐glucose (Merck, Cat# 108337), 50 ng/mL ethidium bromide (Sigma, Cat# E‐8751), 100 µg/mL pyruvate (Sigma, Cat# P5280), and 50 µg/mL uridine (Sigma, Cat# U3003) for 48 hours. Subsequently, the cells were treated with either 10 µM tamoxifen or DMSO for another 48 hours. Cells were trypsinized, washed once with 1X PBS, and snap‐frozen in liquid nitrogen. The frozen pellets were lysed with RIPA buffer and subjected to immunoblotting to detect STING protein levels. Alternatively, RNA was extracted from the cells and analyzed by RT‐qPCR for the detection of ISGs. Whole‐cell DNA was extracted using the Quick Extract DNA extraction kit (Lucigen, Cat# QE09050) following the manufacturer's protocols. Depletion of mtDNA was validated by RT‐qPCR using primers targeting three distinct loci of mtDNA‐encoded genes (ND1, ATP6, and CYB). Primers targeting the genomic locus of the HBB gene were used as a normalization control. All RT‐qPCR primers, genomic primers, and antibodies used and their corresponding RRIDs are listed in Supplementary Table 1 and Supplementary Table 2.

### 4.6 Immunoblot and RT‐qPCR to Quantify the Effects of Short‐Term Tamoxifen Treatment on the Protein Stability of RACK7 and KDM5C

To assess the effect of tamoxifen on the stability of RACK7 and KDM5C proteins, a cycloheximide (CHX) chase assay (Sigma‐Aldrich, Cat# C7698) was performed using parental T47D cells. Cells were seeded into 60‐mm dishes and treated with either 10 µM tamoxifen or vehicle control (DMSO) for 48 hours. Protein synthesis was then inhibited by the addition of 100 µg/mL CHX for 0 (control), 2, 4, 5, 6, 7, and 8 hours post‐treatment. After each time point, cells were trypsinized, washed once with 1X PBS, and snap‐frozen in liquid nitrogen. Cell pellets were lysed with RIPA buffer and analyzed by immunoblotting to detect RACK7 and KDM5C proteins. To determine whether RACK7 and KDM5C are transcriptional targets of ER signaling, parental T47D cells were treated with estradiol (E_2_) (Selleckchem, Cat# S1709), the natural ER ligand. Cells were seeded into 60‐mm dishes and cultured in FBS‐free complete media for 6 hours prior to E_2_ treatment. Cells were then treated with E_2_ for 24 and 48 hours, trypsinized, washed with 1X PBS, and snap‐frozen in liquid nitrogen. RNA was extracted from the cells and analyzed by RT‐qPCR to measure the mRNA levels of RACK7 and KDM5C. The mRNA of PS2, a well‐established ER target gene, was quantified as a positive control.

### 4.7 RNA Sequencing (RNA‐seq) and Data Analysis

For RNA‐seq, RNA was extracted with RNAspin Mini Kit (Cytiva, Cat# GE25050071) according to the manufacturer's protocols. Sequencing libraries were generated using the TruSeq Stranded mRNA Library Prep Kit (Illumina), following the manufacturer's protocols. The products were enriched via PCR and purified using the AMPure XP system (Beckman Coulter). The libraries were qualified by Qsep400 System (Bioptic Inc.) and quantified with a Qubit 2.0 Fluorometer (Thermo Fisher Scientific). The qualified libraries were then sequenced on an Illumina NovaSeq 6000 platform with 150 bp paired‐end reads. For RNA‐seq data analysis, raw reads were processed by Trimmomatic to remove adapters and low‐quality sequences (cutoff = Q20). The processed reads were aligned to the human genome (GRCh38) using Hisat2. Gene expression levels (read count and TPM) were measured using Stringtie with human gene annotation downloaded from Ensembl (release 104). Differentially expressed genes (DEGs) were identified using the DESeq2 R package. Kyoto Encyclopedia of Genes and Genomes (KEGG) pathway enrichment analysis was performed using WebGestalt with FDR < 0.05. The Gene Set Enrichment Analysis (GSEA) was conducted with GSEA v4.2.3 software, using 1,000 permutations. The list of dysregulated pathways, along with their corresponding normalized enrichment scores from the GSEA, was summarized using GraphPad Prism 9.5.0. Differential Expression Gene (DEG) volcano plots were created using Microsoft Excel, and enrichment plots were produced with the GSEA software version 4.2.3.

### 4.8 Flow Cytometry to Quantify Tamoxifen's Effects on Tumor Cell‐Induced T‐Cell Activation in Co‐Culture Systems

Whole blood samples from healthy adults were sourced from AllCells (Alameda, CA, USA), with all donors providing informed consent under their Institutional Review Board (IRB)‐approved program. Human peripheral blood mononuclear cells (PBMCs) were isolated by density gradient centrifugation using Ficoll‐Paque following the manufacturer's instructions. Briefly, whole blood samples were pre‐diluted 1:1 with 1X PBS without Ca^2+^ and Mg^2+^ (Gibco, Cat# J67802‐AP) and carefully layered on an equal volume of Ficoll‐Paque (Cytiva, Cat# 17144003). The PBMC fraction was isolated following centrifugation at 800×g for 20 minutes at room temperature with the brake off. Subsequently, CD3^+^ T‐cells were positively isolated using the MACS system (Miltenyi Biotec, Cat# 130‐097‐043) according to the manufacturer's instructions. Isolated T‐cells were cultured in phenol‐free RPMI supplemented with 10% heat‐inactivated FBS, 25 µL/mL CD3/CD28 T‐cell activator (Stem Cell Technologies, Cat# 10971), and 10 ng/mL IL‐2 (Stem Cell Technologies, Cat# 78036) for 3 days prior to co‐culture with tumor cells. In parallel, parental‐T47D cells were pre‐treated with either 10 µM tamoxifen or vehicle control (DMSO) for 2 days. DMSO‐T47D and DMSO‐MCF7 cells were pre‐treated with vehicle control (DMSO), while the cTMX‐T47D and cTMX‐MCF7 cells were pre‐treated with 10 µM tamoxifen for 2 days prior to co‐culture. T‐cells were then stained with CellTrace Violet (CTV) dye (BioLegend, Cat# 425101) and co‐cultured with either DMSO‐pre‐treated parental‐T47D, TMX‐pre‐treated parental‐T47D, DMSO‐T47D, DMSO‐MCF7, cTMX‐T47D, or cTMX‐MCF7 cells at a 1:5 T‐cell to tumor cell ratio for an additional 2 days in the presence of 25 µL/mL CD3/CD28 activator, 10 ng/mL IL‐2, and either DMSO or 10 µM tamoxifen, as indicated. The supernatants were harvested, and T‐cells were resuspended in ice‐cold, serum‐free PBS. CD3^+^ T‐cells were incubated with an antibody cocktail containing CD44‐PE (BioLegend, Cat# 103023), CD69‐FITC (BioLegend, Cat# 310903), and CD25‐APC (BioLegend, Cat# 302609) antibodies for 50 minutes at 4°C with shaking. The antibody against CD44 was used together with antibodies against CD69 and CD25 to gate for CD3/CD28‐stimulated T‐cell populations. Flow cytometry was performed using an Attune CytPix Flow Cytometer (Thermo Fisher Sc), and data were analyzed using the FlowJo Software (BD Life Sciences). Representative gating strategies are illustrated in Supplementary Figure S4.

### 4.9 Syngeneic Tumor Mouse Models

The *RACK7* gene was knocked down in murine ER^+^ TS/A or TNBC 4T1 cells using mouse RACK7‐shRNA, with scramble‐control shRNA as the control. For the TS/A and 4T1 tumor models, viable control and RACK7‐knockdown (RACK7‐KD) TS/A or 4T1 cells, at a target of 1×10^5^ cells/mouse, were orthotopically implanted into the mammary fat pad of each female BALB/c mouse aged 6–8 weeks. Treatment was initiated on Day 1, which is 1‐day post‐tumor implantation, as follows. The vehicle (corn oil) and tamoxifen at 2 mg/mL, formulated in corn oil, were each intraperitoneally (IP) administered at 0.05 mL per mouse once every other day for a total of 12 dose administrations (QOD × 12 doses). The animals received a tamoxifen dose of 0.1 mg/mouse with an approximate 5 mg/kg dosage using an average weight of 20 g per mouse. The IgG isotype control (ichorbio, Cat# ICH2244) and anti‐mouse TIM‐3 (Bio X Cell, Cat# BE0115) antibodies, each at 10 mg/kg, were IP administered twice per week for 3 consecutive weeks with a total of 6 dose administrations (BIW × 3 weeks), starting on Day 7 post‐tumor implantation. On days of co‐administration, tamoxifen was administered first, followed by antibody administration 1 hour later. Body weight and tumor volume were measured thrice per week beginning 4 days post‐tumor implantation, and continued for 4 weeks, or until the mean tumor volume reached 1500 mm^3^, whichever came first. Tumor volume (mm^3^) was measured using a caliper and calculated according to the prolate ellipsoid formula, by length (L) × width (W) × width (W) × 0.5. At study termination, tumors were harvested, weighed, and processed into single cells for flow cytometric (TS/A) or CyTOF (4T1) immune profiling of the tumor microenvironment. All animal experiments were reviewed, approved, and conducted in accordance with the Institutional Animal Care and Use Committee (IACUC) guidelines for each collaborating group, namely, Pharmacology Discovery Services Taiwan, Ltd. (protocol no. ON003‐07142023), Academia Sinica (protocol no. 24‐03‐2151), and Albert Einstein College of Medicine (protocol no. 00001055).

### 4.10 Flow Cytometry for Profiling the Tumor Immune Microenvironment in TS/A Xenografts

Single‐cell suspensions were prepared from tumors harvested on day 30. The tumors were weighed upon excision, and 0.2 grams of tumor tissue were chopped into small pieces and enzymatically digested in a cocktail of collagenase I and IV (100 U/mL each, Gibco, Cat#17100‐017; Cat#17104‐019) and 40 U/mL of DNase I (Cat# 10104159001) in RPMI by stirring at 37°C for 30 minutes. All cell suspensions were passed through a 70‐µm cell strainer before staining. All flow antibodies were used at 1:100 dilutions for staining. NIR Zombie Live/Dead Fixable dye (Biolegend, Cat# 77184) was used to exclude dead cells from the analysis. Anti‐mouse CD16/32 (clone 2.4G2) Fc block antibody was used for blocking. The following antibodies were used for surface staining: Anti‐CD45_Alexa Fluor700 (clone 30‐F11), Anti‐CD3_APC Fire810 (clone 17A2,) Anti‐CD4_PE Fire640 (clone GK1.5), Anti‐CD8a_eFluor450 (clone 53–6.7), Anti‐PD1_PE‐Cy7 (clone EH12.1), Anti‐TIM3_PerCP‐eF710 (clone RMT3‐23), Anti‐PD‐L1_BUV737 (clone MIH5), Anti‐CD66a (CEACAM1)_APC (clone Mab‐CC1) antibodies. Single‐stained eBeads (UltraComp eBeads Compensation beads; Invitrogen, Cat# 01‐2222‐41) were used for compensation, except for the unstained sample for PMT voltage setting and Fixable Viability Dye for which cells were used. Flow cytometry was performed using an Aurora Flow Cytometer (Cytek Biosciences), and data were analyzed with FlowJo FACS Analysis Software (Tree Star). Representative gating strategies are illustrated in Supplementary Figure S7.

### 4.11 Single‐Cell Mass Cytometry (CyTOF) for Profiling the Tumor Immune Microenvironment in 4T1 Xenografts

Samples were processed following the established protocol [83, 84] with minor modifications. Briefly, tumors were finely chopped into pieces measuring approximately 1–1.5 mm using scissors. These fragments were then resuspended in papain (0.6 mg/mL) and incubated at 37°C for 1 hour to facilitate digestion. Subsequently, the digested tissue was filtered through a 70 µm cell strainer and rinsed with HBSS, followed by centrifugation at 450 × g for 5 minutes at 4°C. The resulting pellets were treated with ACK lysis buffer to lyse red blood cells for 5 minutes at room temperature. This process was halted by adding an equal volume of HBSS. After another round of centrifugation under identical conditions, the supernatant was removed, leaving behind single cells that were then resuspended in MACS cell storage buffer (Miltenyi Biotec). Approximately 1×10^7^ dissociated single cells were stained for viability using cisplatin (Fluidigm, Cat#201064), followed by fixation with 1.5% paraformaldehyde (Electron Microscopy Sciences, Cat# 15710) for 10 minutes at room temperature. Subsequently, the cells were washed twice with Cell Staining Medium (CSM) [PBS supplemented with 0.5% bovine serum albumin (BSA; Sigma‐Aldrich, Cat# A3059)]. The cell samples were then subjected to barcoding according to the manufacturer's protocol (Fluidigm, Cat# 201060). Briefly, formaldehyde‐fixed cell samples were resuspended in 1X Barcode Buffer and labelled with the corresponding barcodes. The samples were then stained with metal‐conjugated antibodies against surface markers for 1 hour. After staining, samples were washed once with CSM and then permeabilized with 1X Foxp3 Fixation/Permeabilization Solution (FPS) on ice for 30 minutes (eBioscience, Cat# 00‐5523‐00). Following permeabilization, samples were washed twice with 1X FPS and stained with antibodies against intracellular molecules for 1 hour. Cells were washed once with 1X FPS, and incubated with an iridium‐containing DNA intercalator (Fluidigm, Cat# 201192A) in PBS containing 1.5% paraformaldehyde at room temperature for 20 minutes. After intercalation and fixation, the cell samples were washed once with CSM and twice with water before analysis using a mass cytometer (Fluidigm). Finally, cells were resuspended in MilliQ water containing EQ Four Element Calibration Beads (Fluidigm) for normalization. Data were acquired on a CyTOF2 mass cytometer (Fluidigm). For antibody conjugations, antibodies in carrier‐free PBS were conjugated to metal‐chelated polymers according to the manufacturer's recommendations (Fluidigm). All conjugated antibodies are listed in Supplementary Table S3.

### 4.12 CyTOF Data Analysis

CyTOF data were normalized and debarcoded using Premessa (https://github.com/ParkerICI/premessa) as previously described [83, 84]. Batch‐related staining variations were corrected with CytofBatchAdjust (https://github.com/CUHIMSR/ CytofBatchAdjust) [85]. Gating was performed as shown in Supplementary Figure S11. Uniform Manifold Approximation and Projection (UMAP) of live CD45^+^ population was conducted in Cytobank (https://www.cytobank.org/) using 35 immune cell lineage markers, including B220, CCR2, CD103, CD11b, CD11c, CD127, CD137, CD172a, CD19, CD25, CD274, CD3, CD4, CD44, CD47, CD62L, CD64, CD80, CD86, CD8a, CTLA4, F4/80, GR1, LAG3, Ly6C, Ly6G, MHCII, NK1.1, NKp46, PD1, pDCA1, TCRb, TCRgd, TIM‐3, and XCR1. The parameters for UMAP included all events, with a number of neighbors set to 15 and a minimum distance of 0.01. CD3^+^CD8^+^ populations were identified by high CD3 and CD8 expression in UMAP results. The t‐distributed Stochastic Neighbor Embedding (t‐SNE) analysis of CD3^+^CD8^+^ populations was performed in Cytobank using 10 T‐cell exhaustion markers: CD137, CD44, CTLA4, EOMES, LAG3, LEF1, PD1, T‐bet, TIM‐3, and TOX. The t‐SNE parameters included all events, 750 iterations, and a perplexity of 30. The outliers were identified using the ROUT method (Q = 1%) and excluded from the analysis. The percentages of immune cell populations and CD8^+^ T cell subpopulations were analyzed based on UMAP and t‐SNE results. Statistical significance was assessed using one‐way ANOVA with Tukey's post hoc test in Prism, with *p < 0.05 and **p < 0.05 considered significant.

### 4.13 Statistical and Database Analyses

Public patient datasets as indicated were re‐analyzed using the cBioPortal [45], BioJupies [86], WebGestalt [87], and CTGS [51] web tools. For constructing TMB histograms and conducting survival analysis that stratify between TMB classes and endocrine therapy status, data from the METABRIC database were utilized. Patients were categorized based on their TMB levels; individuals were assigned to either a high TMB group (≥10 mutations per megabase [mt/Mb]) or a low TMB group (<10 mt/Mb), in accordance with US FDA‐approved cutoffs [88]. For the survival analysis of ER^+^ patients, differentiated by low vs high ISG geneset scores, patient data from both the METABRIC and TCGA datasets accessed via the CTGS web application were used. The scRNA‐seq dataset of tamoxifen‐treated tumor biopsies was obtained and reanalyzed from dbGaP, with accession #phs003186.v1.p1. All statistical analyses were conducted using GraphPad Prism 9.5.0. The specific statistical tests, sample size, and significance values used for each presented data is outlined in their corresponding figure legends.

## Funding

Academia Sinica (AS‐VTA‐111‐13, AS‐CDA‐112‐L03, AS‐VTA‐113‐17, AS‐GCS‐114‐L10, AS‐FILBD‐114‐03, AS‐NTU‐114‐09, AS‐GCS‐115‐L02, AS‐BRTP‐115‐02, AS‐VTA‐115‐12‐3) and National Science and Technology Council in Taiwan (110‐2628‐B‐001‐017, 111‐2628‐B‐001‐010, 112‐2628‐B‐001‐002, 112‐2320‐B‐001‐013‐MY3, 113‐2923‐B‐001‐004‐MY3).

## Conflicts of Interest

The authors declare no conflicts of interest.

## Supporting information




**Supporting File 1**: advs77007‐sup‐0001‐SuppMat.pdf.

**Supporting File 2**: advs77007‐sup‐0002‐TableS1.pdf.

**Supporting File 3**: advs77007‐sup‐0003‐TableS2.pdf.

**Supporting File 4**: advs77007‐sup‐0004‐TableS3.pdf.

## Data Availability

The data that support the findings of this study are openly available in NCBI at https://dataview.ncbi.nlm.nih.gov/object/PRJNA1175753?reviewer=kbbvtqgkipba2pv1en2svu158q, reference number 1175753.
