# Supplementary material for: Dual Role of Tamoxifen in Enhancing STING and CEACAM1 Expression to Prime a Favorable Tumor Microenvironment for Anti‐TIM3 Immunotherapy
Source: Adv Sci (Weinh). 2026 Aug 17:e03303. Online ahead of print. doi: 10.1002/advs.202503303 (PMC13479595; doi:10.1002/advs.202503303)
Supplement: Supplementary file 1 [file ADVS-9999-e03303-s001.pdf]

Supporting Information

Dual Role of Tamoxifen in Enhancing STING and CEACAM1 Expression to Prime a Favorable Tumor Microenvironment for Anti-TIM3 Immunotherapy

Marvin Angelo E Aberin, Saurabh Singh, Soumya Chatterjee, Guang-Zhi Sui, Yi-Fu Wang, Ya-Ting Lu, Yu-Ling Lee, Kun-Yuan Lin, Joy Khag, Ta-Yu Liu, Shao-Han Chang, Wai-Mui Cheung, Hsiao-Chin Hong, Ren-Jun Hsu, Chen-Yang Shen, Chia-Wei Li, Weng-Lang Yang, Yao-Ming Chang, Shih-Yu Chen, Chandan Guha, Shu-Ping Wang**

Figure S1 (related to Figure 1)

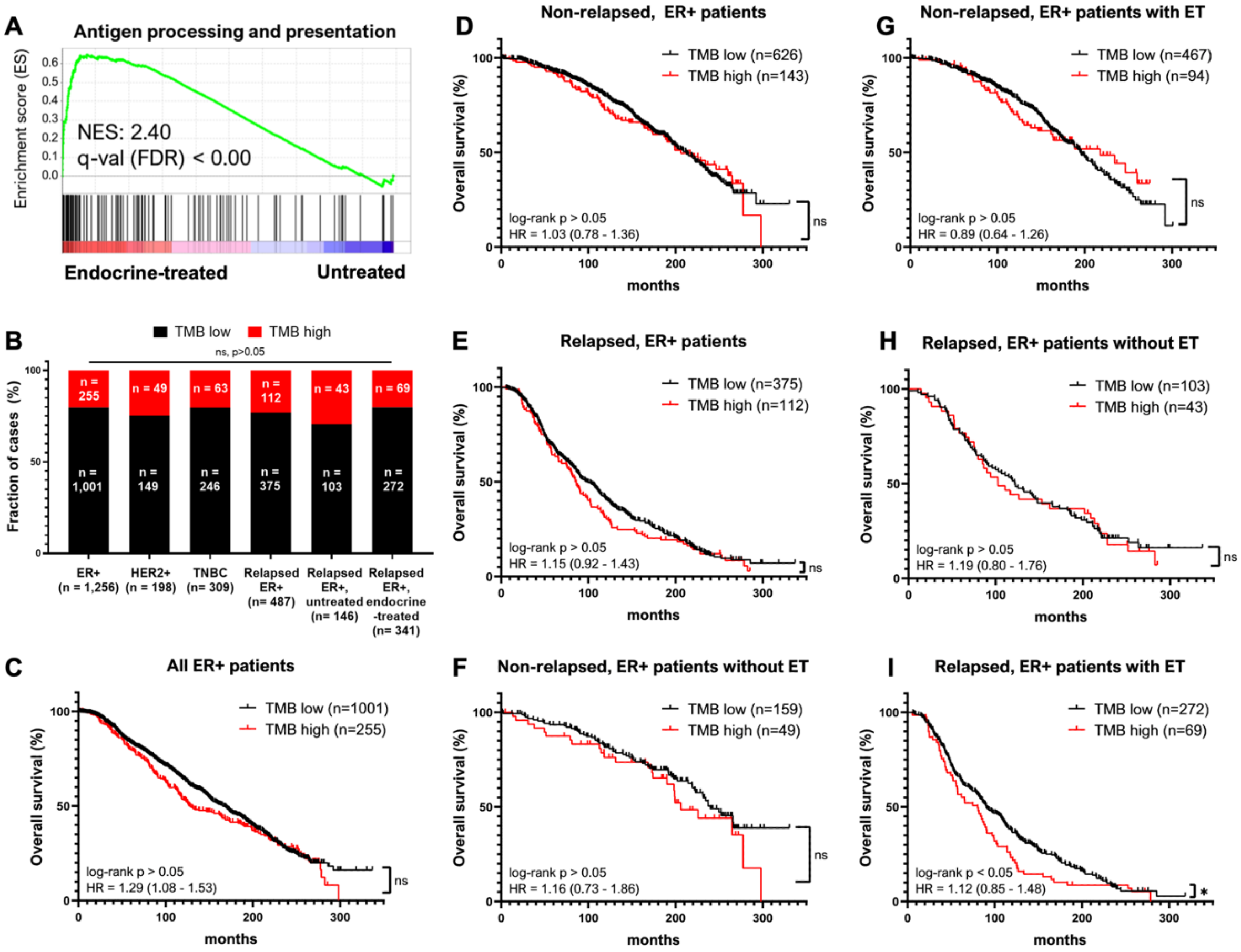

Figure S1 (related to Figure 1). Relapsed ER$^+$ tumors exhibit a comparable TMB profile to ICB-amenable TNBCs.

A. Enrichment score plot of 'Antigen processing and presentation' pathway (database: GO Biological processes) from Gene-set enrichment analysis (GSEA) using the relapsed, ER$^+$ patients receiving endocrine therapy treatment compared with those who did not receive endocrine therapy. Data were obtained from the cBioPortal webtool (Breast cancer dataset: METABRIC, Nature 2012 & Nature Communications 2016, n=2,509). **B.** Distribution of low (≤10) and high (>10) non-synonymous tumor mutational burden (TMB) across different breast cancer subtypes (ER$^+$, HER2$^+$, TNBC), including relapsed ER$^+$ tumors with or without endocrine treatment. **C–I.** Kaplan–Meier survival curves of ER$^+$ breast cancer patients with low and high TMB status under different conditions. All ER$^+$ patients (C), All non-relapsed ER$^+$ patients (D), All relapsed ER$^+$ patients (E), Non-relapsed ER$^+$ patients without endocrine therapy (F), Non-relapsed ER$^+$ patients with endocrine therapy (G), Relapsed ER$^+$ patients without endocrine therapy (H), Relapsed ER$^+$ patients with endocrine therapy (I). ER$^+$, ER-positive; HER2$^+$, HER2-positive; TNBC, triple negative breast cancer; ET, endocrine therapy; FDR, false discovery rate; NES, normalized enrichment score.

Figure S2 (related to Figure 1)

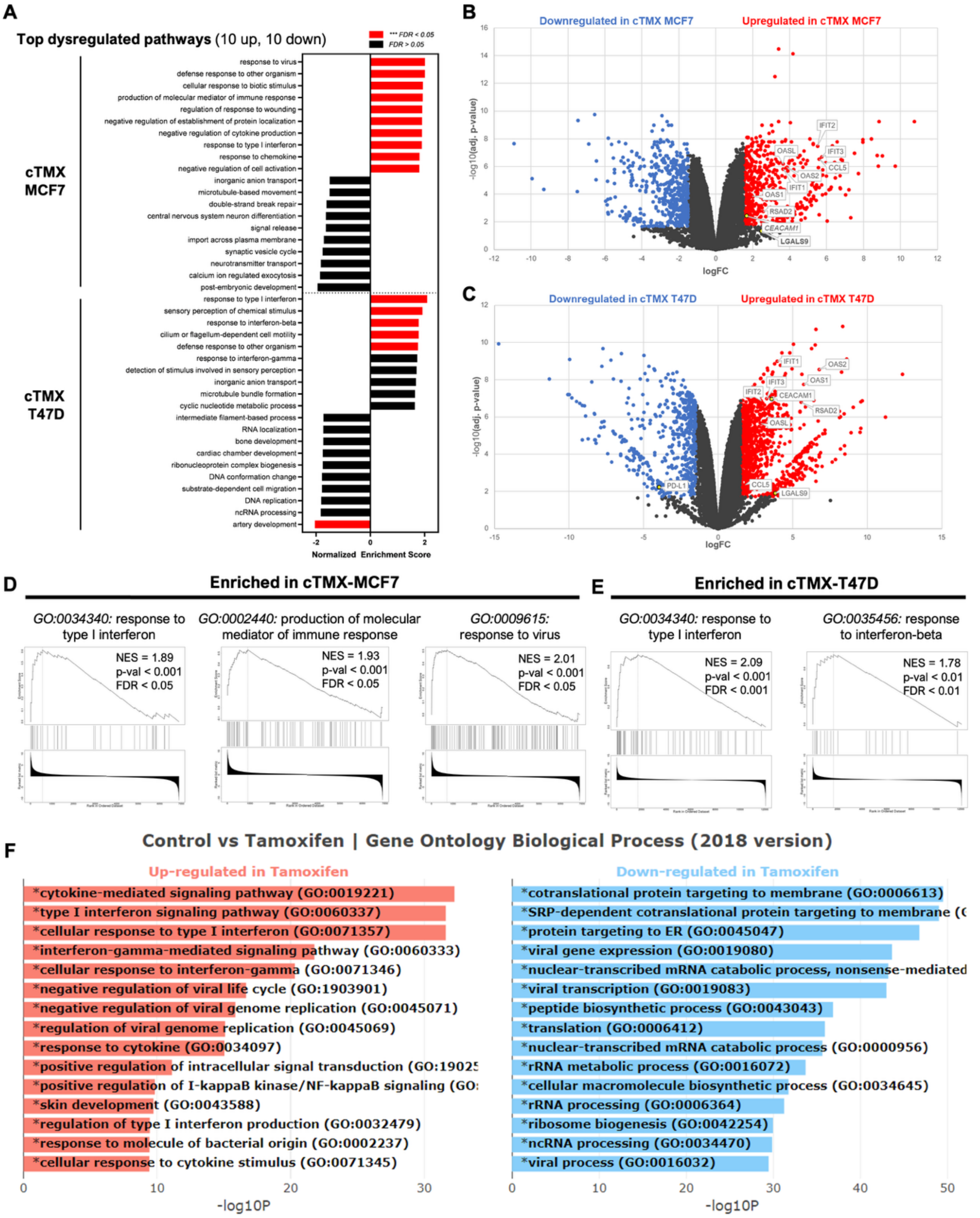

Figure S2 (related to Figure 1). Long-term tamoxifen treatment activates type I IFN-signaling and its associated ISGs.
A. Top 10 significantly inhibited and activated (FDR <0.05) pathways in cTMX MCF7 and T47D cells. **B** and **C.** Volcano plot showing differentially expressed genes (DEGs) in cTMX MCF7 (B) and T47D (C) cells relative to control. Data presented as logFC and –log10(adj. p-value) of each gene. **D** and **E.** GSEA plots of immunomodulatory and type I-IFN signaling-associated pathways activated in cTMX MCF7 and T47D cells. **F.** Enrichment analysis showing activation of type I-IFN signaling-associated pathways among T47D xenografts treated with tamoxifen relative to control xenografts. Data was accessed and reanalyzed from GSE80619 using the BioJupies webtool. cTMX, chronic tamoxifen.

Figure S3 (related to Figure 1)

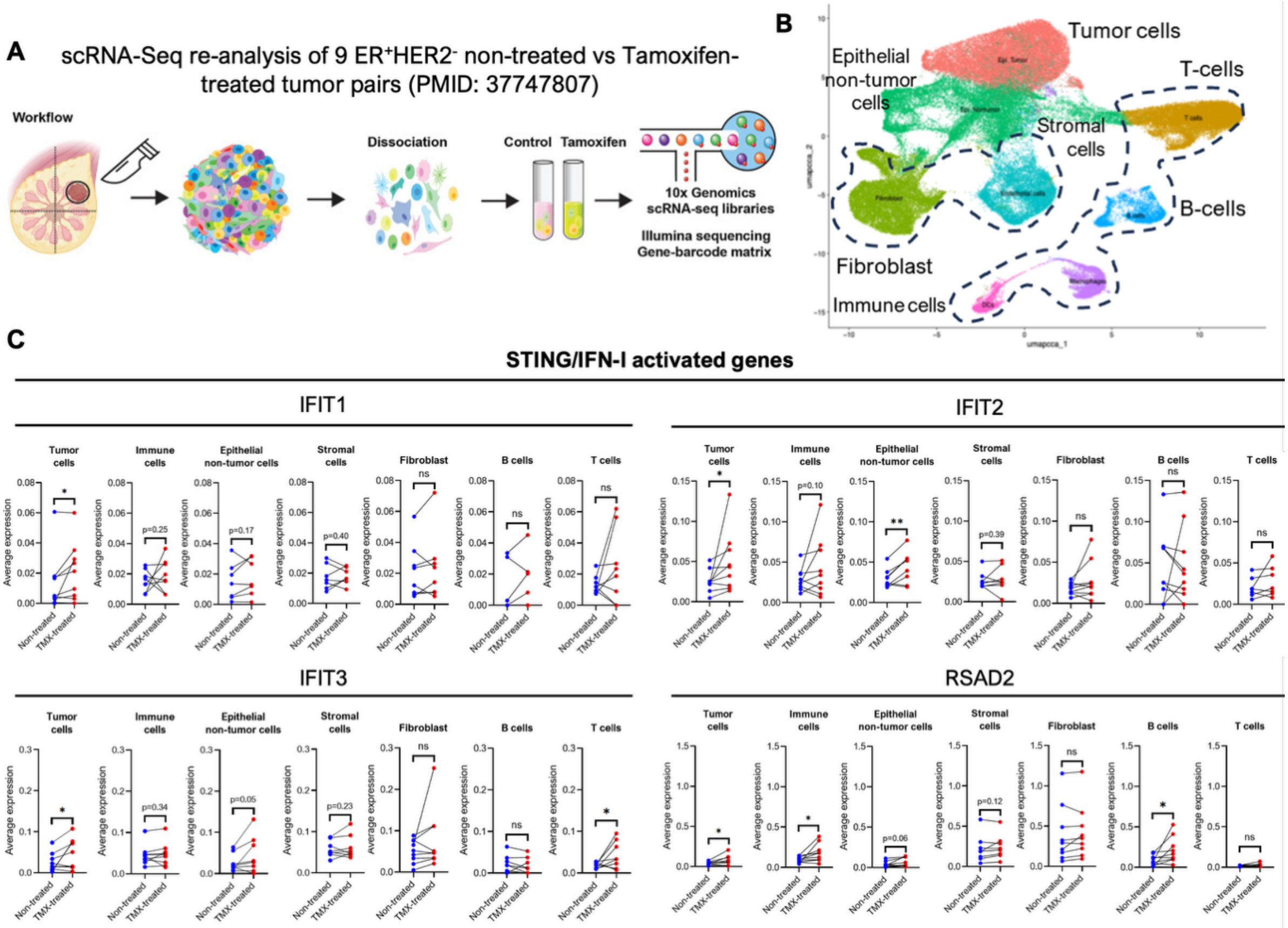

Figure S3 (related to Figure 1). Tamoxifen treatment selectively activates type IFN-I associated ISGs in tumor cells.
A. Experimental workflow of ex vivo tamoxifen treatment of paired tumor biopsies from ER$^+$ patients. Figure is adapted from (PMID: 37747807). **B.** UMAP re-clustering of patient-derived single cells identifies distinct clusters of tumor, epithelial non-tumor, stromal, fibroblast, and immune cells (i.e., T-cells, B-cells) in the tumor microenvironment **C.** Paired gene expression of STING/IFN-I activated genes between non-treated vs. tamoxifen-treated tumor biopsies.

Figure S4 (related to Figure 2)

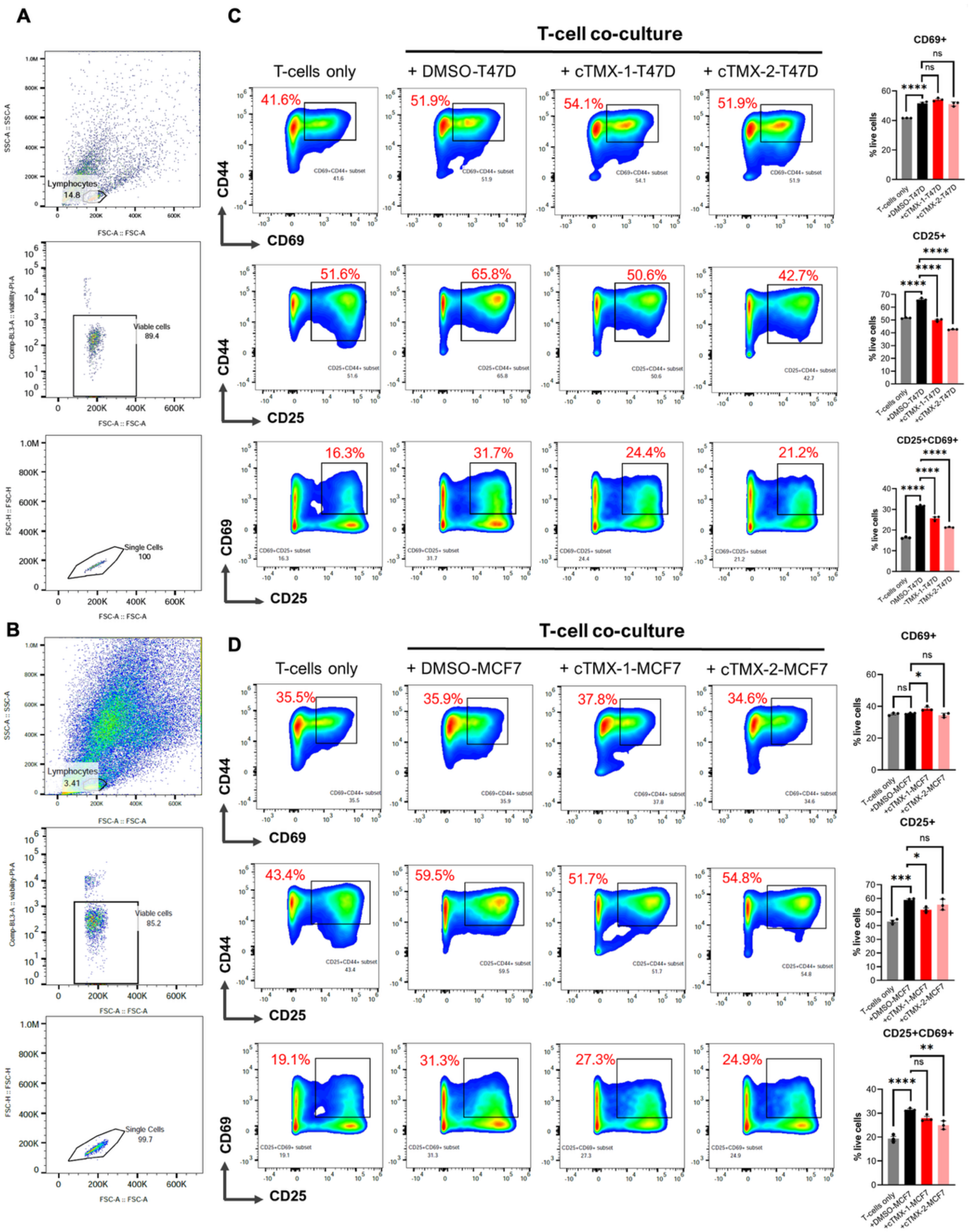

Figure S4 (related to Figure 2). Tamoxifen induces T-cell exhaustion in an in vitro tumor and T-cell co-culture system.
A and **B.** Representative gating strategy in the flow cytometry analysis of in vitro co-culture system. PI-negative cells were gated as viable T-cells from the total lymphocyte population. T-cells only group without tumor cell co-culture (A). Tumor and T-cell co-culture group (B). **C** and **D.** Representative density plots (left) and quantification (right) of $CD44^+CD69^+$, $CD44^+CD25^+$, and $CD25^+CD69^+$ T-cell populations co-cultured with DMSO-T47D, cTMX-1-T47D, cTMX-2 T47D cells (C) and DMSO-MCF7, cTMX-1-MCF7, cTMX-2 MCF7 cells (D). Data is presented as mean % of live cells ± SEM, n=3, p-values are calculated using one-way ANOVA followed by Tukey post-hoc test, *$p<0.05$, **$p<0.005$, ***$p<0.0005$, ****$p<0.0001$.

Figure S5 (related to Figure 3)

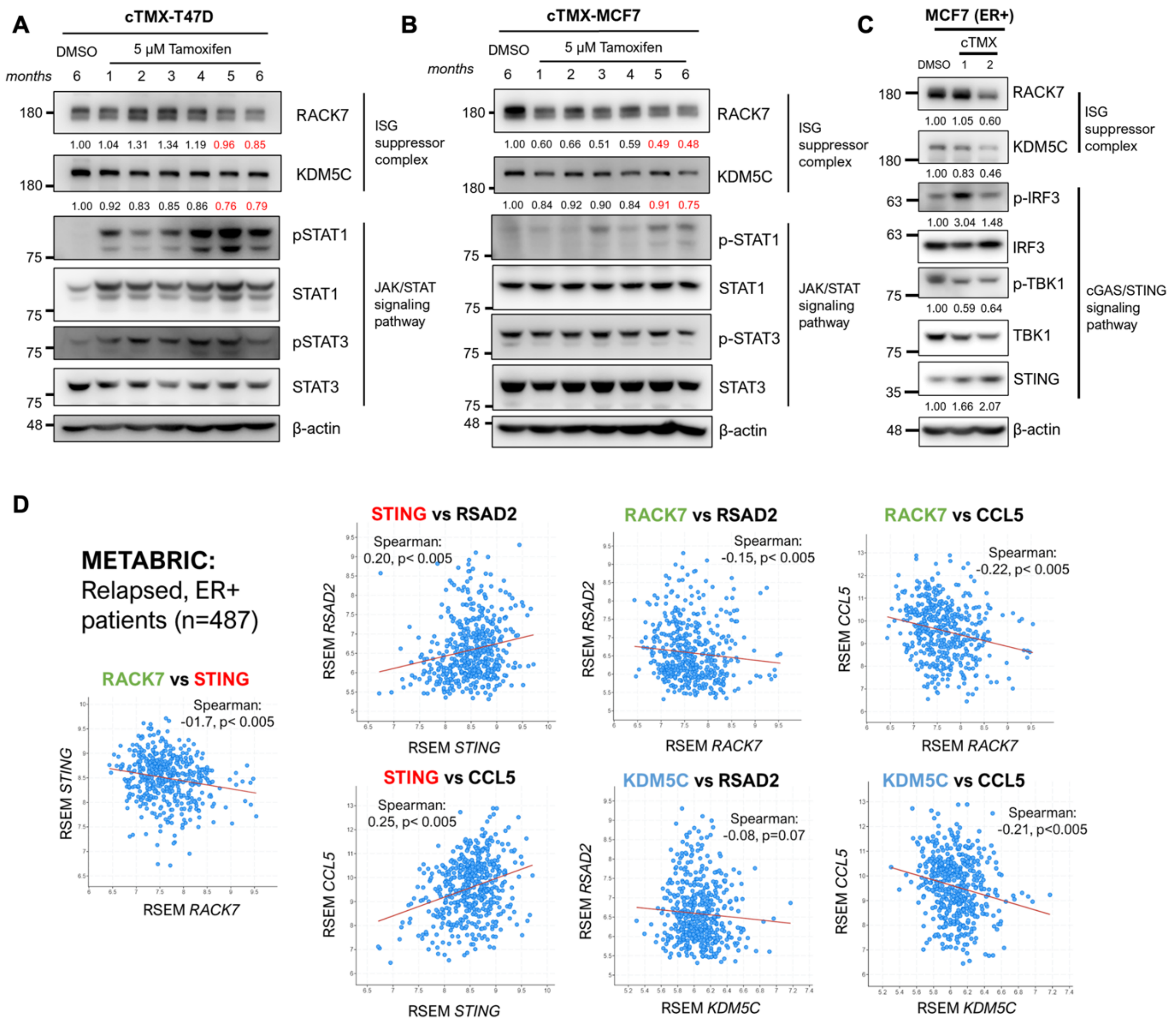

Figure S5 (related to Figure 3). Long-term tamoxifen treatment triggers the activation of JAK/STAT pathway, as well as the loss of the RACK7/KDM5C ISG suppressor complex. **A** and **B.** The ER-positive T47D (A) and MCF7 (B) breast cancer cells were chronically treated with 5 μM tamoxifen for 6 months. These chronically tamoxifen-treated cells (designated cTMX-T47D and cTMX-MCF7 cells) were collected monthly and subjected to immunoblotting. Immunoblotting revealed alterations in protein expression and/or phosphorylation status of components in the JAK/STAT signaling pathway and the RACK7/KDM5C complex following chronic tamoxifen (cTMX) treatment. **C.** Alterations in protein expression and/or phosphorylation status of components of the histone H3K4 demethylase complex (upper) and cGAS/STING pathway (lower) following chronic tamoxifen (cTMX) treatment. Immunoblotting showing the expression of the indicated total or phosphorylated proteins in control and two independent cTMX-MCF7 clones. The levels of β-actin serve as loading control. Numbers below the panels of the immunoblots indicate the densitometric values normalized to the corresponding control lane. **D.** Spearman correlation analysis of RACK7 with STING, as well as STING, RACK7, and KDM5C with selected ISGs, using data from the METABRIC public database (accessed via cBioPortal).

Figure S6 (related to Figure 3)

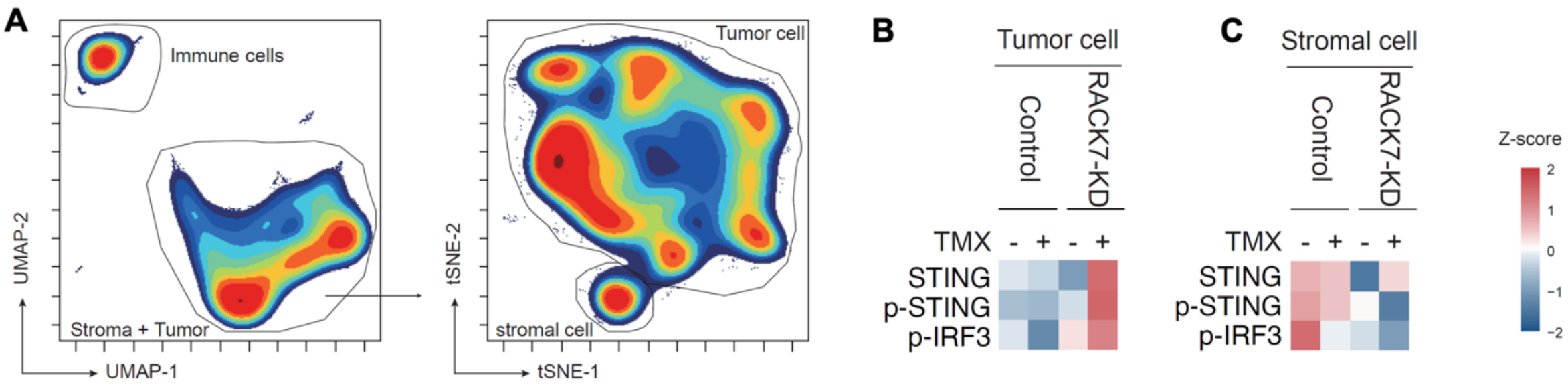

Figure S6 (related to Figure 3). Tamoxifen treatment activates cGAS/STING pathway in vivo.
A. UMAP (left) and tSNE (right) analysis showing distinct immune cell, stromal, and tumor cell population identified from harvested tumor samples. **B** and **C**. Heatmap comparison of STING, p-STING, and p-IRF3 signals in tumor (B) and stromal (C) cell populations between Control and RACK7-KD groups, with or without tamoxifen treatment.

Figure S7 (related to Figure 4)

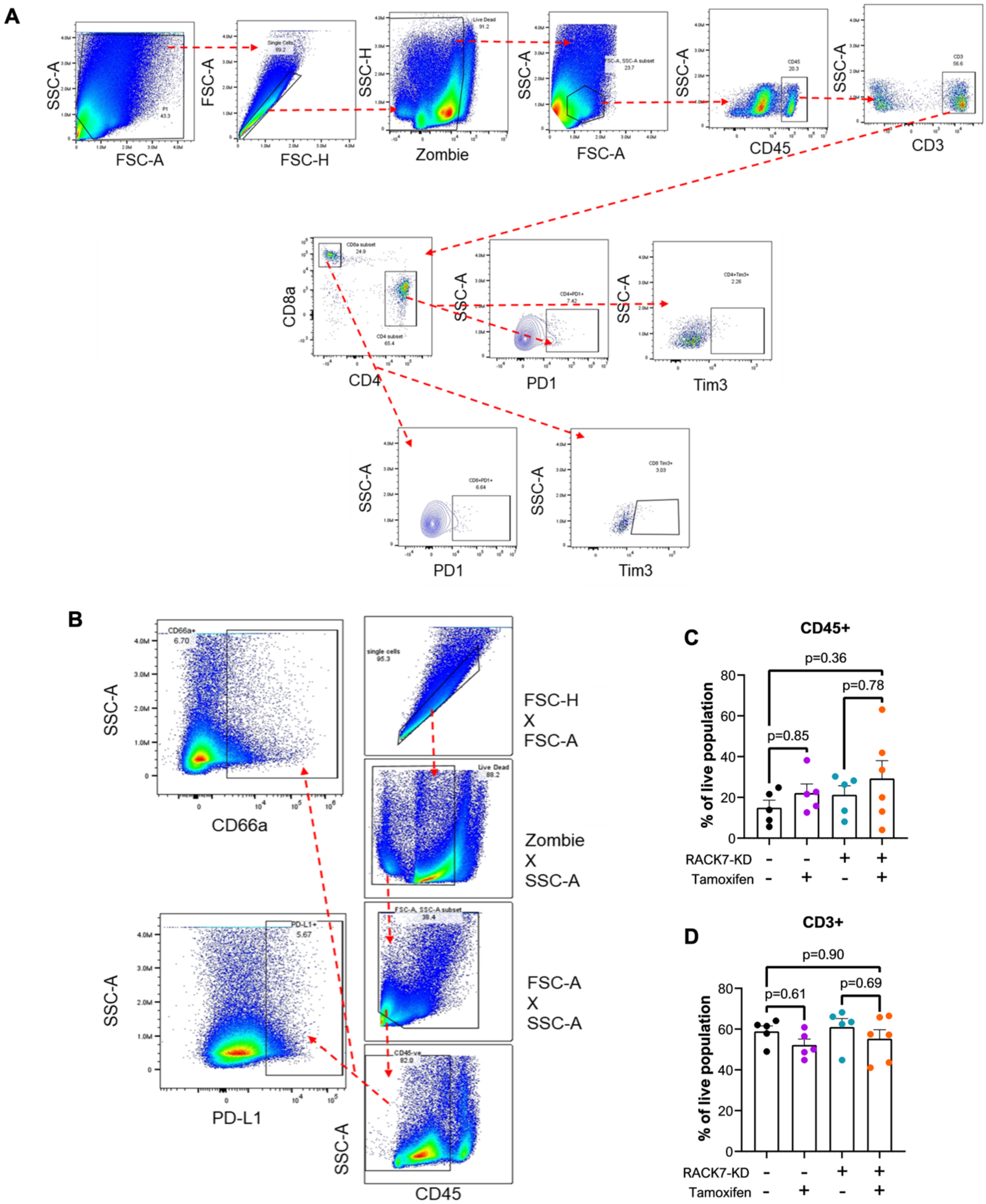

Figure S7 (related to Figure 4). The combined treatment of tamoxifen and RACK7 knockdown alter CD45+ immune cell population in the TS/A tumor microenvironment. **A.** Gating strategy for CD4 and CD8 T-cell populations. **B.** Gating strategy for the CD45-negative tumor cell population. **C** and **D.** The intratumoral abundance of CD45-positive ($CD45^+$, shown in C) immune cell or CD3-positive ($CD3^+$, shown in D) T cell populations in TS/A-Control or TS/A-RACK7-KD tumors, with or without tamoxifen treatment.

Figure S8 (related to Figure 4)

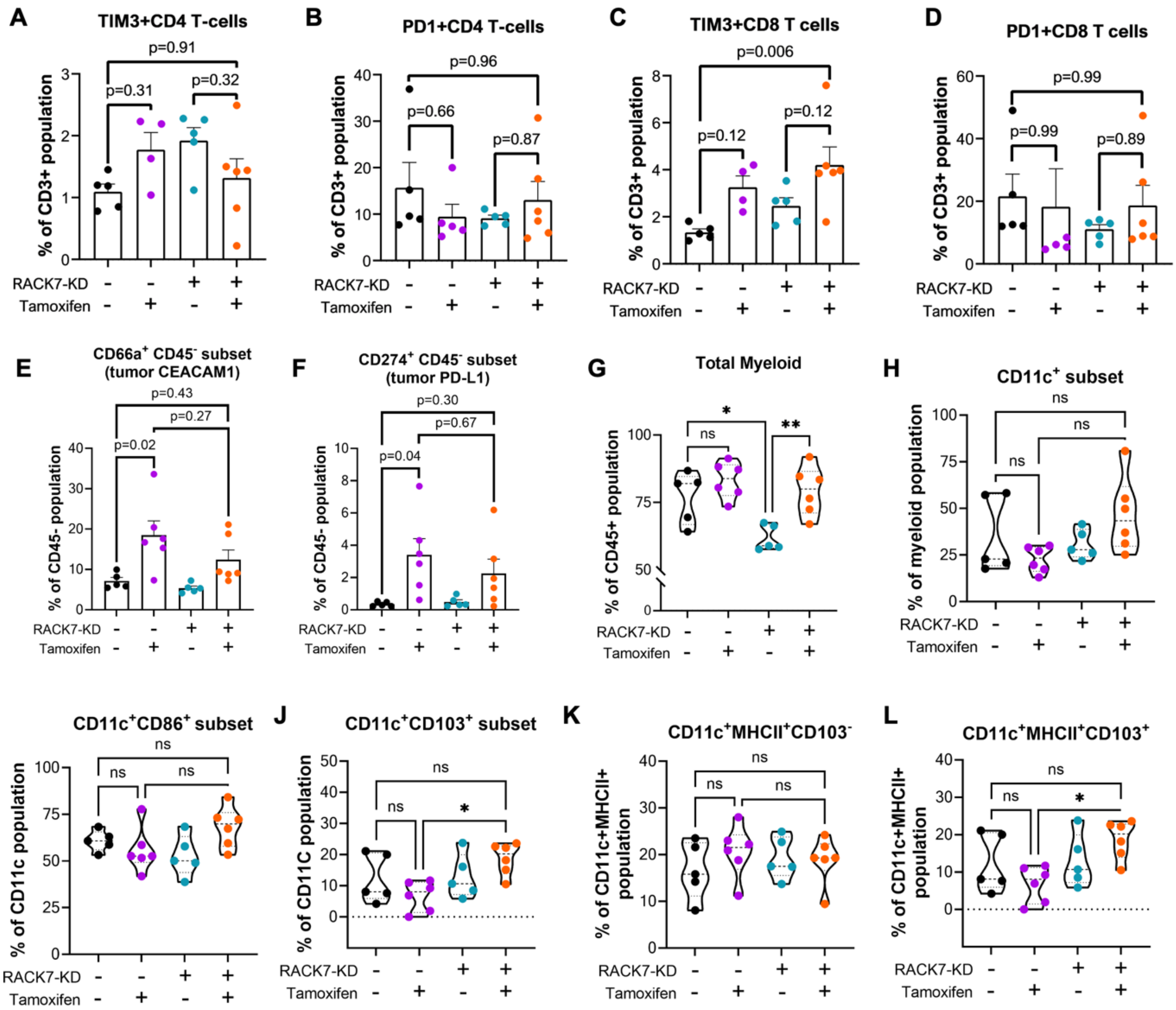

Figure S8 (related to Figure 4) The combined treatment of tamoxifen and RACK7 knockdown induce CD8^{+} T-cell exhaustion and promotes TS/A tumor growth in vivo.
A and **B.** Abundance of intratumoral TIM3-positive (TIM3^{+}, shown in A) or PD1-positive (PD1^{+}, shown in B) CD4^{+} T cell populations in TS/A-Control or TS/A-RACK7-KD tumors, with or without tamoxifen treatment. **C** and **D.** Abundance of intratumoral TIM3-positive (TIM3^{+}, shown in C) or PD1-positive (PD1^{+}, shown in D) CD8^{+} T cell populations in TS/A-Control or TS/A-RACK7-KD tumors, with or without tamoxifen treatment. **E** and **F.** Abundance of intratumoral CEACAM1-positive (represented by the CD66-positive subset, shown in E) and PD-L1-positive (represented by the CD274-positive subset, shown in F) CD45-negative (CD45^{-}) non-immune cells in TS/A-Control or TS/A-RACK7-KD tumors, with or without tamoxifen treatment. **G.** Abundance of intratumoral myeloid cell populations in TS/A-Control or TS/A-RACK7-KD tumors, with or without tamoxifen treatment. **H, I, J, K,** and **L.** Abundance of intratumoral CD11c^{+} (shown in H), CD11c^{+}CD86^{+} (shown in I), CD11c^{+}CD103^{+} (shown in J), CD11c^{+}MHCII^{+}CD103^{-} (shown in K), and CD11c^{+}MHCII^{+}CD103^{+} (shown in L) populations in TS/A-Control or TS/A-RACK7-KD tumors, with or without tamoxifen treatment. Data from (A) to (L) are presented as bar plots of % indicated cell populations, n=6, p-values are calculated using one-way ANOVA followed by Tukey post-hoc test.

Figure S9 (related to Figure 4)

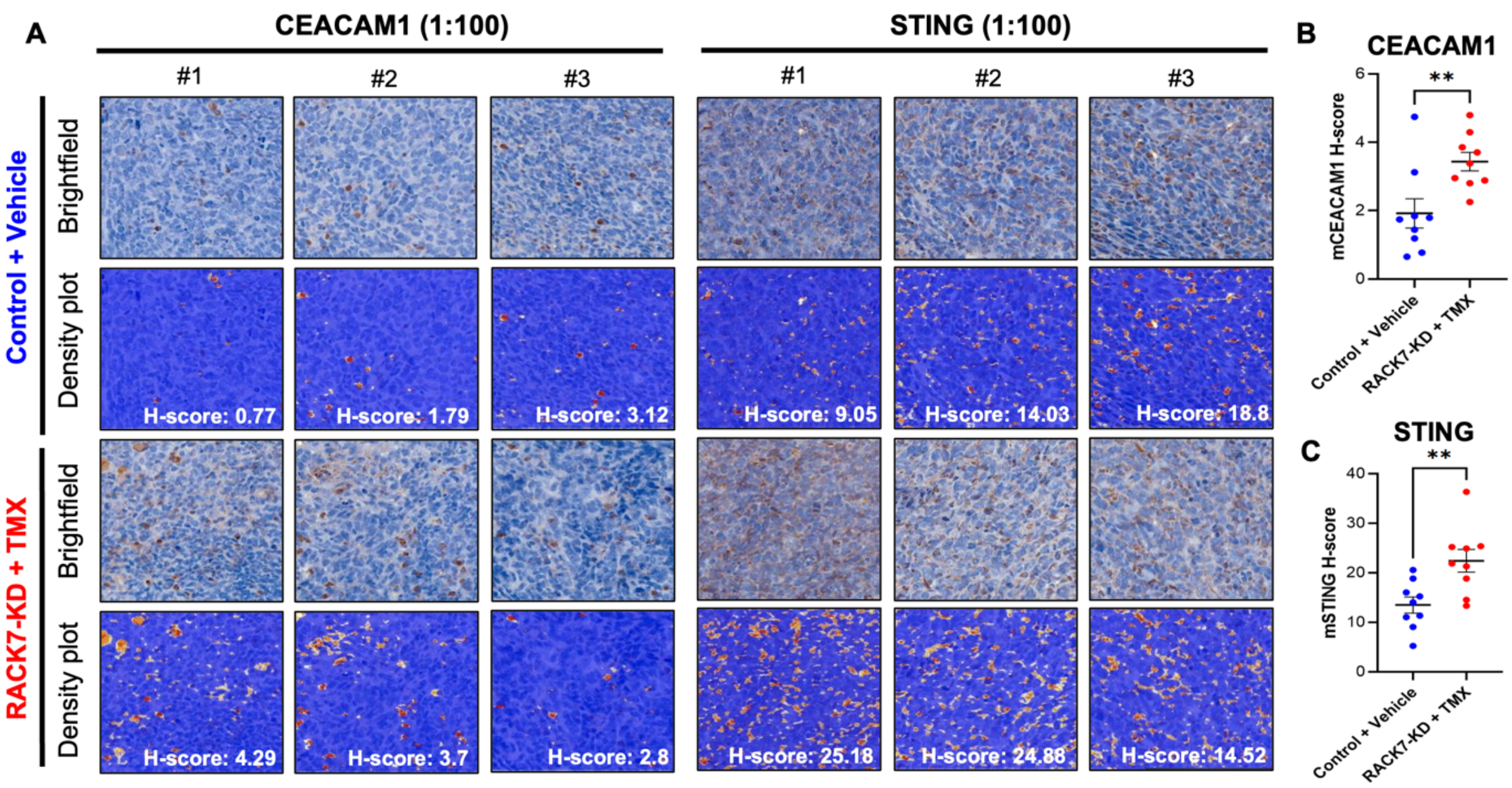

Figure S9 (related to Figure 4). The combined treatment of tamoxifen and RACK7 knockdown enhance CEACAM1 and STING expression in vivo.

A. Representative immunohistochemistry (IHC) density plots of CEACAM1 and STING staining in 4T1 tumors, with or without combined treatment of RACK7-KD and tamoxifen. **B** and **C.** Quantification results of CEACAM1 (B) and STING (C) staining in 4T1 tumors, with or without combined treatment of RACK7-KD and tamoxifen. Data is presented as mean H-score ± SEM, **p< 0.01.

Figure S10 (related to Figure 4)

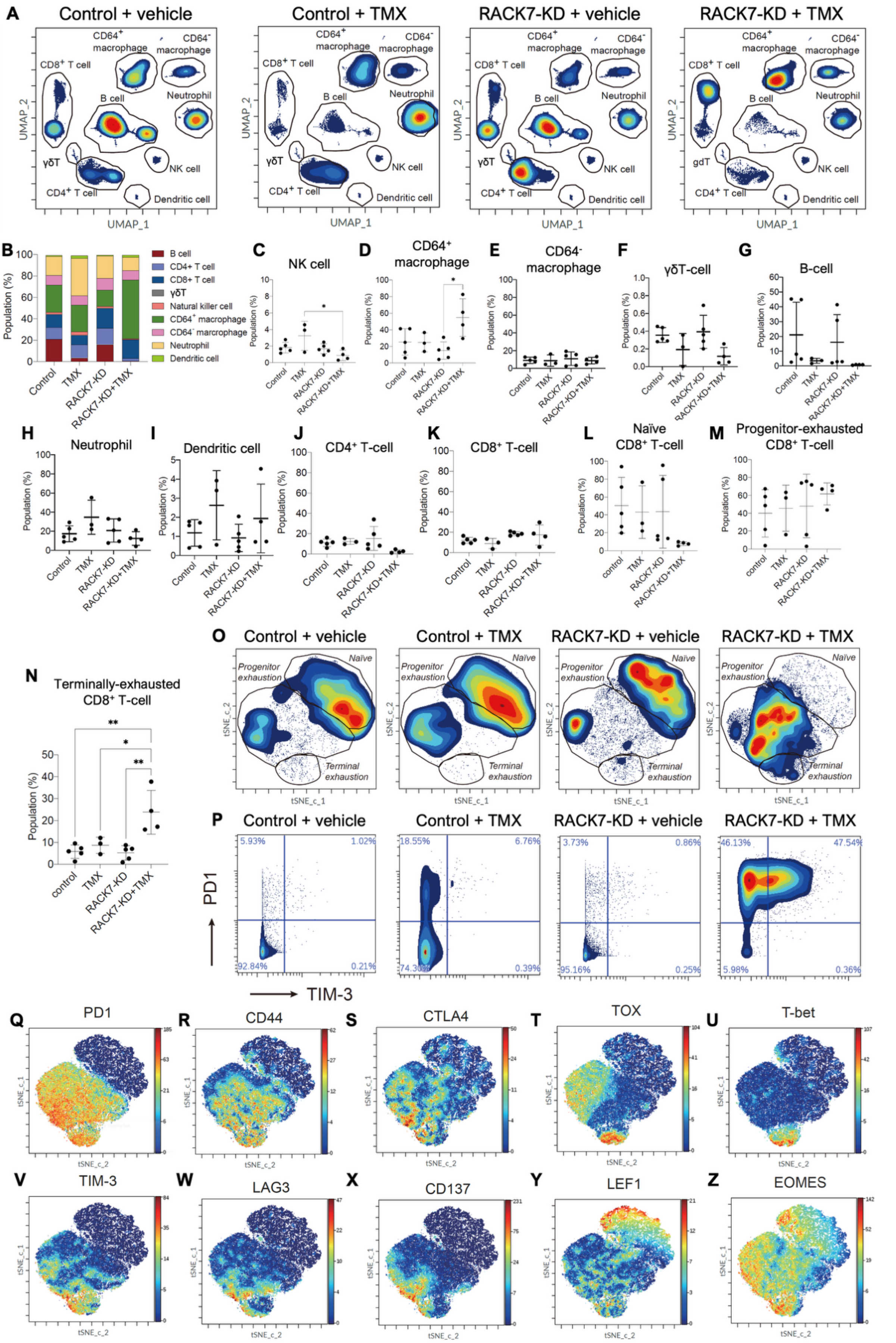

Figure S10 (related to Figure 4). The combined treatment of tamoxifen and RACK7 knockdown reshaped the immune tumor landscape and promotes the growth of ER$^-$ 4T1 tumors in vivo.

A and **B.** UMAP plots (A) and quantification data (B) indicating the percentages of distinct immune cell populations in 4T1-Control or 4T1-RACK7-KD tumors, with or without tamoxifen treatment. **C - I.** Abundance of intratumoral NK cells (C), CD64$^+$ macrophages (D), CD64$^-$ macrophages (E), γδ T-cells (F), B-cells (G), neutrophils (H), and dendritic cells (I) in 4T1-Control or 4T1-RACK7-KD tumors, with or without tamoxifen treatment. **J – N.** Abundance of intratumoral CD4$^+$ (J), CD8$^+$ (K), Naïve CD8$^+$ (L), progenitor-exhausted (M), and terminally-exhausted (N) CD8$^+$ T-cells in 4T1-Control or 4T1-RACK7-KD tumors, with or without tamoxifen treatment. **O.** tsNE plots indicating the percentages of naïve, progenitor-exhausted, and terminally-exhausted CD8$^+$ T-cells in 4T1-Control or 4T1-RACK7-KD tumors, with or without tamoxifen treatment. **P.** Density plots indicating the distribution of double negative (TIM3$^-$PD1$^-$), single-TIM3-positive (TIM3$^+$PD1$^-$), single-PD1-positive (TIM3$^-$PD1$^+$), and double-positive (TIM3$^+$PD1$^+$) population of terminally-exhausted CD8$^+$ T-cells in 4T1-Control or 4T1-RACK7-KD tumors, with or without tamoxifen treatment. **Q - Z.** UMAP plots indicating the intensity of the T-cell markers PD1 (Q), CD44 (R), CTLA4 (S), TOX (T), T-bet (U), TIM3 (V), LAG3 (W), CD137 (X), LEF1 (Y), and EOMES (Z) among CD8$^+$ T-cells in 4T1-Control or 4T1-RACK7-KD tumors, with or without tamoxifen treatment. Data from (C) to (N) and (Q) to (Z) are presented as distribution of the indicated cell populations, n=4, p-values are calculated using one-way ANOVA followed by Tukey post-hoc test, *$p<0.05$, **$p<0.005$. γδ T-cells, gamma-delta T-cells; NK cells, natural killer cells.

Figure S11 (related to Figure 4)

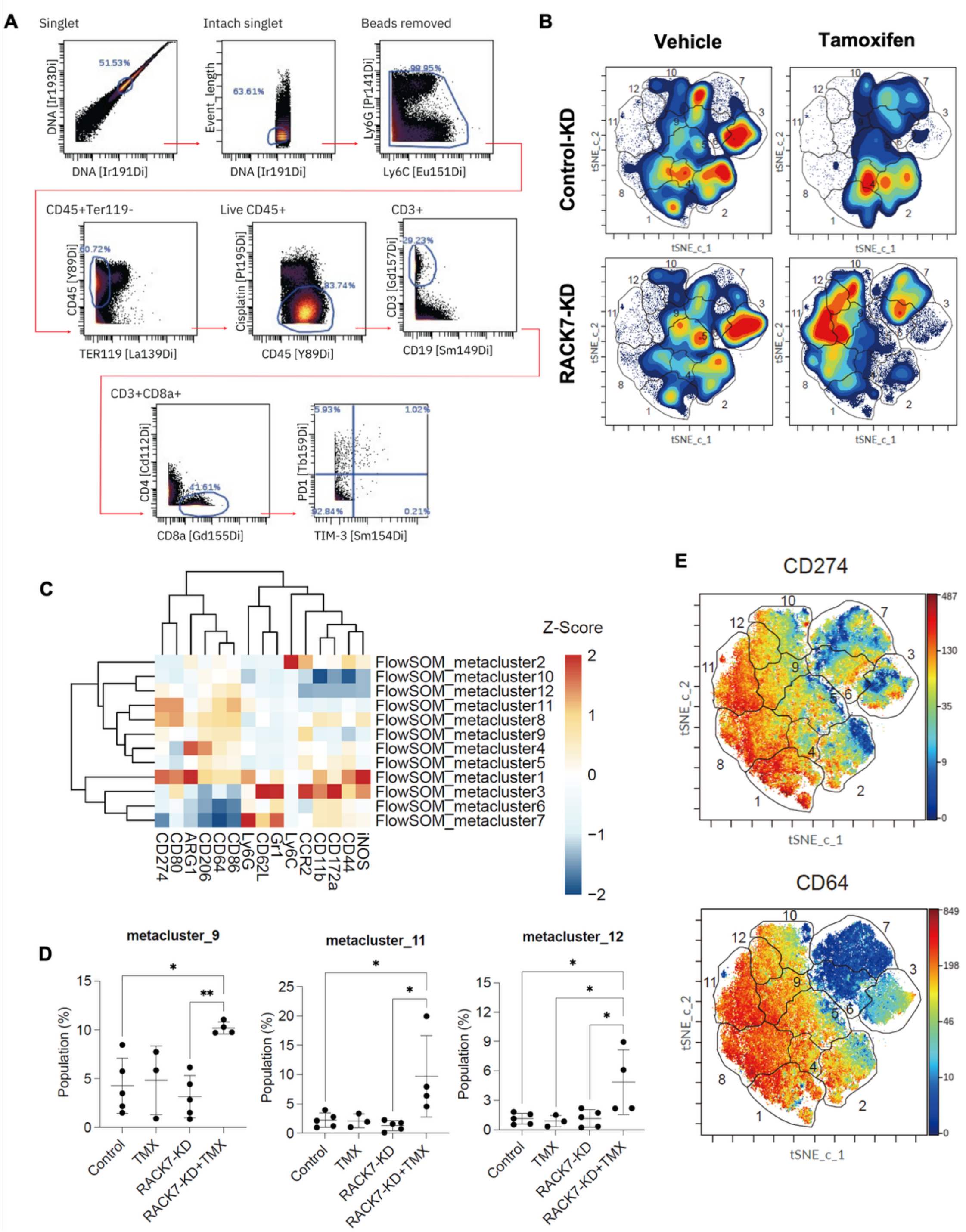

Figure S11 (related to Figure 4). The combined treatment of tamoxifen and RACK7 knockdown induce CD8$^+$ T-cell exhaustion and shifts in F4/80$^+$ macrophage populations in 4T1 xenografts.
A. CyTOF gating strategy for CD3$^+$CD8a$^+$ T-cells from CD45$^+$ immune cell populations. **B.** Dimensionality reduction analysis and FlowSOM clustering of F4/80$^+$ macrophages identify 12 distinct macrophage clusters in 4T1-Control or 4T1-RACK7-KD tumors, with or without tamoxifen treatment. **C.** Marker expression patterns for the 12 macrophage clusters. **D.** Abundance of macrophages in metaclusters 9, 11, and 12 in 4T1-Control or 4T1-RACK7-KD tumors, with or without tamoxifen treatment. Data is presented as mean ± SD, *$p < 0.05$. **E.** Heatmap of CD274 (top) and CD64 (bottom) expression across the 12 macrophage clusters.

Figure S12 (related to Figure 5)

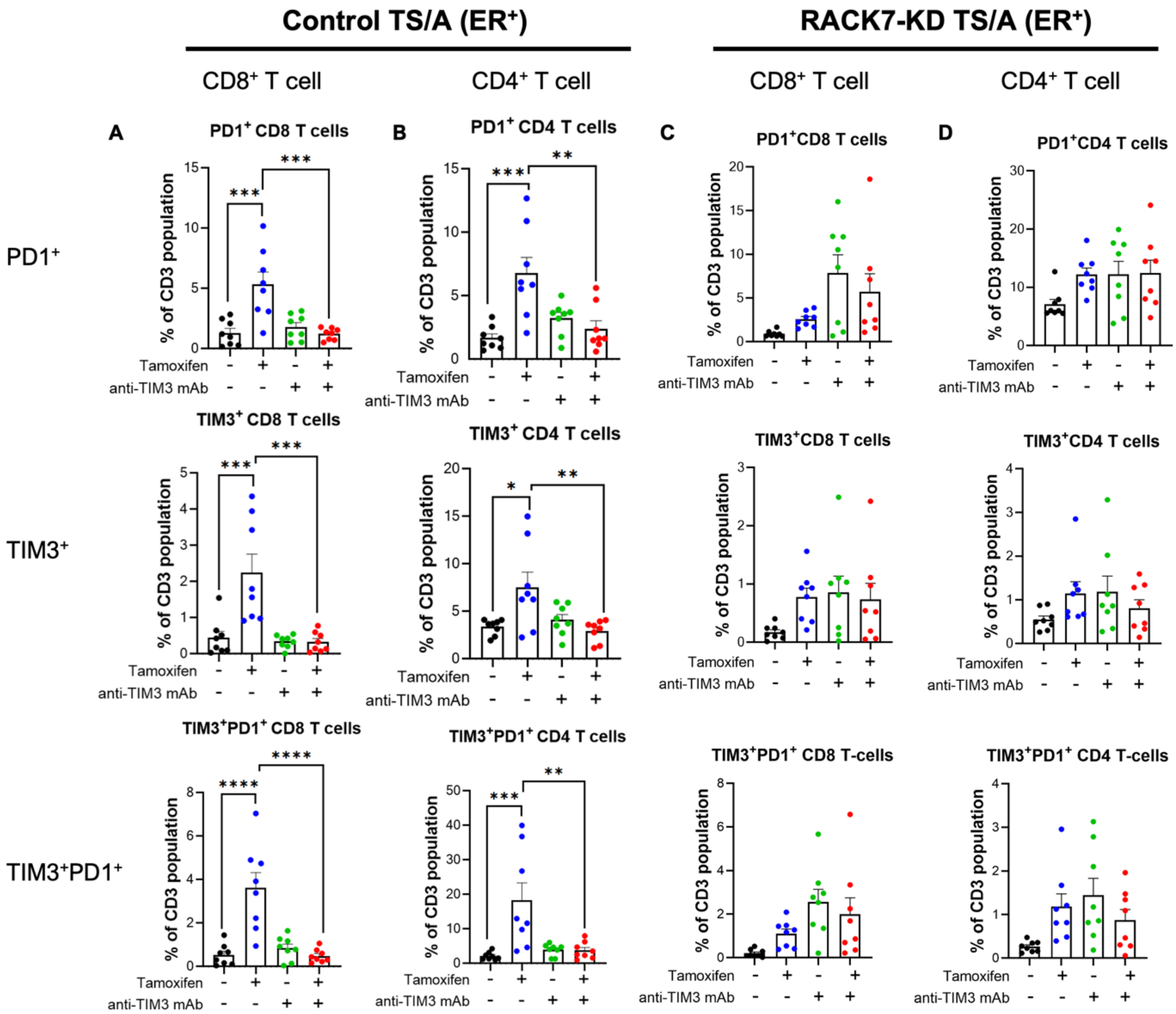

Figure S12 (related to Figure 5). Combination of tamoxifen and anti-TIM3 mAb reshapes T-cell exhaustion profiles of TS/A tumors in vivo.
A and **B.** Abundance of intratumoral PD1$^+$, TIM3$^+$, and TIM3$^+$PD1$^+$ CD8$^+$ (A) and CD4$^+$ (B) T cell populations in Control-TS/A tumors, with or without tamoxifen and anti-TIM3 monoclonal antibody monotherapy or combination therapy. **C** and **D.** Abundance of intratumoral PD1$^+$, TIM3$^+$, and TIM3$^+$PD1$^+$ CD8$^+$ (C) and CD4$^+$ (D) T cell populations in RACK7-KD-TS/A tumors, with or without tamoxifen and anti-TIM3 monoclonal antibody monotherapy or combination therapy.

Figure S13 (related to Figure 5)

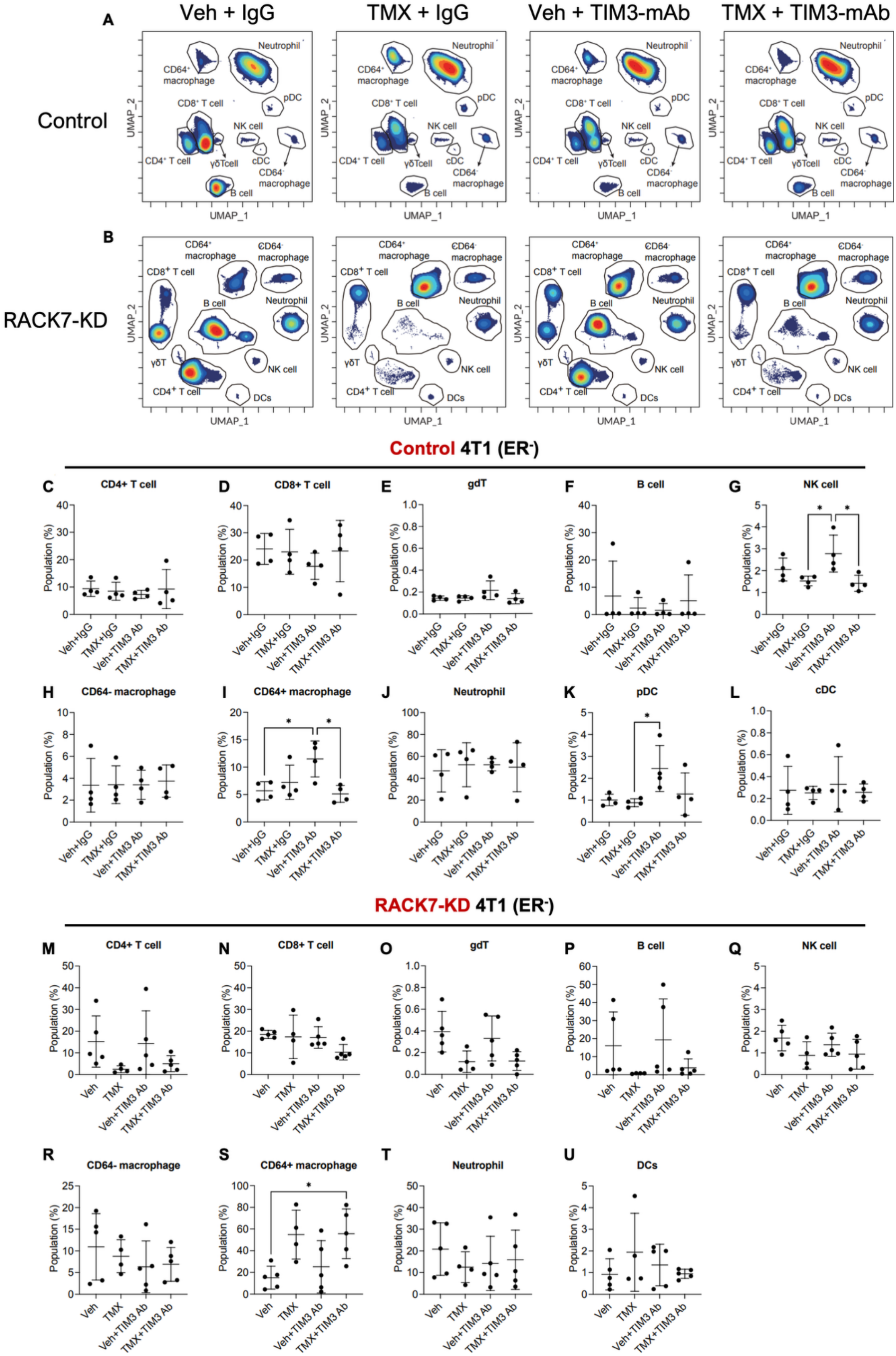

Figure S13 (related to Figure 5). Combination of tamoxifen and anti-TIM3 mAb reshapes the tumor-immune microenvironment of 4T1 tumors in vivo.

A and **B.** UMAP plots indicating the percentages of distinct immune cell populations in 4T1-Control (A) or 4T1-RACK7-KD tumors (B), with or without tamoxifen and anti-TIM3 monoclonal antibody monotherapy or combination therapy. **C – L.** Abundance of intratumoral $CD4^+$ T cells (C), $CD8^+$ T cells (D), gamma-delta T cells (E), B cells (F), NK cells (G), $CD64^-$ macrophages (H), $CD64^+$ macrophages (I), Neutrophil (J), plasmacytoid dendritic cells (K), and conventional dendritic cells (L) in Control-4T1 tumors, with or without tamoxifen and anti-TIM3 monoclonal antibody monotherapy or combination therapy. **M – U.** Abundance of intratumoral $CD4^+$ T cells (M), $CD8^+$ T cells (N), gamma-delta T cells (O), B cells (P), NK cells (Q), $CD64^-$ macrophages (R), $CD64^+$ macrophages (S), neutrophil (T), and dendritic cells (U) in RACK7-KD-4T1 tumors, with or without tamoxifen and anti-TIM3 monoclonal antibody monotherapy or combination therapy.

Figure S14 (related to Figure 5)

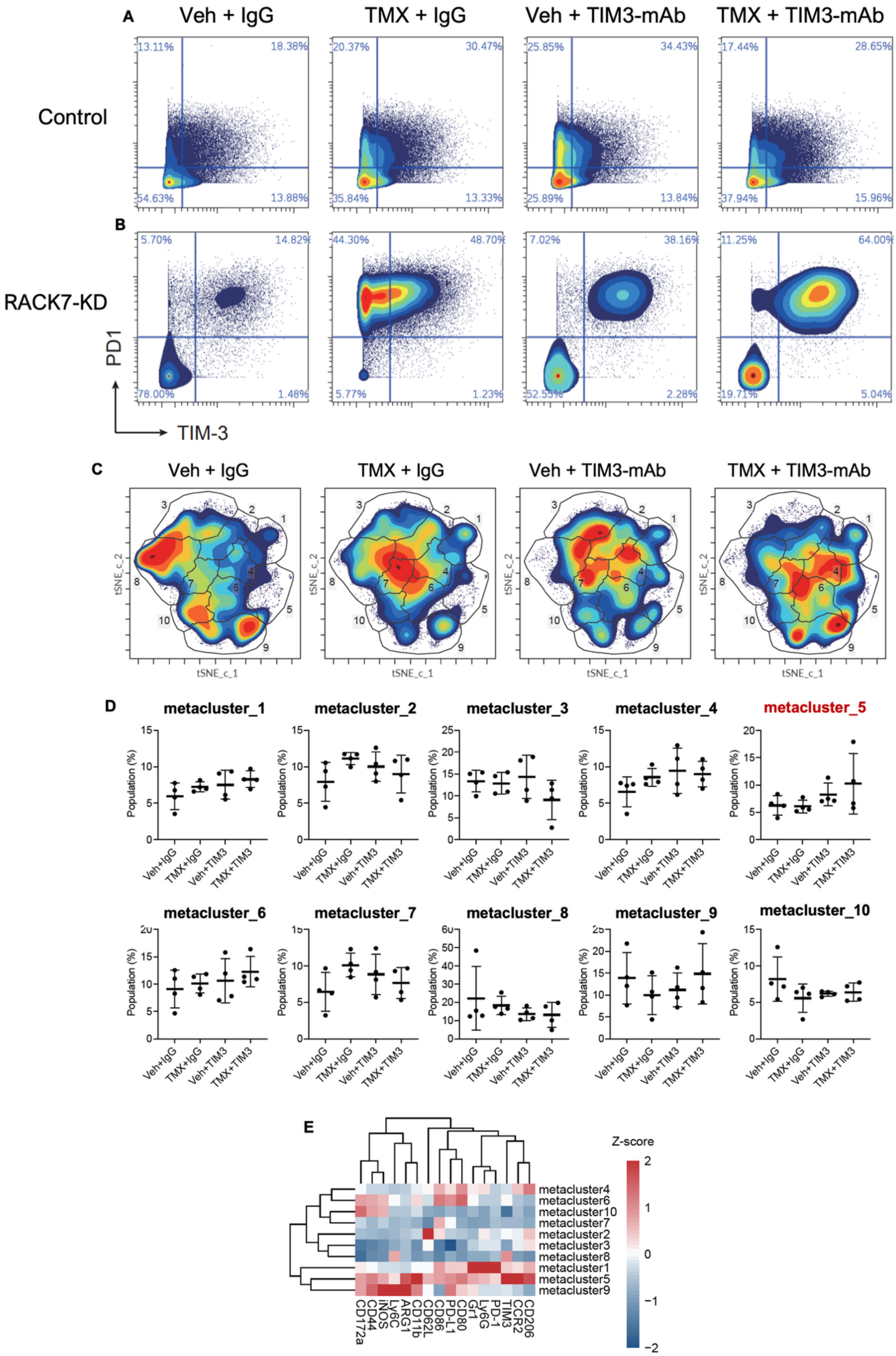

Figure S14 (related to Figure 5). Combination of tamoxifen and anti-TIM3 mAb reshapes the tumor-immune microenvironment of $CD8^+$ T cell and $CD64^+$ macrophage population in 4T1 tumors in vivo.

A and **B.** Density plots indicating the distribution of double negative ($TIM3^-PD1^-$), single-TIM3-positive ($TIM3^+PD1^-$), single-PD1-positive ($TIM3^-PD1^+$), and double-positive ($TIM3^+PD1^+$) $CD8^+$ T cells in Control-4T1 (A) and RACK7-KD-4T1 (B) tumors, with or without tamoxifen and anti-TIM3 monoclonal antibody monotherapy or combination therapy. **C.** Dimensionality reduction analysis and FlowSOM clustering of $F4/80^+$ macrophages identify 10 distinct macrophage clusters in Control-4T1 tumors, with or without tamoxifen and anti-TIM3 monoclonal antibody monotherapy or combination therapy. **D.** Intratumoral abundance of macrophages in metaclusters 1-10 in Control-4T1 tumors, with or without tamoxifen and anti-TIM3 monoclonal antibody monotherapy or combination therapy. **E**. Marker expression patterns for the 10 macrophage clusters.

Figure S15 (related to Discussion)

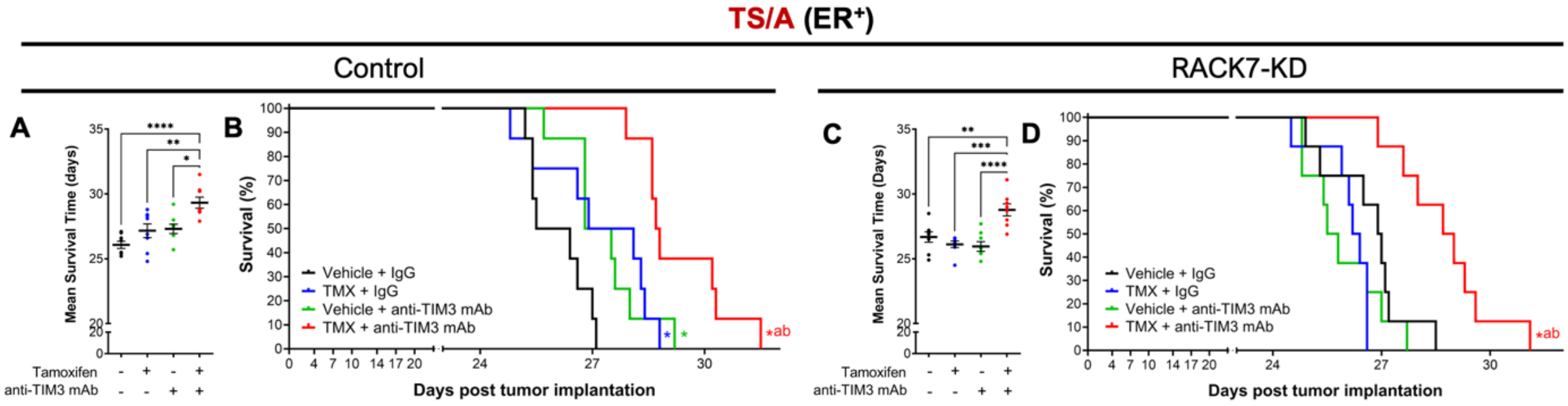

Figure S15 (related to Discussion). Surrogate survival analysis demonstrates therapeutic efficacy of combined TMX + TIM3-mAb therapy in TS/A tumors in vivo.
A and **B.** Mean survival time (A) and surrogate survival plots (B) of Control-4T1 tumors with or without tamoxifen and anti-TIM3 monoclonal antibody monotherapy or combination therapy. **C** and **D.** Mean survival time (C) and surrogate survival plots (D) of RACK7-KD-4T1 tumors with or without tamoxifen and anti-TIM3 monoclonal antibody monotherapy or combination therapy. Survival time is defined as estimated number of days for individual mice to reach tumor volumes equal to 800 mm^3.
