# Supplementary material for: Dual Role of Tamoxifen in Enhancing STING and CEACAM1 Expression to Prime a Favorable Tumor Microenvironment for Anti‐TIM3 Immunotherapy
Source: Adv Sci (Weinh). 2026 Aug 17:e03303. Online ahead of print. doi: 10.1002/advs.202503303 (PMC13479595; doi:10.1002/advs.202503303)
Supplement: Supplementary file 2 [file ADVS-9999-e03303-s002.pdf]

Supplemental Table S1 (key resources table)

REAGENT or RESOURCE	SOURCE	IDENTIFIER
**Antibodies**		
Donkey polyclonal, anti-rabbit AF488-conjugated anti-IgG	Invitrogen	Cat# A-21206, RRID:AB_2535792
Mouse monoclonal, APC-conjugated anti-human-CD25	Biolegend	Cat# 302609, RRID:AB_314280
Mouse monoclonal, APC-conjugated anti-mouse-CD3	Biolegend	Cat# 100268, RRID: AB_2876392
Mouse monoclonal, APC-conjugated anti-mouse-CD66a (aka CEACAM1)	Biolegend	Cat# 134509, RRID:AB_2632612
Mouse monoclonal, FITC-conjugated anti-human-CD69	Biolegend	Cat# 310903, RRID:AB_314838
Mouse monoclonal, HRP-conjugated anti-human-β-actin	GenScript	Cat# A00730, RRID:AB_914100
Mouse monoclonal, PE-conjugated anti-H-2Kb	BD Biosciences	Cat# 553570, RRID: AB_394928
Mouse monoclonal, PE-Cy7-conjugated anti-human PD-L1	Biolegend	Cat# 374505, RRID: AB_2734433
Mouse monoclonal, PE-Cy7-conjugated anti-mouse-CD279 (PD1)	BD Biosciences	Cat# 561272, RRID: AB_10611585
Rabbit monoclonal, anti-human-CEACAM1	Abcam	Cat# ab300061, RRID:AB_3096353
Rabbit monoclonal, anti-human-IRF3	Cell Signaling Technology	Cat# 11904, RRID:AB_2722521
Rabbit monoclonal, anti-human-phospho-IRF3 (Ser396)	Cell Signaling Technology	Cat# 29047, RRID:AB_2773013
Rabbit monoclonal, anti-human-phospho-STAT1 (Tyr701)	Cell Signaling Technology	Cat# 7649, RRID:AB_10950970
Rabbit monoclonal, anti-human-phospho-STAT3 (Tyr705)	Cell Signaling Technology	Cat# 9131, RRID:AB_331586
Rabbit monoclonal, anti-human-phospho-TBK1 (Ser172)	Cell Signaling Technology	Cat# 5483, RRID:AB_10693472
Rabbit monoclonal, anti-human-STAT1	Cell Signaling Technology	Cat# 14994, RRID:AB_2737027
Rabbit monoclonal, anti-human-STAT3	Cell Signaling Technology	Cat# 12640, RRID:AB_2629499
Rabbit monoclonal, anti-human-STING	Cell Signaling Technology	Cat# 13647, RRID:AB_2732796
Rabbit monoclonal, anti-human-TBK1	Cell Signaling Technology	Cat# 3504, RRID:AB_2255663
Rabbit polyclonal, anti-KDM5C (aka JARID1C)	Bethyl Laboratories	Cat# A301-034A, RRID:AB_817995
Rabbit polyclonal, anti-RACK7 (aka PRKCBP1)	Bethyl Laboratories	Cat# A302-090A, RRID:AB_1604208
Rat anti-mouse CD16/32 (Mouse BD Fc Block)	BD Biosciences	Cat# 553142, RRID: AB_394656
Rat IgG2a isotype control, anti-trinitrophenol	BioXCell	Cat# BE0089, RRID: AB_1107769
Rat monoclonal anti-HA	Roche	Cat# 11867431001, RRID:AB_390919
Rat monoclonal, anti-mouse-CD366 (aka TIM3)	BioXCell	Cat# BE0115, RRID: AB_10949464
Rat monoclonal, BUV-737-conjugated anti-mouse-CD274 (aka PD-L1)	BD Biosciences	Cat# 741877, RRID:AB_2871203
Rat monoclonal, eFluor450-conjugated anti-mouse-CD8a	eBioscience	Cat# 48-0081-82; RRID: AB_1272198
Rat monoclonal, FITC-conjugated anti-MHCII (I-A/I-E)	eBioscience	Cat# 11-5321-81, RRID: AB_465232
Rat monoclonal, PE-conjugated anti-human-CD44	Biolegend	Cat# 103023, RRID:AB_312959
Rat monoclonal, PE-conjugated anti-mouse-CD4	Biolegend	Cat# 100482, RRID: AB_2860585

Rat monoclonal, PerCP-eF710-conjugated anti-mouse-CD366 (TIM3)	eBioscience	Cat# 46-5870-82, RRID: AB_2802279
Rat monoclocnal, AlexaFluor700-conjugated anti-mouse-CD45	Biolegend	Cat# 103128, RRID: AB_493715
**Experimental models: Cell lines**		
Human: MCF7	Li Laboratory	
Human: T47D	Li Laboratory	
Human: MDA-MB-157	Li Laboratory	
Human: vehicle-control-MCF7 (DMSO)	Shen Laboratory	
Human: cTMX-MCF7-clone 1	Shen Laboratory	
Human: cTMX-MCF7-clone 2	Shen Laboratory	
Human: vehicle-control-T47D (DMSO)	Shen Laboratory	
Human: cTMX-T47D-clone 1	Shen Laboratory	
Human: cTMX-T47D-clone 2	Shen Laboratory	
Human: HEK293T	Roeder Laboratory	
Mouse: 4T1	Li Laboratory	
Mouse: TS/A	Guha Laboratory	
Human: shControl-T47D	This study	
Human: shRACK7-T47D	This study	
Human: shControl-MDA-MB-157	This study	
Human: shRACK7-MDA-MB-157	This study	
Mouse: shControl-TS/A	This study	
Mouse: shRACK7-TS/A	This study	
Mouse: shControl-4T1	This study	
Mouse: shRACK7-4T1	This study	
**Oligonucleotides**		
qPCR primers	This study	see Table S2
shRNA sequences	This study	see Table S2
**Cell culture media and supplements**		
RPMI-1640 (phenol-red free)	Sigma-Aldrich	Cat# R8755
DMEM/F-12, powder, HEPES	Gibco	Cat# 12400024
DMEM, powder, high glucose, pyruvate	Gibco	Cat# 12800017
Fetal bovine serum (FBS)	Gibco	Cat# 26140079
Penicillin G sodium salt	Sigma-Aldrich	Cat# P3032
Streptomycin sulfate salt	Sigma-Aldrich	Cat# S9137

Immunocult human CD3/CD28 T-cell Activator	Stem Cell Technologies	Cat# 10971
Human Recombinant IL-2	Stem Cell Technologies	Cat# 78036
**Chemicals, peptides, and recombinant proteins**		
Bovine Serum Albumin (flow cytometry)	BioWorld	Cat# 22070004-1
Bovine Serum Albumin (immunoblotting)	BioBasic	Cat# D0024
Cisplatin	Fluidigm	Cat# 201064
CD3 MicroBeads (human T-cell isolation)	Miltenyi Biotec	Cat# 130-097-043
Collagenase Type I	Gibco	Cat# 17100-017
Collagenase Type IV	Gibco	Cat# 17104-019
cOmplete EDTA-free Protease Inhibitor Cocktail	Roche	Cat# 04693132001
Cycloheximide	Sigma-Aldrich	Cat# C7698
Dimethyl Sulfoxide (DMSO)	Sigma-Aldrich	Cat# D5879
DNAse I, grade II, from bovine pancreas	Roche Diagnostics GmBH	Cat# 10104159001
Doxycycline hyclate	Sigma-Aldrich	Cat# D9891
Estradiol (E_2)	Selleckchem	Cat# S1709
Ficoll-Paque PLUS media	Cytiva	Cat# 17144003
H-151 (STING inhibitor)	Chen Laboratory	https://doi.org/10.1016/j.bmc.2023.117502
Olive Oil	Sigma Aldrich	Cat# 01514
PhosSTOP (Phosphatase Inhibitor Cocktail)	Roche	Cat# 04906837001
Propidium iodide	Sigma-Aldrich	Cat# P4170
Puromycin dihydrochloride	Gibco	Cat# A1113803
RBC lysis buffer (10X)	Biolegend	Cat# 420301
Tamoxifen (in vitro)	Selleckchem	Cat# S1238
Tamoxifen (in vivo)	Sigma Aldrich	Cat# T5648
Trident Femto Western HRP Substrate	GeneTex	GTX14698
Trident Plus Western HRP Substrate	GeneTex	GTX400006
UltraComp eBeads Compensation Beads	Invitrogen	Cat# 01-2222-42
**Commercial assays**		
Cell-ID 20-Plex Pd Barcoding kit	Fluidigm	Cat# 201060
Endotoxin Free Midi plasmid purification kit	GeneAid	Cat# PIE25
Lipofectamine 3000 Transfection Kit	Thermo Fisher Scientific	Cat# L3000015
Mycoplasma PCR detection kit	ABM	Cat# G238
Pierce BCA Protein Assay kit	Thermo Fisher Scientific	Cat# 23325

Power SYBR Green PCR Master Mix	Thermo Fisher Scientific	Cat# 4368708
Presto Mini plasmid purification kit	GeneAid	Cat# PDH300
PrimeScript RT Master Mix	Takara	Cat# RR036A
RNASpin Mini Kit	Sigma-Aldrich	Cat# GE25050071
Tag-it Violet Proliferation and Cell tracking Dye kit	Biolegend	Cat# 425101
Zombie NIR Fixable Viability kit	Biolegend	Cat# 77184
**Recombinant DNA**		
GFP-RACK7	Addgene	RRID:Addgene_65401
pCDNA3.1(+) myc-3X-HA-RXR	Roeder Laboratory	http://dx.doi.org/10.1016/j.molcel.2017.06.028
pLVX-mCherry (DOX-inducible tet-on)	Addgene	RRID:Addgene_180646
tet-on-HA-RACK7-OE	This study	
**Software and algorithms**		
Prism 9.5.0	GraphPad	www.graphpad.com
GSEA	Mesirov Laboratory	https://doi.org/10.1073/pnas.0506580102
BioJupies	Ma'ayan Laboratory	https://doi.org/10.1016/j.cels.2018.10.007
cBioPortal	Schultz Laboratory	https://doi.org/10.1126/scisignal.2004088
Fiji (ImageJ)	Cardona Laboratory	https://doi.org/10.1038/nmeth.2019
TNMplot	Gyorffy Laboratory	https://doi.org/10.3390/ijms22052622
WebGestalt	Zhang Laboratory	https://doi.org/10.1093/nar/gkz401
**Deposited data**		
RNA-Seq	PRJNA1175753	https://dataview.ncbi.nlm.nih.gov/object/PRJNA1175753?reviewer=kbbvtqgkipba2pv1en2svu158q
