# Supplementary material for: Dual Role of Tamoxifen in Enhancing STING and CEACAM1 Expression to Prime a Favorable Tumor Microenvironment for Anti‐TIM3 Immunotherapy
Source: Adv Sci (Weinh). 2026 Aug 17:e03303. Online ahead of print. doi: 10.1002/advs.202503303 (PMC13479595; doi:10.1002/advs.202503303)
Supplement: Supplementary file 3 [file ADVS-9999-e03303-s003.pdf]

Supplemental Table S3

**4T1 CyTOF panel**					
**Target**	**Metal**	**Element**	**Clone**	**Source**	**Identifier (Cat #)**
BC1	102	Pd			
BC2	104	Pd			
BC3	105	Pd			
BC4	106	Pd			
BC5	108	Pd			
BC6	110	Pd			
CD45	89	Y	30-F11	Biolegend	103102
B220	111	Cd	RA3-6B2	Biolegend	103202
CD4	112	Cd	RM4-5	Biolegend	100506
CCR2	113	In	QA18A56	Biolegend	160102
Gr1	114	Cd	RB6-8C5	Biolegend	108402
TCRb	115	In	H57-597	Biolegend	109254
CD44	116	Cd	IM7	BD	553131
TER119	139	La	Ter-119	Biolegend	116202
CD11b	140	Ce	M1/70	Biolegend	101202
Ly6G	141	Pr	1A8	Fluidigm	3141008B
CD47	142	Nd	miap301	BD	555297
CD11c	143	Nd	HL3	BD	553799
XCR1	144	Nd	ZET	Biolegend	148202
CD172a	145	Nd	P84	Biolegend	144004
iNOS	146	Nd	CXNFT	eBioscience	14-5920-82
CD127	147	Sm	A7R34	Biolegend	135002
CD103	148	Nd	2E7	Biolegend	121402
CD19	149	Sm	6D5	Biolegend	115502
PDCA1	150	Nd	120G8.04	Dendritics	DDX0390P-100
Ly6C	151	Eu	HK1.4	Biolegend	128002
TOX	152	Sm	TXRX10	eBioscience	14-6502-82
CD274 (PD-L1)	153	Eu	10F.9G2	Biolegend	124302
TIM3	154	Sm	B8.2C12	Biolegend	134002
CD8a	155	Gd	53-6.7	Biolegend	100702
EOMES	156	Gd	Dan11mag	eBioscience	14-4875-82
CD3	157	Gd	17A2	Biolegend	100202
Foxp3	158	Gd	FJK-16s	eBioscience	14-5773-82
PD-1	159	Tb	29F.1A12	Fluidigm	3159024B
CD206	160	Gd	C068C2	Biolegend	141702
T-bet	161	Dy	O4-46	BD	561263
TCRgd	162	Dy	GL3	Biolegend	118101
CD80	163	Dy	16-10A1	Biolegend	104735
CD62L	164	Dy	MEL-14	Biolegend	104402
NK1.1	165	Ho	PK136	Biolegend	108702
ARG1	166	Er	poly-Rb	NOVUS	NBP1-32731
NKp46	167	Er	29A1.4	Biolegend	137602
CD25	168	Er	PC61	Biolegend	102002
F4/80	169	Tm	BM8	Biolegend	123103

CD137 (41BB)	170	Er	17B5	Biolegend	106114
CD64	171	Yb	X54-5/7.1	Biolegend	139301
CD86	172	Yb	GL-1	Biolegend	105002
CTLA4 (CD152)	173	Yb	UC10-4B9	Biolegend	106302
LAG3 (CD223)	174	Yb	C9B7W	Biolegend	125202
LEF1	176	Yb	EPR2029Y	abcam	ab215999
I-A/I-E (MHCII)	209	Bi	M5/114.15.2	Biolegend	107602
Cell-ID	191	Ir		Fluidigm	201192B
Cell-ID	193	Ir		Fluidigm	201192B
Cisplatin	198	Pt		Sigma-Aldrich	P4394
**T47D CyTOF panel**					
**Target**	**Metal**	**Element**	**Clone**	**Source**	**Identifier (Cat #)**
BC1	102	Pd			
BC2	104	Pd			
BC3	105	Pd			
BC4	106	Pd			
BC5	108	Pd			
BC6	110	Pd			
CD45	89	Y	30-F11	Biolegend	103102
B220	111	Cd	RA3-6B2	Biolegend	103202
CD4	112	Cd	RM4-5	Biolegend	100506
CCR2	113	In	QA18A56	Biolegend	160102
Gr1	114	Cd	RB6-8C5	Biolegend	108402
Vimentin	115	In	D21H3	CST	46173
CD44	116	Cd	IM7	BD	553131
TER119	139	La	Ter-119	Biolegend	116202
CD11b	140	Ce	101202	Biolegend	20210415
Ly6G	141	Pr	1A8	Fluidigm	3141008B
CD47	142	Nd	miap301	BD	555297
CD11c	143	Nd	HL3	BD	553799
XCR1	144	Nd	ZET	Biolegend	148202
CD172a	145	Nd	P84	Biolegend	144002
iNOS	146	Nd	CXNFT	eBioscience	14-5920-82
CD127	147	Sm	A7R34		04-Oct-17 LC
CD103	148	Nd	2E7	Biolegend	121402
CD19	149	Sm	6D5	Biolegend	115502
PDCA1	150	Nd	120G8.04	Dendritics	DDX0390P-100
Ly6C	151	Eu	HK1.4	Biolegend	128002
TOX	152	Sm	TXRX10	eBioscience	14-6502-82
CD274 (PD-L1)	153	Eu	10F.9G2	Fluidigm	3153016B
TIM3	154	Sm	RMT3-23	Biolegend	119702
alpha-SMA	155	Gd	1A4/asm-1	NOVUS	NBP2-34522
CD8a	156	Gd	53-6.7	BD	553027
CD3	157	Gd	17A2	Biolegend	100202
Foxp3	158	Gd	FJK-16s	eBioscience	14-5773-82
PD-1	159	Tb	29F.1A12	Biolegend	135202
CD206	160	Gd	C068C2	Biolegend	141702

T-bet	161	Dy	O4-46	BD	561263
EOMES	162	Dy	Dan11mag	eBioscience	14-4875-82
STING	163	Dy	SP339	Abcam	ab238796
CD62L	164	Dy	MEL-14	Biolegend	104402
p-IRF3	165	Ho	D601M	CST	61166
ARG1	166	Er	poly-Rb	NOVUS	NBP1-32731
CX3CR1	167	Er	SA011F11	Biolegend	149002
p-STING	169	Tm	EPR29040-93	Abcam	ab324230
p-TBK1	170	Er	D52C2	CST	54382
CD64	171	Yb	X54-5/7.1	Biolegend	139301
CD86	172	Yb	GL-1	Biolegend	105002
ER	173	Yb	6A6E8	NOVUS	NBP2-61764
LAG-3	174	Yb	C9B7W	Biolegend	125202
NKp46	175	Lu	29A1.4	Biolegend	137602
F4/80	176	Yb	T45-2342	BD	565409
I-A/I-E (MHCII)	209	Bi	M5/114.15.2	Biolegend	107602
DNA	191/193	Ir			
Cisplatin Viability	195	Pt			
