# Supplementary material for: Dual Role of Tamoxifen in Enhancing STING and CEACAM1 Expression to Prime a Favorable Tumor Microenvironment for Anti‐TIM3 Immunotherapy
Source: Adv Sci (Weinh). 2026 Aug 17:e03303. Online ahead of print. doi: 10.1002/advs.202503303 (PMC13479595; doi:10.1002/advs.202503303)
Supplement: Supplementary file 4 [file ADVS-9999-e03303-s004.pdf]

Supplemental Table S2

REAGENT or RESOURCE			SOURCE and IDENTIFIER	
**Target**	**Forward (5' to 3')**	**Reverse (3' to 5')**	**Source**	**Identifier**
**Oligonucleotides (mrRNA RT-qPCR)**				
human IFIT1	ATTTACAGCAACCATGAGTACAAA	GGCTTCCTCATTCTGGCCTT	this study	
human IFIT2	AGAACGCCATTGACCCTCTG	TAAAACTTGGCTGCACTGCG	this study	
human IFIT3	AACTGCAGGGAAACAGCCAT	GGCATTTCAGCTGTGGAAGG	this study	
human OAS1	TTGACTGGCGGCTATAAACC	GAGCTCCAGGGCATACTGAG	this study	
human OAS2	TCAGCGAGGCCAGTAATCTT	TTGTTGGGCTGGAGAAACTC	this study	
human OASL	TTTCTGAGGCAGGAGCATTT	TTGCCACATGGTTTTCCATA	this study	
human RSAD2	CTGAGAGGGCCAGATGAGAC	TGGCTCTCCACCTGAAAAGT	this study	
human CCL5	CGCTGTCATCCTCATTGCTA	GAGCACTTGCCACTGGTGTA	this study	
human PD-L1	GGTGGTGCCGACTACAAGCGA	CCTTGGGGTAGCCCTCAGCCT	Jaenisch Laboratory	PMID: 33205073
human CEACAM1	TGAATGTCACCTATGGCCCG	TGGGGCTTTGCTACTACTGG	this study	
human RACK7	AAGCGCCAGATTCGTAGCAGGT	TCCTCCGAATCGCTGTGCTCTA	this study	
human KDM5C	ACTGCTGACCATTGCTGAACGC	CCTCCTTGAGAGCCTGGATGTT	this study	
human PS2	CCAGTGTGCAAATAAGGGCTGC	AGGCAGATCCCTGCAGAAGTGT	this study	
human GAPDH	CTGACTTCAACAGCGACACC	CTGACTTCAACAGCGACACC	this study	
**Oligonucleotides (mitochondrial DNA RT-qPCR)**				
human ND1	ATTCGATGTTGAAGCCTGAGACT	TGACCCTTGGCCATAATATGATT	this study	
human ATP6	TAGCCATACACAACACTAAAGGACGA	GGGCATTTTTAATCTTAGAGCGAAA	this study	
human CYB	ATCACTCGAGACGTAAATTATGGCT	TGAACTAGGTCTGTCCCAATGTATG	this study	
**Oligonucleotides (genomic DNA RT-qPCR)**				
human HBB	ACCTCAAGGGCACCTTTGC	AAAACATCAAGCGTCCCATAGAC	this study	
**Oligonucleotides (shRNA-knockdown)**				
Target	**Sequence (5' to 3')**		**Source**	**Identifier**
human RACK7	CCGGCCGGATTTCCTTGTCGGATATCTCGAGATATCCGACAAGGAAATCCGGTTTTTG		RNAi Core, Academia Sinica	TRCN0000037995
mouse RACK7	CCGGGCCTGGGTTCCAGTCAATAATCTCGAGATTATTGACTGGAACCCAGGCTTTTTG		RNAi Core, Academia Sinica	TRCN0000088516
